# A dedicated microarray for in-depth analysis of pre-mRNA splicing events: application to the study of genes involved in the response to targeted anticancer therapies

**DOI:** 10.1186/1476-4598-13-9

**Published:** 2014-01-15

**Authors:** Marine Pesson, Béatrice Eymin, Pierre De La Grange, Brigitte Simon, Laurent Corcos

**Affiliations:** 1 UMR INSERM U1078-UBO, Equipe ECLA, Faculté de Médecine, 22 Avenue Camille Desmoulins, 29200 Brest, France; 2 Centre de Recherche INSERM U823, Université Joseph Fourier, Equipe 2 Bases Moléculaires de la Progression des Cancers du Poumon, Institut Albert Bonniot, Domaine de la Merci, Rond-Point de la Chantourne, 38706 La Tronche Cedex, France; 3 IUH, Centre Hayem, 1 Avenue Claude Vellefaux, 75010 Paris, France

**Keywords:** DNA chip, Targeted anticancer therapies, Pre-mRNA splicing, SRSF2

## Abstract

Alternative pre-mRNA splicing (AS) widely expands proteome diversity through the combinatorial assembly of exons. The analysis of AS on a large scale, by using splice-sensitive microarrays, is a highly efficient method to detect the majority of known and predicted alternative transcripts for a given gene. The response to targeted anticancer therapies cannot easily be anticipated without prior knowledge of the expression, by the tumor, of target proteins or genes. To analyze, in depth, transcript structure and levels for genes involved in these responses, including *AKT1-3*, *HER1-4*, *HIF1A*, *PIK3CA*, *PIK3R1-2*, *VEGFA-D* and *PIR*, we engineered a dedicated gene chip with coverage of an average 185 probes per gene and, especially, exon-exon junction probes. As a proof of concept, we demonstrated the ability of such a chip to detect the effects of over-expressed SRSF2 RNA binding protein on the structure and abundance of mRNA products in H358 lung cancer cells conditionally over-expressing SRSF2. Major splicing changes were observed, including in *HER1/EGFR* pre-mRNA, which were also seen in human lung cancer samples over-expressing the SRSF2 protein. In addition, we showed that variations in *HER1/EGFR* pre-mRNA splicing triggered by SRSF2 overexpression in H358 cells resulted in a drop in HER1/EGFR protein level, which correlated with increased sensitivity to gefitinib, an EGFR tyrosine kinase inhibitor. We propose, therefore, that this novel tool could be especially relevant for clinical applications, with the aim to predict the response before treatment.

## Background

Alternative pre-mRNA splicing (AS) occurs for an estimated 90% of genes in the human genome [1], with remarkable repercussions on proteome diversity [2]. The outcome of AS strongly depends on context. Hence, AS occurs to allow the onset of development or differentiation programs, to participate in cancer occurrence or progression, and to develop integrated responses to stressful conditions [3-5]. Importantly, AS transcripts may encode alternative protein isoforms, which quite often display distinct or even opposite functions, such as for the pro- or anti-apoptotic caspases or Bcl-2 family proteins [6-8]. In addition, AS may also lead to the assembly of short-lived mRNAs targeted to degradation through the nonsense mediated decay (NMD) system [9]. However, even if NMD transcripts do not encode proteins, their occurrence may modify the ratio of mRNA isoforms, potentially affecting protein synthesis outcome [10].

Analytical tools to study AS on a large scale have been developed by Affymetrix™, with the Human Exon 1.0 ST arrays, also referred to as splice-sensitive microarrays, which allow surveying known and predicted AS events throughout the transcriptome [11,12]. Recently, deep sequencing methods have made it possible to determine both mRNA levels and structure [13-15]. Nevertheless, the mathematical tools necessary to decipher the structure and amount of mRNA species identified by sequencing are still under constant development [16,17]. In addition, a recent comparison between RNA-Seq and Affymetrix™ Exon arrays has revealed that the chip method was more powerful at detecting and quantifying exons [18]. It was also demonstrated that microarray technologies could be used as a reliable routine diagnostic tool, thanks to the development of a small custom-made microarray able to predict disease outcome in breast cancer patients [19]. Following on that path, the aim of the present study was to develop a customized microarray enabling to detect both known and predictable AS events for a small number of genes involved in tumor growth and in the response to targeted anticancer therapies. To take advantage of the DNA chip experimental setup, we wished to improve the methodology by increasing the amount of probes, including exon-exon junction probes absent from Affymetrix™ Exon arrays, which would allow detecting virtually all AS events that could occur in this subset of genes.

Targeted anticancer therapies include drugs, such as inhibitors of tyrosine kinase or monoclonal antibodies (mAbs), which oppose cell growth signaling or tumor blood vessel development, promote the specific death of cancer cells, or stimulate the immune system. Among specific molecules with which targeted therapies interfere, the HER (human epidermal growth factor receptor) family regulates cell growth, survival, adhesion, migration and differentiation. Trastuzumab (Herceptin™), which was FDA-approved in 2000, was the first treatment using a humanized mAb to target the receptor tyrosine kinase encoded by the *HER2* oncogene, and is mainly used to treat breast cancers over-expressing this receptor [20,21]. Cetuximab (Erbitux™) and gefitinib (Iressa™) target HER1/EGFR (epithelial growth factor receptor), or its tyrosine kinase activity, respectively, and bevacizumab (Avastin™) blunts VEGF-A (vascular endothelial growth factor A) activity upon binding to the Gly88 residue from the extracellular domain [22]. AS transcript variants have been characterized for all these targets, especially for *VEGFA*[23-25], and could account for part of the inefficacy of the responses to mAbs. The PIK3/Akt pathway is a major signaling cascade downstream of the receptor tyrosine kinases. In addition, *VEGFA* expression is regulated by the hypoxia factor HIF-1α. The analyzed genes on this custom microarray include *AKT1-3*, *HER1-4*, *HIF1A*, *PIK3CA*, *PIK3R1-2*, *VEGFA-D*, and *PIR* that lies close to the *VEGFD* locus and could be fused to *VEGFD* upon read through transcription. Collectively, these genes can lead to the assembly of more than 100 mRNAs with protein-coding capacity ( http://www.ensembl.org). Hence, the response to targeted anticancer therapy will likely depend, at least in part, on the selection of specific combinations of protein targets derived from AS events.

In order to validate our custom DNA chip, we took advantage of the human lung adenocarcinoma H358 cell line that we previously engineered to conditionally over-express the pre-mRNA splicing enhancer protein SRSF2, which controls the splicing of *VEGFA* pre-mRNA [26], but also has a role in transcriptional elongation [27]. Positive results were further validated by specific quantitative RT-PCR in both H358 cells and human non-small cell lung carcinoma (NSCLC) samples that we previously showed to over-express the SRSF2 protein [28]. The repercussion of altered splicing on the amount of the HER1/EGFR protein and the response to gefitinib were analyzed in H358 cells.

## Results

### Validation of the splice-inducing ability of SRSF2

Using an E1A-based plasmid minigene in transient transfection experiments, we analyzed the splice-inducing ability of SRSF2 (Additional file 1: Figure S1). There was an up-regulation of the 13S PCR band associated with a down-regulation of the 9S band, indicating that SRSF2 over-expression could modify the balance of E1A-derived transcripts, as originally described [29].

### Cross validation with 44 k Agilent microarray

To analyze the gene expression changes triggered by over-expression of SRSF2 in H358 lung cancer cells, we performed an analysis using 44 k Agilent™ microarrays. These data have been deposited in NCBI’s Gene Expression Omnibus and are accessible through GEO Series accession number GSE50467. A lot of genes were differentially expressed between SRSF2-over-expressing H358 lung cancer cells and H358 control cells (1,709 deregulated probes; ≥ 2.0 FC, P-value ≤ 0.05 by *t*-test with FDR; Additional file 2: Table S1), corresponding to 52% up- and 48% down-regulations. Hence, in addition to its already reported role in the regulation of *VEGFA* splicing, over-expression of SRSF2 led to the regulation of transcript abundance of many additional genes, including genes present on the 15 k custom chip (Additional file 3: Table S2), as demonstrated with the 44 k Agilent™ microarrays.

### Validation of the labeling method: comparison of the 15 k custom and 44 k Agilent microarrays

The labeled cRNA yield and the specific activity of cyanine3 were examined for each of three labeling experiments (Additional file 4: Table S3). A comparison of the 15 k custom and 44 k commercial microarrays, with respect to Agilent™ probes present on both chips, was performed in order to validate the use of the labeling method with the 15 k custom microarray. The number of 15 k replicates using Quick Amp labeling was equal to 4 for each condition (control or SRSF2 over-expression), and the number of 44 k replicates was equal to 6 for each condition. We found that 313 Agilent™ probes (corresponding to 16% of the total number of Agilent™ probes on the 15 k chip) were deregulated on the 15 k custom microarray (≥ 1.5 FC, P-value ≤ 0.05), among which 310 (99%) had the same type of (up- or down-) regulation on the 44 k commercial microarrays (Additional file 5: Table S4). Pearson correlation between expression signals of these 313 common genes led to a coefficient of 0.89. Therefore, it was considered that Quick Amp labeling was validated for the 15 k custom microarray.

### Detection of the mRNA regulation

We analyzed the expression of the 16 selected genes present in the 15 k custom microarray, considering the expression of all custom probes for each gene (Table 1). Four genes (*HER4*, *PIK3CA*, *PIK3R1* and *VEGFD*) were not expressed; five genes (*AKT2*, *AKT3*, *HER2*, *PIK3R2* and *VEGFC*) were not differentially expressed; five genes (*AKT1*, *HER3*, *HIF1A*, *PIR* and *VEGFB*) were slightly down-regulated (≤ 1.5 FC, P-value ≤ 0.05); *HER1/EGFR* was more strongly down-regulated (≥ 1.5 FC, P-value ≤ 0.05), and *VEGFA* was up-regulated (≥ 1.5 FC, P-value ≤ 0.05) in SRSF2-over-expressing H358 lung cancer cells in comparison to H358 control cells. A good concordance between the 15 k and 44 k microarray results was found: 8 out of the 16 genes present in 15 k custom chip were deregulated on 44 k chips (≥ 1.1 FC, P-value ≤ 0.05), considering Agilent™ probes, and showed the same type of regulation on the 15 k chip, considering custom probes (Additional file 3: Table S2).

**Table 1:** Gene expression changes in SRSF2-over-expressing H358 lung adenocarcinoma cells

**Gene regulation**	**Gene symbol**	**Control condition intensity**	**SRSF2 condition intensity**	**Regulation**	**Fold-change**	**P-value**
Up-regulated	*VEGFA*	9.25	9.99	up	1.67	3.38E-08
Down-regulated	*HER1/EGFR*	5.01	4.11	down	1.87	3.65E-06
Slightly down-regulated	*HER3*	2.33	1.78	down	1.47	3.98E-03
	*HIF1A*	6.48	6.06	down	1.33	2.04E-04
	*PIR*	7.01	6.62	down	1.32	1.82E-03
	*AKT1*	8.04	7.68	down	1.28	8.15E-05
	*VEGFB*	7.84	7.48	down	1.28	2.56E-04
Not regulated	*PIK3R2*	3.94	4.19	up	1.19	1.62E-01
	*AKT3*	2,00	1.91	down	1.06	6.18E-01
	*HER2*	4.89	4.81	down	1.05	4.01E-01
	*AKT2*	5.81	5.74	down	1.05	5.57E-01
	*VEGFC*	5.90	5.89	down	1.01	9.03E-01
Not expressed	*PIK3R1*	1.35	1.89	up	1.45	3.71E-02
	*VEGFD*	0.88	0.98	up	1.07	8.23E-02
	*HER4*	0.65	0.69	up	1.03	4.87E-01
	*PIK3CA*	1.79	1.78	down	1.01	8.58E-01

The expression and the regulation of the 16 genes were analyzed on the 15 k custom microarray in SRSF2-over-expressing H358 lung cancer cells in comparison to control cells. Some genes were not expressed; others were not differentially expressed. Five genes were slightly down-regulated (≤ 1.5 FC, P-value ≤ 0.05), and one gene (*HER1/EGFR*) was more strongly down-regulated (≥ 1.5 FC, P-value ≤ 0.05). Only one gene (*VEGFA*) was up-regulated in the SRSF2 over-expression condition (≥ 1.5 FC, P-value ≤ 0.05).

### Regulation events among the expressed genes

The bioinformatics analysis of the 15 k custom microarray showed that 30 custom probe sets from expressed genes were differentially expressed in SRSF2-over-expressing H358 lung cancer cells in comparison to H358 control cells (≥ 1.5 FC, P-value ≤ 0.05; Table 2). The low expressed deregulated probe sets were not considered. The regulation events corresponded to 70% down- and 30% up-regulations, mostly affecting cassette exons, but also 5′-untranslated regions and terminal or donor splice sites, of 9 genes among the 12 expressed genes (*AKT2*, *AKT3*, *HER1/EGFR*, *HER2*, *HER3*, *HIF1A*, *PIK3R2*, *VEGFA* and *VEGFB*). Regulations were associated with a high, medium or low confidence, depending on the regulation of probes close to the deregulated probe sets. A list of supporting evidences (Additional file 6: Table S5) was defined corresponding to the regulations that were not always statistically relevant, but confirmed the deregulation of some probe sets. Consequently, these regulations were associated with a high confidence. On the contrary, the confidence was considered as low if neighboring probes were not deregulated or if their regulation was opposite. The regulations associated with a high fold-change and corresponding to unknown and predicted pre-mRNA splicing events could be of special interest.

**Table 2:** Deregulated probe sets in SRSF2-over-expressing H358 lung adenocarcinoma cells

**Gene symbol**	**Region name**	**Region type**	**Confidence**	**Regulation**	**Fold-change**	**P-value**	**RT-PCR**
*AKT2*	je14_e15_5p_region	Junction	Low	Down	1.63	1.36E-02	No
***AKT3***	**je7_e8**	**Junction**	**High**	**Down**	**5.99**	**2.07E-05**	**Yes**
***AKT3***	**e8**	**Exon**	**High**	**Down**	**4.19**	**4.10E-03**	**Yes**
***HER1/EGFR***	**je16_e19**	**Junction**	**High**	**Down**	**3.66**	**1.42E-03**	**Yes**
*HER1/EGFR*	predict_exon_1_2-10	Exon	Medium	Up	33.98	0.00E + 00	No
*HER1/EGFR*	est_1_2	Exon	Medium	Up	17.35	1.00E-09	No
*HER1/EGFR*	predict_exon_2_3-1	Exon	Medium	Up	4.97	5.51E-05	No
*HER1/EGFR*	e26	Exon	Medium	Down	2.64	2.30E-03	No
*HER1/EGFR*	ae1_donor_alter	Donor_alter	Medium	Up	5.55	5.40E-03	No
*HER1/EGFR*	je14_e15	Junction	Low	Down	1.87	1.05E-02	No
*HER1/EGFR*	je12_e13	Junction	Low	Down	1.76	1.13E-02	No
*HER2*	ae10_prom_alter	Prom_alter	Medium	Down	2.73	2.32E-02	No
*HER3*	je22_ae23_acceptor_alter_l	Junction	Low	Up	1.68	3.33E-02	No
***HIF1A***	**je10_e11**	**junction**	**High**	**Down**	**1.87**	**1.66E-05**	**Yes**
***HIF1A***	**e10**	**Exon**	**High**	**Down**	**1.62**	**3.65E-02**	**Yes**
***HIF1A***	**e9**	**Exon**	**High**	**Down**	**1.56**	**4.78E-02**	**Yes**
*HIF1A*	e5	Exon	Low	Down	2.07	1.23E-02	No
*HIF1A*	je1_5p_region_ae3_acceptor_alter	Junction	Low	Down	1.56	1.64E-02	No
*HIF1A*	je14_e16	Junction	Low	Up	2.22	2.08E-02	No
*PIK3R2*	e6	Exon	High	Down	1.97	7.61E-05	No
*PIK3R2*	je5_e6	Junction	High	Down	3.95	3.98E-04	No
*PIK3R2*	je7_e8	Junction	Low	Down	1.68	2.14E-03	No
*PIK3R2*	e9	Exon	Low	Down	1.74	3.34E-03	No
*PIK3R2*	je2_e3	Junction	Low	Down	1.58	4.36E-02	No
***VEGFA***	**e4_term_alter**	**Term_alter**	**High**	**Up**	**10.18**	**7.43E-08**	**Yes**
***VEGFA***	**ae6_donor_alter_2**	**Donor_alter**	**High**	**Down**	**2.44**	**4.18E-07**	**Yes**
***VEGFA***	**jae6_donor_alter_2_e7**	**Junction**	**High**	**Down**	**1.93**	**4.96E-03**	**Yes**
***VEGFA***	**ae7_donor_alter**	**Donor_alter**	**High**	**Up**	**1.80**	**6.98E-04**	**Yes**
*VEGFA*	e7	Exon	Low	Up	1.50	4.00E-04	No
*VEGFB*	je2_e3	Junction	Low	Down	1.62	6.47E-05	No

A list of the 30 differentially expressed and deregulated custom probe sets (≥ 1.5 FC, P-value ≤ 0.05) from expressed genes among the 16 analyzed genes in SRSF2-over-expressing lung cancer cells in comparison to control cells on the 15 k custom microarray is presented. The regulations were associated with a high, medium or low confidence, depending on the regulation of probes in the vicinity of the deregulated probe sets. According to the results with a high confidence (in bold characters), we expect an up-regulation of exon 7 and a down-regulation of exon 8 for *AKT3*, a multiple exon skipping for *HER1/EGFR*, a skipping of both exons 9 and 10 for *HIF1A*, an alternative polyadenylation in intron 4, and alternative donor sites for exons 6 and 7 for *VEGFA*.

### Validation of regulation events by real-time polymerase chain reaction

**Figure 1:**
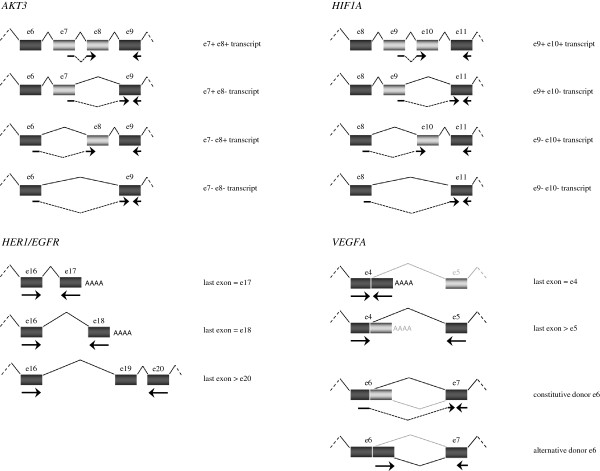
**Alternative splicing events induced by SRSF2 over-expression in H358 lung adenocarcinoma cells.** The *AKT3*-derived mRNAs in the exon 6–9 region, the *HIF1A*-derived mRNAs in the exon 8–11 region, and the various last exons for *HER1/EGFR* and *VEGFA* are depicted. The arrows show the position of the primers designed and used for validation of the splicing events detected by the 15 k custom gene chip.

Quantitative RT-PCR was used to measure the expression of 9 genes deregulated on both the 15 k custom and the 44 k commercial microarrays, and the differential expression of all genes in SRSF2-over-expressing H358 lung cancer cells in comparison to H358 control cells was analyzed with RNA isolated independently from that used for chip hybridization (Additional file 7: Table S6). These results confirmed the validity of our experimental approach used to analyze the 15 k custom microarray. Ten out of the 30 deregulated probe sets were selected according to their high confidence (Table 2), and concerned 4 genes, including *AKT3*, *HER1/EGFR*, *HIF1A* and *VEGFA* (Figure 1). The results of quantitative RT-PCR experiments are shown in Table 3. Relative mRNA levels were normalized to control gene mRNA levels or a fold-change was calculated comparing to a reference event. For *HER1/EGFR*, we showed a down-regulation of one of the transcripts (last exon > e20) in SRSF2-over-expressing H358 lung cancer cells in comparison to H358 control cells. For *AKT3*, we validated the up-regulation of exon 7 and the down-regulation of exon 8; that is because the e7+/e8- transcript was over-expressed as compared to the e7+/e8+ transcript including both exons. For *HIF1A*, the up-regulation for two (e9+/e10- and e9-/e10-) of the three alternative transcripts compared to the e9+/e10+ transcript led us to conclude that both exons 9 and 10 were down-regulated. For *VEGFA*, we validated the alternative polyadenylation in intron 4 by an over-expression of the smaller transcript (last exon = e4) in comparison to the longer transcript (last exon > e5). We also confirmed the alternative donor site for the exon 6 by an up-regulation of the "alternative donor e6" transcript in comparison with the "constitutive donor e6" transcript.

**Table 3:** Quantitative RT-PCR validation in SRSF2-over-expressing H358 lung adenocarcinoma cells

**SRSF2 Condition *vs*. Control condition**					
Gene	Calculation	Transcript	Expression	Observed transcript regulation	Expected transcript regulation
*HER1/EGFR*	Relative expression	Last exon = e17	n/a	Not expressed	No expression
		Last exon = e18	0.91	Not regulated	Over-expression
		Last exon > e20	0.42	Under-expressed	Under-expression
*AKT3*	Fold-change	e7+ e8- *vs*. e7+ e8+	1.46	Over-expression of e7+ e8-	Over-expression of exon 7 and under-expression of exon 8
		e7- e8+ *vs*. e7+ e8+	-1.19	No regulation of e7- e8+	
		e7- e8- *vs*. e7+ e8+	n/a	No expression of e7- e8-	
*HIF1A*	Fold-change	e9+ e10- *vs*. e9+ e10+	1.91	Over-expression of e9+ e10-	Under-expression of exon 10
		e9- e10+ *vs*. e9+ e10+	-1.52	Under-expression of e9- e10+	Under-expression of exon 10
		e9- e10- *vs*. e9+ e10+	1.82	Over-expression of e9- e10-	Under-expression of exons 9 and 10
*VEGFA*	Fold-change	Last exon = e4 *vs*. last exon > e5	18.93	Over-expression of "last exon = e4"	Over-expression of exon 4
*VEGFA*	Fold-change	Alternative *vs*. constitutive donor e6	14.46	Over-expression of "alternative donor e6"	Over-expression of alternative donor

The regulation of the 10 selected deregulated custom probe sets was analyzed by quantitative RT-PCR in SRSF2-over-expressing lung cancer cells in comparison to control cells. Relative mRNA levels were normalized to that of *beta*-2-microglobulin or a fold-change was calculated comparing to a reference event. The cut-off value was equal to 1.40. n/a: not available.

### HER1/EGFR protein expression analysis

**Figure 2:**
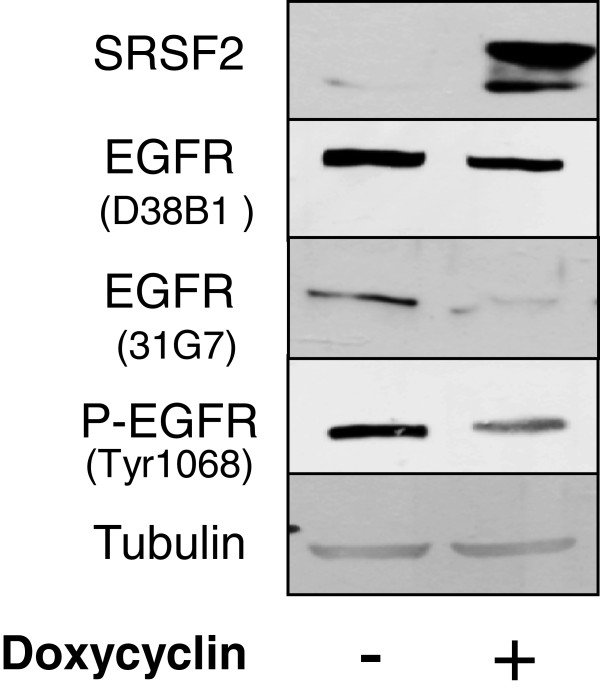
**Western blot analysis of HER1/EGFR expression.** HER1/EGFR and P-HER1/EGFR (Tyr1068) protein levels were analyzed in H358/Tet-On/SRSF2 inducible H358 clone by western blotting. Tubulin was used as a loading control.

The 15 k custom microarray predicted multiple exon skipping in the 3′ region of *HER1/EGFR* in SRSF2-over-expressing H358 lung cancer cells, which was confirmed by quantitative RT-PCR. These observations led us to test whether these splicing events would have an impact on the amount of the HER1/EGFR protein. Western blotting analysis was performed using various anti-EGFR antibodies directed against the N-terminal (31G7) or the C-terminal (D38B1) portion of the protein, as well as against the phosphorylated active form of EGFR (P-HER1/EGFR-Tyr1068). The results demonstrated that SRSF2 overexpression in H358 cells led to a decrease in EGFR protein amount, as detected using all antibodies (Figure 2). These data suggested that SRSF2-regulated *EGFR* pre-mRNA splicing strongly affects EGFR protein expression.

**Figure 3:**
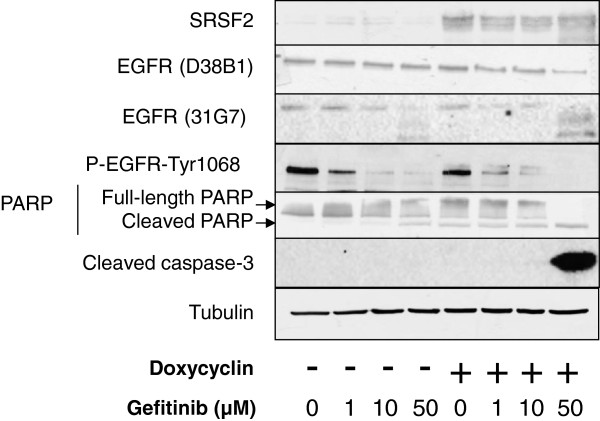
**Effects of SRSF2 overexpression on the response to gefitinib in H358 lung adenocarcinoma cells.** The H358/Tet-On/SRSF2 inducible H358 clone was treated or not for 24 hours with increasing concentrations of gefitinib as indicated, in the presence (+) or absence (-) of 1 μg/mL doxycyclin. Western blot analysis was performed using the indicated antibodies. Apoptosis was analyzed by the detection of PARP or caspase-3 activation. Tubulin was used as a loading control.

In addition, H358 cells express a wild-type EGFR protein and are resistant to apoptosis in response to EGFR tyrosine kinase inhibitors such as gefitinib. In order to determine if SRSF2-induced EGFR protein down-regulation could modify the response of H358 cells to gefitinib, we performed a dose–response of the drug in the presence or absence of SRSF2 induction (Figure 3). As expected, a 24 hours-treatment with gefitinib significantly prevented EGFR-Tyr1068 phosphorylation in these cells, but only partially engaged apoptosis at the higher concentration, which was detected by poly-ADP ribose polymerase (PARP) processing. However, caspase-3 was never activated in gefitinib-treated cells. Of note, at the highest gefitinib concentration, a reduction in the amount of total EGFR together with the appearance of protein bands of smaller sizes was observed when using the 31G7 antibody mainly. These data suggested that EGFR could be processed in response to high gefitinib doses. Importantly, when SRSF2 was overexpressed in gefitinib-treated cells, the decrease in EGFR protein amount was more pronounced and apoptosis was strongly engaged, as evidenced by procaspase-3 and PARP cleavages (Figure 3). This result indicated that SRSF2, through its ability to control EGFR protein expression, sensitizes H358 cells to the apoptosis induced by EGFR tyrosine kinase inhibitors.

### Alternative splicing events in lung cancer biopsy samples

Finally, we aimed at extending some of our *in vitro* data to cancer tissues. For this purpose, we took advantage of the cancer-associated over-expression of SRSF2, as it may occur in NSCLC [28]. SRSF2 and phospho-SRSF2 expression scores (0–300) were established in 10 NSCLC biopsy samples (Table 4A) by multiplying the percentage of labeled tumor cells (0 to 100%) by the staining intensity (0, null; 1, low; 2, moderate; 3, strong). Interestingly, the three NSCLC samples with the highest SRSF2 and phospho-SRSF2 scores all displayed a drop in the *HER1/EGFR* "last exon > e20" transcript, as determined by quantitative RT-PCR, similarly to what occurred in lung cancer cells. We also analyzed the occurrence of the *AKT3*, *HIF1A* and *VEGFA* splicing events in NSCLC biopsy samples (Table 4B). For several samples, we observed an over-expression of exon 7 and an under-expression of exon 8 of *AKT3*, and an over-expression of exon 4 and alternative exon 6 donor splice site for *VEGFA*. Although the relationships between SRSF2 status and these splicing events were less clear in these cases, maybe owing to the small number of samples, these data validated, in cancer samples, some of the pre-mRNA splicing events detected in the SRSF2-over-expressing H538 cell line. The results were inconclusive for *HIF1A*, possibly reflecting heterogeneity among the NSCLC samples with respect to expression of this gene.

**Table 4:** Quantitative RT-PCR validation in non small cell lung carcinoma samples

**A**													
**Sample**			**1**	**2**	**3**	**4**	**5**	**6**	**7**	**8**	**9**	**10**	
SRSF2 Protein Score			**270**	**270**	**240**	180	160	140	120	100	60	n/a	
Phospho-SRSF2 Protein Score			**270**	**270**	**270**	100	100	90	60	100	40	n/a	
**B**													
			**Non small cell lung carcinoma *vs*. normal lung tissue**										
Gene	Calculation	Transcript	**1**	**2**	**3**	4	5	6	7	8	9	10	Observed Transcript Regulation
*HER1/EGFR*	Relative expression	Last exon = e17	**n/a**	**n/a**	**n/a**	n/a	7.17	n/a	n/a	16.15	2.52	n/a	Not expressed or over-expressed
		Last exon = e18	**2.69**	**n/a**	**1.86**	2.27	5.13	6.86	2.23	15.16	3.01	66.18	Over-expressed
		Last exon > e20	**0.15**	**0.29**	**0.20**	6.71	6.99	21.45	0.10	5.39	2.46	n/a	Over-expressed or under-expressed
*AKT3*	Fold-change	e7+ e8- *vs*. e7+ e8+	**0.84**	**1.46**	**1.16**	1.37	1.08	1.76	1.29	1.47	1.47	2.28	Over-expression or no regulation
		e7- e8+ *vs*. e7+ e8+	**n/a**	**1.33**	**n/a**	1.35	n/a	n/a	n/a	0.38	n/a	n/a	Low expression of e7- e8+
		e7- e8- *vs*. e7+ e8+	**n/a**	**n/a**	**n/a**	n/a	n/a	n/a	n/a	n/a	n/a	n/a	No expression of e7- e8-
*HIF1A*	Fold-change	e9+ e10- *vs*. e9+ e10+	**0.63**	**0.54**	**1.63**	1.40	0.79	1.77	0.87	1.22	1.20	1.15	Depending on biopsy samples
		e9- e10+ *vs*. e9+ e10+	**1.87**	**n/a**	**n/a**	1.06	n/a	n/a	n/a	n/a	1.14	1.77	Depending on biopsy samples
		e9- e10- *vs*. e9+ e10+	**0.57**	**0.73**	**1.55**	1.49	0.84	1.88	0.79	1.99	0.76	1.01	Depending on biopsy samples
*VEGFA*	Fold-change	Last exon = e4 *vs*. Last exon > e5	**1.33**	**1.21**	**1.54**	1.92	0.84	2.22	10.37	2.73	1.21	1.09	Over-expression or no regulation
*VEGFA*	Fold-change	Alternative *vs*. constitutive donor e6	**3.50**	**3.33**	**5.25**	3.99	n/a	n/a	19.22	14.29	n/a	45.68	Over-expression

The regulation of the 10 selected deregulated custom probe sets was analysed by quantitative RT-PCR in 10 non small cell lung carcinoma – normal sample pairs (patients numbered from 1 to 10). SRSF2 protein expression levels in biopsy samples were analysed by immunohistochemistry in a previous study. A score (0–300) was established for SRSF2 and phosphorylated SRSF2 (P-SRSF2). Patients with scores ≥ 150 and > 175 were those over-expressing SRSF2 and P-SRSF2 proteins respectively, as compared to normal lung tissues. Patients in bold characters over-expressed both proteins. n/a: not available.

Relative mRNA levels were normalised to that of *beta*-2-microglobulin or a fold-change was calculated comparing to a reference event. The cut-off value was equal to 1.40. n/a: not available. Patients in bold characters over-expressed both SRSF2 and phospho-SRSF2 proteins (see Table 4A).

## Discussion

In this study, we designed a custom gene expression microarray amenable to the study of alternative pre-mRNA splicing (AS) events of a selection of genes involved in the response to targeted anticancer therapies. This approach was preferred to commercial microarrays, such as the Human Exon 1.0 ST arrays (Affymetrix™) because it allowed a deeper analysis of AS, in this case of a small number of genes highly relevant from a clinical standpoint. Indeed, it is clear that our custom splice-sensitive microarray could theoretically detect many more events than Affymetrix™ Exon Arrays (Table 5), considering probe length, probe number and, especially, exon-exon junction probes, which were not present on Affymetrix™ Exon Arrays. At a practical level, several high confidence events revealed, thanks to exon-exon junction probes, specific splicing events (Table 2). For example *AKT3* je7_e8, *HER1/EGFR* je16_e19 or *HIF1A* je10_e11 junction-specific events would have been undetected on Affymetrix™ arrays. In addition, selecting only the high confidence events, the regulations observed through the chip analysis were confirmed by quantitative RT-PCR, emphasizing the robustness of both the technical and the analytical tools used in this study. Nevertheless, we anticipate that RNA-Seq methodologies will probably soon be another, reliable, means for characterizing AS throughout the transcriptome [30,31].

**Table 5:** Comparison of gene coverage between the custom gene chip and the Affymetrix™ Exon Array

**Gene**	**Custom array (present study)**		**Affymetrix™ Exon array**	
	**Nb probes (exonic/junction)**	**Average probe length (bp)**	**Nb probes (exonic/junction)**	**Average probe length (bp)**
HIF1A	123 (85/38)	42.4	80 (80/0)	25.0
VEGFA	90 (64/26)	42.3	60 (60/0)	25.0

The numbers of probes and their average length are shown for both the HIF1A and VEGFA genes.

We are aware of only one study that used a designed chip to analyze the occurrence of splicing variants which, in that case, corresponded to AS events from a single gene, *CIZ1*, encoding a Cip1-interacting zinc finger protein [32]. This approach led to the identification of a splice variant that may be specific for pediatric cancer. There is an absolute need for predictive biomarkers of therapeutic responses, especially targeted anticancer therapies, as many patients do not respond or acquire resistance. For instance, VEGF-A isoforms may not respond identically to anti-VEGF-A mAbs (bevacizumab). In fact, the co-occurrence of both pro-angiogenic (VEGF-A_xxx_) and anti-angiogenic (VEGF-A_xxx_b) splice isoforms might restrict the therapeutic response [33-37]. In addition, the occurrence of soluble EGFR isoforms, as detected in meningiomas [38], presumably unresponsive to tyrosine kinase inhibitor therapy, might also dampen the therapeutic response. Furthermore, an exon 4-lacking EGFR variant mRNA was associated with an increased metastatic potential, a molecular event that would likely have been detected with our splice-sensitive microarray [39]. Hence, in addition to providing a comprehensive picture of splicing events and potential therapy response, our chip could also help predicting clinical outcome, based on the detection of pro-metastatic mRNA species. Nevertheless, beyond the concept, more predictive studies should be performed to make our splice-screening methodology an efficient therapy selecting option.

We showed that SRSF2 has an effect on transcriptional regulation and on AS of several genes analyzed in this study. Notably, SRSF2 over-expression modified *HER1/EGFR* and *VEGFA* expression in H358 lung cancer cells. Using patient-derived material, we observed that strong SRSF2 over-expression in NSCLC is associated with splicing alterations of the *HER1/EGFR* and *VEGFA* transcripts, as predicted from the results in the SRSF2-over-expressing H358 lung cancer cell line. In addition, *HER1/EGFR* splicing events have also been identified in lung adenocarcinomas [40], lending support to our results. The observation that the increase in SRSF2 protein level induced massive procaspase-3 cleavage when associated with gefitinib in H358 cells, which express wild-type and non amplified EGFR protein, may be particularly relevant for patients with lung adenocarcinomas without EGFR mutations, as one of the challenges is to understand why only some of them respond to EGFR tyrosine kinase inhibitors.

The expression level of *HER1* mRNA, measured through analysis of the 44 k Agilent™ chip, and the western blotting analysis of the protein, showed a good correlation in response to SRSF2 over-expression. In this specific case, use of the custom 15 k chip would not have been more predictive. Nevertheless, it is doubtless that AS, analyzed globally for all genes from the chip, will provide a lot more information on both transcript abundance and structure, allowing defining a prognostic indicator of response to antibody-based therapy [41]. An important challenge will be to develop specific antibodies to detect full length or modified proteins encoded by AS-derived transcripts. Alternatively, mass spectrometry proteomics could be used to identify and quantify such proteins [42]. The custom chip analysis could thus ideally supplement immunology- or proteomics-based approaches aimed at looking for the expression of protein targets. Our DNA gene chip could also be used to analyze the effect of other triggers, such as over-expression or silencing of other splice-modifying proteins, or treatment with drugs, especially anticancer drugs, which can profoundly affect pre-mRNA splicing [3,43].

## Conclusion

Our results describe, for the first time, the design and validation of a custom splice-sensitive microarray to detect AS events occurring in genes involved in the response to targeted anticancer therapies. Such an experimental setup could help clinicians choose anticancer drugs depending on the tumor expression of gene targets with proficient mRNA structures.

## Methods

### Custom microarray design

A custom microarray was designed taking advantage on the 15 k Whole Human Genome microarray, available from Agilent™ (Agilent, Massy, France). Among the Agilent™ probes initially loaded on the chip, 11,881 (Additional file 8: Figure S2) were substituted by custom oligonucleotides, corresponding to known and predicted exons, introns and junctions of 16 selected genes, among which there were members of the AKT (*AKT1*, *AKT2*, *AKT3*), HER (*HER1/EGFR*, *HER2*, *HER3*, *HER4*), PIK3 (*PIK3CA*, *PIK3R1*, *PIK3R2*) and VEGF (*VEGFA*, *VEGFB*, *VEGFC*, *VEGFD*) families, but also *HIF1A* and *PIR*. On the microarray, the majority (60%) of custom probes had a length of 40 bp; some were shorter (down to 22 bp; 8%); others were longer (up to 50 bp; 26%), which was mostly the case of the probes for exon-exon junctions. This was especially important to insure a good detection of alternative 5′ and 3′ splice sites, *i.e.* alternative exon boundaries. Each custom probe length was adjusted to 60 bp with linker addition. The other 3,863 probes on the microarray corresponded to replicates of commercial Agilent™ probes (genes or controls). As a whole, the expression of 1,967 distinct genes can be analyzed with our chip.

### Cell culture and RNA extraction

The H358 human lung adenocarcinoma cell line was cultured as described previously [44]. The H358/Tet-On/SRSF2 inducible clone, conditionally over-expressing the SRSF2 splicing factor under the control of a Tet-responsive promoter, has been described previously [44,45]. SRSF2 over-expression was induced upon 24 hours treatment with 1 μg/mL doxycycline (Additional file 9: Figure S3). Gefitinib was added to the cells at the indicated final concentrations for 24 hours. Total RNA was isolated using the Trizol reagent (Invitrogen, Cergy-Pontoise, France), according to the manufacturer’s instructions. RNA purity and integrity were determined by measuring the optical density ratio (A260/A280) and the RNA integrity number (RIN) using the RNA 6000 Nano LabChip (Agilent™) and the 2100 Bioanalyzer (Agilent™). Only RNA samples with a 28S/18S ratio > 1.0 and RIN ≥ 7.0 were used for microarray analyses.

### Plasmid transfection and minigene analysis

An E1A reporter minigene-containing plasmid (pXJ41-E1A) to study the effect of splice modifier proteins was used to further validate the effect of SRSF2 protein over-expression. The plasmid was transfected using Lipofectamine 2000 (Invitrogen). Cells were harvested 24 hours after transfection, and total RNA was extracted using the RNeasy Mini kit (Qiagen, Courtabœuf, France), according to the manufacturer’s instructions. The RNAs (200 ng) were further used for first-strand cDNA synthesis with the High-Capacity cDNA Reverse Transcription kit (Applied Biosystems, Courtabœuf, France). For the detection of E1A splice variants, PCR amplification was performed using primers 5′-TTT-GGA-CCA-GCT-GAT-CGA-AG-3′ and 5′-AAG-CTT-GGG-CTG-CAG-GTC-GA-3′, and PCR products were analyzed by agarose gel electrophoresis.

### Microarray hybridization

Analyses of the H358/Tet-On/SRSF2 mRNA content were performed on both the 15 k custom microarray and the 44 k Whole Human Genome microarray (Agilent™) that contains roughly 41,000 probes, providing full coverage of human transcripts. Double-stranded cDNA was synthesized from 500 ng of total RNA using the Quick Amp Labeling kit, One-color, as instructed by the manufacturer (Agilent™). Labeling with cyanine3-CTP, fragmentation of cRNA, hybridization and washing were performed according to the manufacturer’s instructions. The microarrays were scanned and the data were extracted with the Agilent™ Feature Extraction Software.

### Gene expression analysis

The bioinformatics analysis of the 15 k custom microarray data and the comparison of 15 k chip results with 44 k commercial chip results were performed by GenoSplice technology™. Concerning the 15 k custom microarray data analysis, data were normalized using median normalization based on Agilent™ control genes. Gene expression level was assessed using constitutive probes only (*i.e*., probes targeting regions that are not known to be alternative regions). For each gene of interest, all possible splicing patterns were defined and analyzed. All types of alternative events can be analyzed: alternative first exons, alternative terminal exons, cassette exons, mutually exclusive exons, alternative 5′ donor splice sites, alternative 3′ acceptor splice sites, and intron retentions. Analyses were performed using unpaired Student’s *t*-test on the splicing-index as previously described [46,47]. Results were considered statistically significant for unadjusted P-values ≤ 0.05 and fold-changes ≥ 1.5. After bioinformatics analysis of microarray data, a manual inspection using the GenoSplice EASANA™ interface was conducted to select high-confidence events. An alternative 44 k bioinformatics analysis was carried out. Raw gene expression data were imported into the GeneSpring GX 11.0.2 software program (Agilent™). Genes with missing values in more than 25% of the samples were excluded from the analysis. A 2-fold cut-off difference was applied to select the up- and down-regulated genes (P-value ≤ 0.05 by *t*-test with Benjamini-Hochberg false discovery rate).

### Real-time polymerase chain reaction analysis

Regulation events detected in the 15 k custom and 44 k commercial microarrays were analyzed by quantitative RT-PCR using RNA isolated from cell preparations separate from those originally used for microarray hybridization. Reverse transcription was performed as instructed by the manufacturer (Applied Biosystems), as described previously, and quantitative RT-PCR was conducted using the SYBR GREEN PCR Master Mix (Applied Biosystems), according to the manufacturer’s instructions, with an ABI 7300 real-time PCR system (Applied Biosystems). All determinations were performed in duplicate, normalized against *beta*-2-microglobulin or *GAPDH* as internal control genes. These reference transcripts were found to be stable when surveyed in several cell culture systems (data not shown). The results were expressed as the relative gene expression using the ∆∆Ct method [48]. The fold-change was also calculated comparing to a reference event. The sequences of the primers used for the 15 k custom microarray validation are presented in Additional file 10: Table S7.

### Protein extraction and western blotting analysis

The antibodies used in this study included anti-SRSF2 (4 F11) from Euromedex, anti-EGFR (31G7) from Invitrogen, anti-HER1/EGFR (D38B1) and anti-P-HER1/EGFR (Tyr1068) (D7A5) from Cell Signaling. For immunoblotting, cells were lysed in RIPA buffer [150 mM NaCl, 50 mM Tris HCl pH 8, 0.1% SDS, 1% Nonidet P40, 0.5% Na deoxycholate, 0.1 mM PMSF, 2.5 μg/mL pepstatin, 10 μg/mL aprotinin, 5 μg/mL leupeptin, 0.2 mM Na_3_VO_4_] for 30 minutes on ice and pelleted. Protein concentration was determined using the Biorad DC protein assay. Proteins (40–80 μg) were then separated in 10% SDS-PAGE gels and electroblotted onto PVDF membranes. Membranes were incubated overnight at +4°C with primary antibodies and proteins were detected using horseradish peroxidase-conjugated goat antibodies (Jackson Immunoresearch Laboratories, West Grove, PA, USA). After washing, the blots were revealed using the ECL chemiluminescence method (Amersham, Les Ulis, France). Tubulin was used as a loading control.

### Human samples

Tissue samples were collected from resection of lung tumors, and stored for scientific research in a biological resource repository (Centre de Ressources Biologiques, CHU Albert Michallon, Grenoble Hospital). National ethical guidelines were followed. All patients enrolled provided written informed consent. Tissue banking and research conduct was approved by the Ministry of Research (approval AC-2010-1129) and by the regional IRB (CPP 5 Sud Est). Protein and RNA samples were isolated and analysed as described above.

## Abbreviations

AS: Alternative pre-mRNA splicing; FC: Fold-change; FDR: Benjamini-Hochberg false discovery rate; mAbs: monoclonal antibodies; NMD: nonsense mediated decay; NSCLC: Non-small cell lung carcinoma.

## Competing interests

The authors declare that they have no competing interests.

## Authors’ contributions

MP performed the RT-PCR validation of microarray hybridization results, performed the E1A plasmid transfection and RT-PCR analysis, and drafted the manuscript. BS conducted the microarray experiments. BE developed the SRSF2-over-expressing lung cancer cells, and performed the western blotting experiments. PDLG designed the 15 k custom microarray, and performed the bioinformatics analysis of the data. LC coordinated the study, assisted with the design of experiments, and drafted the manuscript. All authors read and approved the final manuscript.

## Supplementary Material

Additional file 1: Figure S1 E1A splicing assay in response to SRSF2 over-expression. Following transient cell transfection with a SRSF2 expression plasmid, E1A splice-derived PCR products were electrophoresed through a 2% agarose gel and stained with ethidium bromide. The characteristic PCR products (9S-13S) are shown. Click here for file

Additional file 2: Table S1 Deregulated genes on the 44k Agilent^TM^ microarray in H358 SRSF2-over-expressing cells. Significantly down- and up-regulated genes in SRSF2-over-expressing H358 lung cancer cells in comparison to H358 control cells are listed (≥ 2.0 FC, P-value ≤ 0.05 by *t*-test with FDR). Click here for file

Additional file 3: Table S2 Regulation of the 16 selected genes on the 44k Agilent^TM^ microarray in SRSF2-over-expressing H358 cells. The results for the 8 deregulated genes in SRSF2-over-expressing H358 lung cancer cells in comparison to H358 control cells on the 44k microarrays are shown (≥ 1.1 FC, P-value ≤ 0.05 by *t*-test with FDR). The 44k microarray results for the 8 deregulated genes showed a good concordance with the 15k custom microarray results. Click here for file

Additional file 4: Table S3 Labeling efficiency for hybridization of the 15k custom microarray. The labeled cRNA yield and specific activity of cyanine3 are shown for each of the three labeling experiments performed. The cRNA yield should be superior to 1.65 μg, and the specific activity superior to 9.0 pmol cyanine3 per μg cRNA. The number of 15k replicates using Quick Amp labeling was 4 for each condition (control or SRSF2 over-expression), and the number of 44k replicates was 6 (*i.e*. 2 for each of the three labeling) for each condition. Click here for file

Additional file 5: Table S4 Comparison of the 15k custom and 44k Agilent^TM^ microarray results. The results are shown for the Agilent^TM^ probes present on both chips: 313 probes were deregulated in SRSF2-over-expressing H358 lung cancer cells in comparison to H358 control cells on the 15k custom chip (≥ 1.5 FC, P-value ≤ 0.05), and 310 had the same type of regulation, considering statistically relevant and not statistically relevant regulations on the 44k chip (same type of regulation = 1; other type of regulation = 0). Click here for file

Additional file 6: Table S5 Supporting evidences. The list of supporting evidences that confirmed the regulation of some probe sets in SRSF2-over-expressing H358 lung cancer cells in comparison to H358 control cells is presented. Click here for file

Additional file 7: Table S6 Quantitative RT-PCR validation. Common regulation events between the 15k custom and 44k Agilent^TM^ microarrays were validated by quantitative RT-PCR in SRSF2-over-expressing H358 lung cancer cells in comparison to H358 control cells. Relative mRNA levels were normalized to that of *GAPDH*. Click here for file

Additional file 8: Figure S2 Design of the custom 15k gene chip. The chip was designed on the backbone of the Agilent^TM^ 15k whole-genome microarray. The majority of the probes correspond to custom oligonucleotides, *i.e.* to both known and predictable sequences of exons, introns and junctions of 16 genes selected for their biological interest in the response to targeted anticancer therapies: *AKT1-3*, *HER1-4*, *HIF1A*, *PIK3CA*, *PIK3R1-2*, *VEGFA-D* and *PIR*. The resolution of the custom microarray was decreased in comparison to the Human Exon 1.0 ST array (Affymetrix^TM^) from 5 million to 12,000 probes, but the number of probes per gene was largely increased, from an average of 45 to an average of 185 probes per gene. The expression of 1,967 distinct genes can also be analyzed thanks to commercial Agilent^TM^ probes. Click here for file

Additional file 9: Figure S3 Western blot analysis of SRSF2 expression. SRSF2 protein level was analyzed in H358/Tet-On/SRSF2 inducible clone by western blotting with the mAb104 monoclonal antibody that recognizes several phosphorylated SR proteins (SRSF2-6). SRSF2 mRNA level was also analyzed by quantitative RT-PCR (data not shown). Relative mRNA level was normalized to that of *GAPDH*. An 8-fold over-expression of SRSF2 mRNA was observed in SRSF2-over-expressing lung cancer cells in comparison to control cells. Click here for file

Additional file 10: Table S7 Primers for validation. The sequences of the primers used for the 15k custom microarray validation are presented. Click here for file
