# Supplementary figures and images for: A dedicated microarray for in-depth analysis of pre-mRNA splicing events: application to the study of genes involved in the response to targeted anticancer therapies
Source: Mol Cancer. 2014 Jan 15;13:9. doi: 10.1186/1476-4598-13-9 (PMC3899606; doi:10.1186/1476-4598-13-9)

## Slide 1
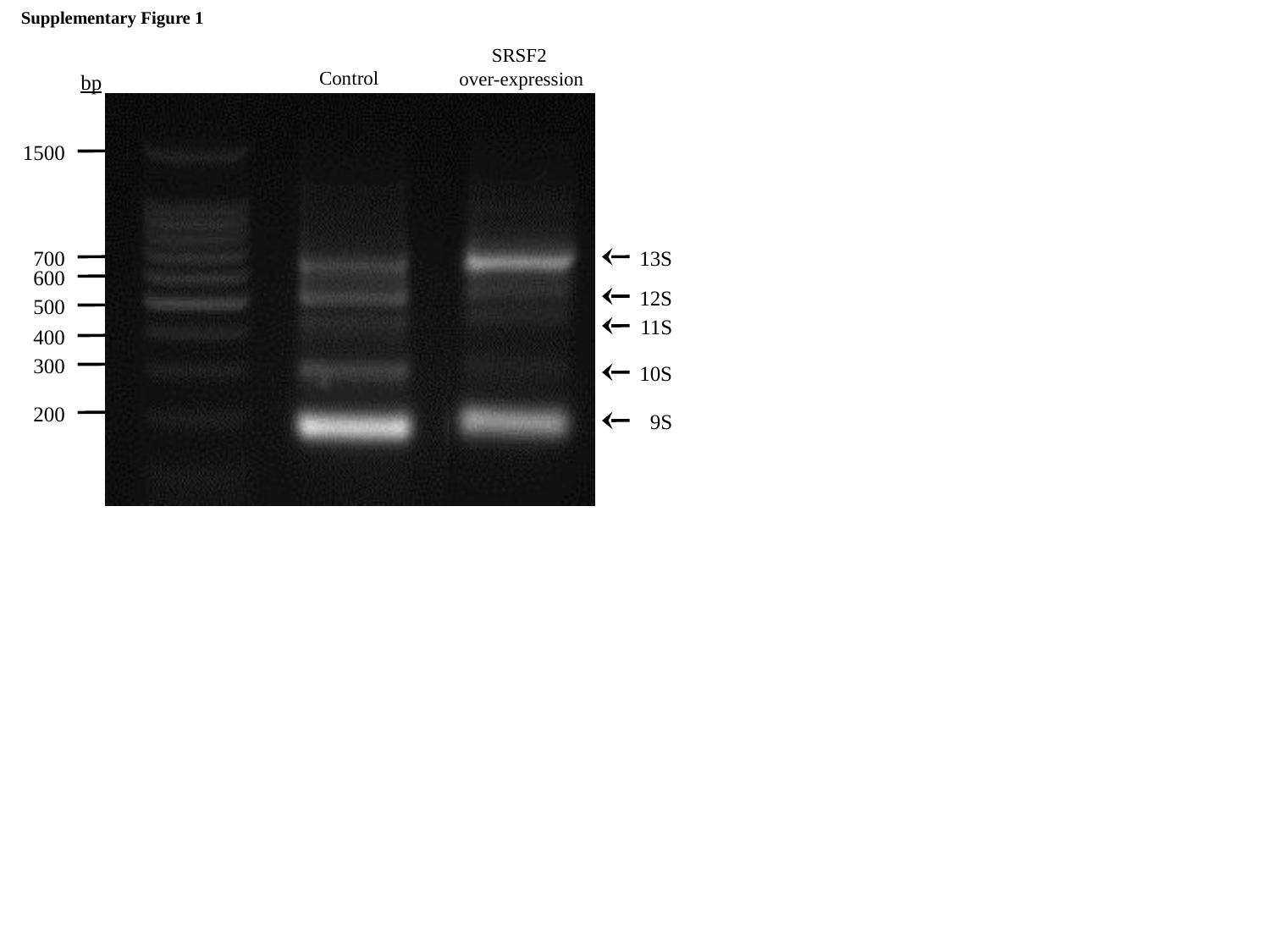

Supplementary Figure 1
SRSF2
 over-expression
Control
bp
1500
700
13S
600
12S
500
11S
400
300
10S
200
9S

Supplement: Additional file 1: Figure S1 — E1A splicing assay in response to SRSF2 over-expression. Following transient cell transfection with a SRSF2 expression plasmid, E1A splice-derived PCR products were electrophoresed through a 2% agarose gel and stained with ethidium bromide. The characteristic PCR products (9S-13S) are shown. [file 1476-4598-13-9-S1.ppt]

## Slide 1
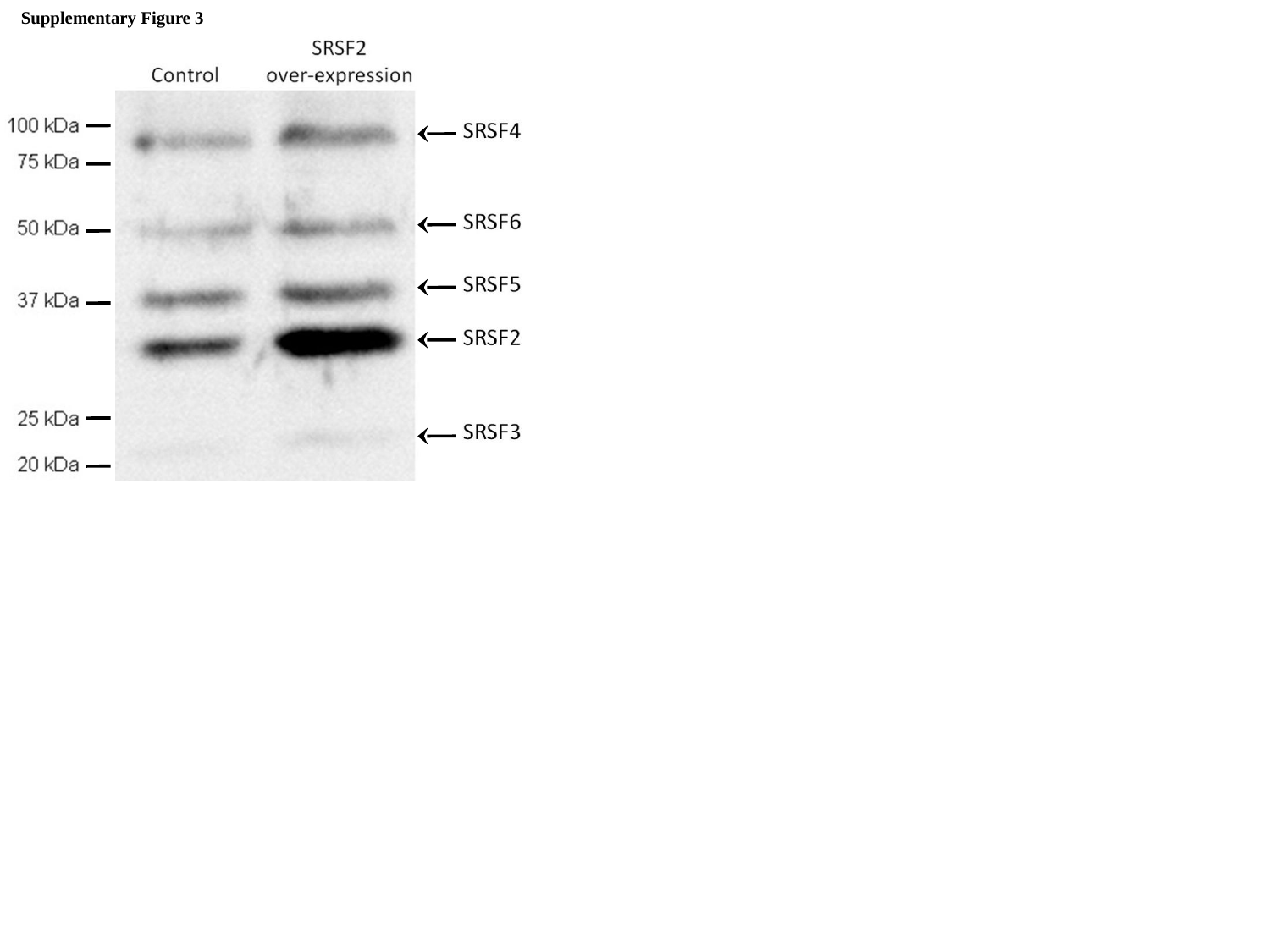

Supplementary Figure 3

Supplement: Additional file 9: Figure S3 — Western blot analysis of SRSF2 expression. SRSF2 protein level was analyzed in H358/Tet-On/SRSF2 inducible clone by western blotting with the mAb104 monoclonal antibody that recognizes several phosphorylated SR proteins (SRSF2-6). SRSF2 mRNA level was also analyzed by quantitative RT-PCR (data not shown). Relative mRNA level was normalized to that of GAPDH. An 8-fold over-expression of SRSF2 mRNA was observed in SRSF2-over-expressing lung cancer cells in comparison to control cells. [file 1476-4598-13-9-S9.ppt]
