# Supplementary material for: A dedicated microarray for in-depth analysis of pre-mRNA splicing events: application to the study of genes involved in the response to targeted anticancer therapies
Source: Mol Cancer. 2014 Jan 15;13:9. doi: 10.1186/1476-4598-13-9 (PMC3899606; doi:10.1186/1476-4598-13-9)
Supplement: Additional file 2: Table S1 — Deregulated genes on the 44k AgilentTM microarray in H358 SRSF2-over-expressing cells. Significantly down- and up-regulated genes in SRSF2-over-expressing H358 lung cancer cells in comparison to H358 control cells are listed (≥ 2.0 FC, P-value ≤ 0.05 by t-test with FDR). [file 1476-4598-13-9-S2.doc]

**Supplementary Table 1.** **Deregulated genes on the 44k Agilent^TM^ microarray in H358 SRSF2-over-expressing cells.** Significantly down- and up-regulated genes in SRSF2-over-expressing H358 lung cancer cells in comparison to H358 control cells are listed (≥ 2.0 FC, P-value ≤ 0.05 by *t*-test with FDR).

| Agilent Probe Name | Gene Symbol | Regulation | Fold-Change | P-Value |
| --- | --- | --- | --- | --- |
| A_24_P324405 | *ANKRD11* | down | 6.77 | 2.93E-06 |
| A_24_P366535 | *LOC100128760* | down | 6.23 | 4.02E-09 |
| A_32_P174374 |  | down | 6.12 | 5.14E-06 |
| A_24_P213503 | *PTPRE* | down | 5.65 | 4.68E-04 |
| A_24_P213494 | *PTPRE* | down | 5.45 | 3.14E-05 |
| A_23_P1331 | *COL13A1* | down | 5.06 | 1.35E-08 |
| A_23_P3632 | *ANKRD11* | down | 4.82 | 8.69E-05 |
| A_24_P621701 |  | down | 4.62 | 4.80E-07 |
| A_23_P133408 | *CSF2* | down | 4.62 | 6.27E-11 |
| A_32_P223327 |  | down | 4.48 | 3.47E-06 |
| A_23_P216779 | *NTRK2* | down | 4.33 | 1.75E-03 |
| A_24_P90005 | *COL13A1* | down | 4.19 | 4.05E-08 |
| A_24_P198598 | *PML* | down | 4.17 | 3.38E-10 |
| A_24_P151 | *KCNAB2* | down | 4.14 | 1.79E-07 |
| A_24_P922877 | *KLC1* | down | 4.09 | 4.92E-09 |
| A_24_P220786 | *RGS12* | down | 4.09 | 3.89E-04 |
| A_24_P924631 |  | down | 3.95 | 5.90E-07 |
| A_23_P398460 | *HK2* | down | 3.93 | 6.47E-05 |
| A_23_P210379 | *ARFGAP1* | down | 3.92 | 3.44E-07 |
| A_24_P243044 | *GSK3B* | down | 3.90 | 5.69E-07 |
| A_24_P696761 | *LEMD1* | down | 3.90 | 8.68E-06 |
| A_32_P169316 |  | down | 3.89 | 1.16E-04 |
| A_23_P218751 | *GNB1L* | down | 3.85 | 2.50E-09 |
| A_32_P87191 |  | down | 3.82 | 2.09E-03 |
| A_23_P250122 | *FAM20C* | down | 3.82 | 2.29E-08 |
| A_23_P366394 | *ZAK* | down | 3.82 | 2.64E-05 |
| A_23_P306148 | *PML* | down | 3.79 | 3.78E-08 |
| A_32_P35047 | *LOC387895* | down | 3.72 | 9.65E-09 |
| A_24_P207139 | *PML* | down | 3.66 | 6.36E-09 |
| A_23_P333735 | *MAD1L1* | down | 3.66 | 1.47E-05 |
| A_32_P192033 | *FLJ31356* | down | 3.65 | 2.15E-07 |
| A_32_P167825 |  | down | 3.64 | 5.18E-05 |
| A_23_P29754 | *BDH1* | down | 3.64 | 3.39E-08 |
| A_23_P31893 | *ST3GAL1* | down | 3.63 | 1.78E-07 |
| A_24_P6030 | *FAM165B* | down | 3.60 | 2.88E-04 |
| A_24_P64100 | *SLC25A37* | down | 3.59 | 8.79E-07 |
| A_24_P8892 | *HDAC4* | down | 3.59 | 2.97E-09 |
| A_23_P59967 | *RP1* | down | 3.58 | 6.94E-05 |
| A_32_P26969 | *ERICH1* | down | 3.54 | 2.64E-08 |
| A_23_P29096 | *PDE9A* | down | 3.54 | 2.74E-08 |
| A_24_P678418 | *FLJ45244* | down | 3.54 | 4.77E-06 |
| A_23_P363426 | *SFRS2B* | down | 3.53 | 4.47E-07 |
| A_32_P213930 |  | down | 3.53 | 4.58E-05 |
| A_23_P209731 | *ARMC9* | down | 3.50 | 2.27E-09 |
| A_24_P272310 | *MUSTN1* | down | 3.49 | 1.02E-05 |
| A_24_P359856 | *HDAC4* | down | 3.48 | 2.87E-08 |
| A_32_P73580 |  | down | 3.48 | 1.52E-07 |
| A_32_P224327 | *LOC730091* | down | 3.45 | 5.22E-07 |
| A_32_P458096 | *TBC1D24* | down | 3.45 | 1.75E-06 |
| A_23_P301521 | *KIAA1462* | down | 3.44 | 1.39E-08 |
| A_24_P133542 | *PML* | down | 3.43 | 2.98E-05 |
| A_23_P425304 | *SUFU* | down | 3.42 | 6.14E-07 |
| A_24_P344087 | *REC8* | down | 3.42 | 3.91E-07 |
| A_24_P354849 |  | down | 3.41 | 2.02E-06 |
| A_23_P20494 | *NDRG1* | down | 3.41 | 6.29E-09 |
| A_32_P82403 | *ANKRD11* | down | 3.41 | 1.35E-08 |
| A_32_P202778 |  | down | 3.41 | 7.70E-06 |
| A_23_P347128 |  | down | 3.38 | 4.94E-09 |
| A_32_P10633 | *TBC1D22A* | down | 3.37 | 1.43E-07 |
| A_23_P207967 | *KIAA0427* | down | 3.37 | 3.20E-08 |
| A_23_P31896 | *ST3GAL1* | down | 3.36 | 1.23E-06 |
| A_23_P165541 |  | down | 3.34 | 1.83E-07 |
| A_24_P58054 | *SLC9A8* | down | 3.33 | 4.53E-09 |
| A_24_P842006 | *C16orf93* | down | 3.33 | 5.94E-09 |
| A_24_P898945 | *C18orf19* | down | 3.32 | 6.16E-04 |
| A_24_P660797 |  | down | 3.31 | 3.82E-03 |
| A_23_P119907 | *ANKZF1* | down | 3.30 | 3.01E-08 |
| A_24_P144936 | *KIAA0319L* | down | 3.28 | 7.02E-03 |
| A_23_P316381 | *ACOX3* | down | 3.28 | 1.45E-07 |
| A_23_P211207 | *ADARB1* | down | 3.27 | 6.03E-08 |
| A_32_P170814 |  | down | 3.27 | 1.78E-05 |
| A_23_P75529 | *PKNOX2* | down | 3.26 | 1.78E-05 |
| A_32_P17635 | *SFRS2B* | down | 3.26 | 8.67E-09 |
| A_24_P921321 | *PTPRJ* | down | 3.25 | 1.85E-06 |
| A_23_P152002 | *BCL2A1* | down | 3.25 | 9.46E-05 |
| A_32_P122715 |  | down | 3.22 | 2.90E-08 |
| A_23_P23947 | *MAP3K8* | down | 3.18 | 1.87E-04 |
| A_32_P84119 |  | down | 3.18 | 1.66E-07 |
| A_32_P138032 | *C1orf61* | down | 3.16 | 6.12E-07 |
| A_24_P85026 | *ZCCHC7* | down | 3.16 | 2.38E-04 |
| A_23_P15751 | *GCGR* | down | 3.15 | 6.69E-05 |
| A_32_P194246 | *CLEC16A* | down | 3.14 | 2.21E-08 |
| A_23_P108751 | *FHL2* | down | 3.14 | 5.09E-07 |
| A_23_P206901 | *NDE1* | down | 3.13 | 4.62E-06 |
| A_23_P135778 | *PPP2R2D* | down | 3.13 | 1.11E-07 |
| A_24_P50908 | *TRIM11* | down | 3.12 | 3.03E-04 |
| A_23_P404965 | *GNL1* | down | 3.10 | 3.34E-06 |
| A_23_P400505 | *C1QTNF9* | down | 3.10 | 8.01E-09 |
| A_24_P317719 | *WHSC1L1* | down | 3.09 | 2.30E-08 |
| A_32_P122268 | *SYT15* | down | 3.09 | 5.55E-08 |
| A_24_P181672 | *B3GNTL1* | down | 3.09 | 7.30E-09 |
| A_24_P566027 |  | down | 3.09 | 3.07E-05 |
| A_23_P215214 | *LMBR1* | down | 3.08 | 5.35E-03 |
| A_23_P332042 | *RECQL5* | down | 3.08 | 3.47E-07 |
| A_32_P175979 |  | down | 3.05 | 1.34E-02 |
| A_24_P76759 | *FARP2* | down | 3.05 | 1.66E-07 |
| A_32_P137299 |  | down | 3.03 | 1.38E-05 |
| A_23_P152505 | *ABAT* | down | 3.03 | 3.76E-07 |
| A_24_P110558 | *C5orf53* | down | 3.02 | 2.89E-06 |
| A_32_P75772 |  | down | 3.02 | 1.28E-07 |
| A_23_P315836 | *BAIAP2* | down | 3.01 | 8.03E-08 |
| A_32_P161455 |  | down | 3.01 | 1.14E-03 |
| A_24_P252130 | *PPARD* | down | 3.00 | 1.02E-08 |
| A_23_P209735 | *ARMC9* | down | 3.00 | 6.17E-11 |
| A_32_P59116 | *GOLGA8F* | down | 3.00 | 1.19E-03 |
| A_23_P5831 | *HPCAL1* | down | 2.99 | 2.97E-08 |
| A_32_P144018 |  | down | 2.98 | 1.79E-04 |
| A_32_P172803 | *MAP9* | down | 2.98 | 2.10E-03 |
| A_23_P83599 | *PRKAR1B* | down | 2.98 | 2.03E-06 |
| A_32_P196287 |  | down | 2.98 | 1.47E-08 |
| A_23_P70448 | *HIST1H1A* | down | 2.98 | 2.78E-04 |
| A_32_P165993 |  | down | 2.97 | 2.31E-03 |
| A_24_P3016 |  | down | 2.97 | 4.12E-06 |
| A_32_P197720 |  | down | 2.97 | 3.94E-05 |
| A_23_P126159 | *HPCA* | down | 2.96 | 4.64E-07 |
| A_23_P123193 | *ACTR3B* | down | 2.96 | 1.27E-08 |
| A_24_P356916 | *SLC13A3* | down | 2.96 | 1.53E-07 |
| A_24_P595567 |  | down | 2.96 | 8.55E-04 |
| A_23_P428842 | *TMEM44* | down | 2.95 | 1.60E-06 |
| A_24_P933138 |  | down | 2.95 | 6.70E-06 |
| A_32_P234661 |  | down | 2.95 | 2.83E-05 |
| A_23_P142304 | *MKNK2* | down | 2.94 | 3.95E-08 |
| A_32_P154473 | *KIF5C* | down | 2.94 | 1.78E-05 |
| A_23_P210048 | *HDAC4* | down | 2.94 | 1.69E-06 |
| A_23_P354547 |  | down | 2.94 | 4.95E-05 |
| A_32_P37247 | *LOC642826* | down | 2.93 | 4.66E-04 |
| A_23_P163639 | *ANKRD11* | down | 2.93 | 7.91E-07 |
| A_23_P161507 | *MTL5* | down | 2.92 | 1.29E-08 |
| A_24_P678741 | *KIAA1671* | down | 2.92 | 5.63E-04 |
| A_23_P384816 | *SLC45A4* | down | 2.92 | 1.30E-06 |
| A_24_P360722 | *DIP2C* | down | 2.91 | 6.36E-05 |
| A_23_P162378 | *CCDC41* | down | 2.91 | 1.04E-07 |
| A_23_P155057 | *CYTH4* | down | 2.90 | 2.78E-06 |
| A_24_P119283 | *ZC3H7A* | down | 2.89 | 4.35E-05 |
| A_23_P393856 | *ST3GAL4* | down | 2.89 | 1.04E-07 |
| A_32_P161633 |  | down | 2.89 | 1.62E-04 |
| A_23_P160787 | *PEX14* | down | 2.89 | 2.13E-08 |
| A_23_P329870 | *RHBDF2* | down | 2.89 | 1.75E-08 |
| A_24_P413126 | *PMEPA1* | down | 2.88 | 6.49E-09 |
| A_24_P938465 |  | down | 2.88 | 2.90E-03 |
| A_24_P227993 | *UBE2I* | down | 2.88 | 1.19E-06 |
| A_23_P214066 | *ARHGAP26* | down | 2.88 | 1.44E-08 |
| A_23_P51926 | *PTAFR* | down | 2.88 | 3.60E-08 |
| A_23_P200267 | *PCNXL2* | down | 2.87 | 2.66E-06 |
| A_23_P145889 | *CDK14* | down | 2.87 | 3.92E-05 |
| A_32_P80697 |  | down | 2.87 | 9.87E-05 |
| A_24_P926666 |  | down | 2.86 | 9.36E-10 |
| A_24_P368544 | *SLC25A26* | down | 2.86 | 7.86E-04 |
| A_32_P84333 |  | down | 2.85 | 4.07E-08 |
| A_23_P302914 | *ZFYVE28* | down | 2.85 | 2.11E-05 |
| A_32_P91107 |  | down | 2.85 | 1.58E-09 |
| A_32_P168388 |  | down | 2.84 | 9.29E-03 |
| A_24_P479364 |  | down | 2.84 | 1.29E-03 |
| A_23_P97423 | *UBE2Q1* | down | 2.83 | 2.24E-09 |
| A_24_P239364 | *TMEFF2* | down | 2.83 | 3.89E-06 |
| A_24_P919683 |  | down | 2.82 | 4.70E-06 |
| A_32_P205913 |  | down | 2.82 | 1.43E-03 |
| A_23_P140725 | *IFT140* | down | 2.82 | 1.89E-08 |
| A_32_P55161 | *AGAP1* | down | 2.81 | 7.97E-05 |
| A_24_P117964 | *BOD1* | down | 2.81 | 6.79E-04 |
| A_23_P400217 | *MUM1* | down | 2.80 | 5.10E-08 |
| A_32_P115947 | *GTPBP4* | down | 2.80 | 3.98E-08 |
| A_32_P128097 |  | down | 2.80 | 2.73E-06 |
| A_32_P37584 |  | down | 2.80 | 1.00E-03 |
| A_32_P542928 | *ZNF789* | down | 2.80 | 6.51E-06 |
| A_23_P9836 | *ETV5* | down | 2.79 | 1.95E-04 |
| A_32_P48526 |  | down | 2.79 | 1.13E-08 |
| A_23_P42116 | *PPT2* | down | 2.79 | 1.30E-09 |
| A_23_P218997 | *PDCD6* | down | 2.78 | 4.30E-08 |
| A_24_P262127 | *RRAD* | down | 2.77 | 1.37E-07 |
| A_23_P93348 | *LTB* | down | 2.77 | 3.32E-06 |
| A_23_P23584 | *CTNNBIP1* | down | 2.77 | 1.23E-08 |
| A_23_P88849 | *RRAD* | down | 2.77 | 2.32E-06 |
| A_24_P361457 | *FLJ35220* | down | 2.77 | 1.25E-03 |
| A_24_P323941 | *C20orf106* | down | 2.76 | 2.55E-07 |
| A_32_P9816 |  | down | 2.76 | 4.21E-05 |
| A_32_P127583 |  | down | 2.76 | 9.75E-03 |
| A_24_P213763 |  | down | 2.76 | 2.79E-04 |
| A_24_P320645 | *SUN1* | down | 2.76 | 1.14E-07 |
| A_23_P77776 | *SFRS2* | down | 2.76 | 1.31E-08 |
| A_23_P206310 | *KIAA0513* | down | 2.76 | 1.71E-06 |
| A_24_P278299 | *ASB13* | down | 2.76 | 8.25E-09 |
| A_24_P364087 | *SERGEF* | down | 2.75 | 4.22E-10 |
| A_24_P364296 | *STX2* | down | 2.75 | 7.85E-06 |
| A_24_P307695 | *LRRCC1* | down | 2.75 | 3.06E-05 |
| A_24_P862122 |  | down | 2.74 | 3.79E-05 |
| A_23_P143374 | *NINL* | down | 2.74 | 1.55E-09 |
| A_23_P204782 | *MDM1* | down | 2.73 | 2.64E-02 |
| A_23_P77430 | *PRMT7* | down | 2.73 | 2.15E-08 |
| A_23_P160154 | *GALE* | down | 2.73 | 5.15E-07 |
| A_24_P792130 | *LOC100289574* | down | 2.73 | 1.60E-04 |
| A_24_P337000 | *TTC7A* | down | 2.73 | 5.03E-07 |
| A_32_P155512 |  | down | 2.73 | 1.50E-02 |
| A_23_P157838 |  | down | 2.72 | 2.31E-06 |
| A_23_P42530 | *DST* | down | 2.72 | 1.39E-03 |
| A_24_P354451 | *ACOX3* | down | 2.72 | 4.76E-05 |
| A_24_P938135 |  | down | 2.72 | 1.18E-08 |
| A_24_P830690 | *PDPK1* | down | 2.71 | 3.53E-05 |
| A_32_P124493 | *LOC642826* | down | 2.71 | 4.57E-05 |
| A_23_P77437 | *PRMT7* | down | 2.71 | 7.54E-08 |
| A_32_P8732 |  | down | 2.71 | 2.01E-06 |
| A_23_P422212 | *SLC35F3* | down | 2.70 | 2.21E-09 |
| A_23_P22460 | *XIAP* | down | 2.70 | 4.36E-09 |
| A_24_P102821 | *PTAFR* | down | 2.69 | 2.22E-07 |
| A_32_P177477 |  | down | 2.69 | 1.22E-06 |
| A_32_P35668 |  | down | 2.69 | 9.88E-06 |
| A_24_P272761 | *DENND1A* | down | 2.68 | 1.34E-06 |
| A_23_P119794 |  | down | 2.68 | 5.18E-07 |
| A_23_P129334 | *CLCN7* | down | 2.68 | 2.25E-05 |
| A_23_P423695 | *MXD4* | down | 2.67 | 6.44E-07 |
| A_32_P142088 | *MPZL1* | down | 2.67 | 5.93E-04 |
| A_23_P35456 | *SH3PXD2A* | down | 2.67 | 1.17E-08 |
| A_23_P165180 | *RFXANK* | down | 2.66 | 3.49E-07 |
| A_23_P214789 | *SNX9* | down | 2.66 | 7.63E-10 |
| A_32_P33802 |  | down | 2.66 | 3.30E-08 |
| A_23_P20303 | *DPYS* | down | 2.65 | 2.33E-03 |
| A_32_P233735 | *FAM40B* | down | 2.65 | 1.69E-04 |
| A_24_P927936 |  | down | 2.65 | 3.88E-04 |
| A_24_P346368 | *C7orf42* | down | 2.65 | 7.33E-09 |
| A_23_P34325 | *LRP8* | down | 2.65 | 7.35E-06 |
| A_32_P153361 |  | down | 2.65 | 1.31E-08 |
| A_32_P353798 | *PTDSS2* | down | 2.65 | 2.53E-10 |
| A_23_P350555 | *TCP10L* | down | 2.65 | 1.07E-06 |
| A_32_P205053 | *UBXN10* | down | 2.64 | 7.28E-04 |
| A_23_P35650 | *RBM17* | down | 2.64 | 2.99E-07 |
| A_23_P305100 | *KIAA1919* | down | 2.63 | 7.34E-07 |
| A_23_P304991 | *HLCS* | down | 2.63 | 1.03E-07 |
| A_23_P123622 | *NPR2* | down | 2.63 | 1.31E-06 |
| A_32_P9597 | *ST6GALNAC2* | down | 2.63 | 1.57E-05 |
| A_23_P103942 | *DNAJC11* | down | 2.62 | 2.78E-06 |
| A_32_P133999 | *CALN1* | down | 2.62 | 3.07E-05 |
| A_32_P23854 | *DENND1A* | down | 2.62 | 1.17E-08 |
| A_24_P219053 | *KIAA1704* | down | 2.61 | 1.33E-04 |
| A_24_P928969 | *PTPN3* | down | 2.61 | 4.41E-07 |
| A_23_P111531 | *GLI3* | down | 2.61 | 2.41E-08 |
| A_24_P289178 | *C16orf74* | down | 2.61 | 6.02E-06 |
| A_24_P396327 | *TYW3* | down | 2.61 | 1.24E-03 |
| A_24_P911191 |  | down | 2.61 | 4.69E-05 |
| A_23_P135239 | *TLE1* | down | 2.61 | 1.49E-05 |
| A_32_P34881 |  | down | 2.60 | 6.55E-07 |
| A_24_P150486 | *SPTLC2* | down | 2.60 | 2.21E-03 |
| A_32_P216122 |  | down | 2.60 | 4.41E-06 |
| A_23_P152125 | *MVD* | down | 2.60 | 6.80E-07 |
| A_23_P347131 | *TTC15* | down | 2.60 | 1.61E-09 |
| A_23_P301304 | *FGFR1* | down | 2.60 | 1.13E-05 |
| A_23_P212284 | *WDR51A* | down | 2.60 | 5.10E-08 |
| A_23_P69652 | *GPR78* | down | 2.59 | 1.32E-03 |
| A_32_P22883 | *C21orf70* | down | 2.59 | 3.45E-07 |
| A_23_P256835 | *TTC15* | down | 2.59 | 3.55E-07 |
| A_23_P60106 |  | down | 2.59 | 1.87E-03 |
| A_23_P345678 | *FANCD2* | down | 2.59 | 1.77E-04 |
| A_24_P811704 | *PPFIBP1* | down | 2.59 | 3.86E-04 |
| A_32_P113736 |  | down | 2.59 | 4.54E-06 |
| A_23_P213959 | *PPARGC1B* | down | 2.58 | 2.60E-05 |
| A_23_P406122 | *C20orf194* | down | 2.58 | 2.51E-02 |
| A_24_P28739 | *CCDC88A* | down | 2.58 | 7.96E-04 |
| A_24_P206305 | *MICALL2* | down | 2.58 | 3.33E-07 |
| A_32_P82869 |  | down | 2.58 | 2.99E-04 |
| A_32_P25243 |  | down | 2.57 | 1.44E-07 |
| A_23_P129801 | *RAB40B* | down | 2.57 | 2.66E-06 |
| A_23_P116602 | *USP35* | down | 2.57 | 1.81E-09 |
| A_32_P152348 | *HIST1H2BD* | down | 2.57 | 4.06E-04 |
| A_24_P743802 | *ZNF618* | down | 2.57 | 1.02E-04 |
| A_23_P23616 | *PLEKHN1* | down | 2.56 | 2.30E-03 |
| A_24_P74070 | *PARD6G* | down | 2.56 | 2.72E-06 |
| A_32_P119165 |  | down | 2.56 | 3.21E-07 |
| A_23_P431268 | *PLEKHA6* | down | 2.56 | 2.90E-07 |
| A_23_P98248 | *TRPT1* | down | 2.56 | 2.41E-09 |
| A_23_P147698 | *CALN1* | down | 2.56 | 1.07E-05 |
| A_24_P940909 | *TTC7A* | down | 2.55 | 1.36E-03 |
| A_23_P308119 | *TPCN1* | down | 2.55 | 9.06E-07 |
| A_24_P190804 | *AP1S2* | down | 2.55 | 5.36E-07 |
| A_23_P60324 | *UBAC1* | down | 2.55 | 7.27E-10 |
| A_23_P366328 | *VPS37A* | down | 2.55 | 1.52E-07 |
| A_23_P3295 | *FAM81A* | down | 2.54 | 3.01E-06 |
| A_32_P330691 |  | down | 2.54 | 1.40E-03 |
| A_23_P417383 | *ASPRV1* | down | 2.54 | 1.90E-06 |
| A_24_P920135 |  | down | 2.54 | 7.15E-03 |
| A_24_P338603 | *SKI* | down | 2.54 | 2.62E-03 |
| A_32_P82189 | *FAM161A* | down | 2.53 | 2.28E-03 |
| A_23_P424878 | *KIAA1688* | down | 2.53 | 1.16E-07 |
| A_32_P31355 |  | down | 2.53 | 3.19E-05 |
| A_23_P200222 | *LRP8* | down | 2.53 | 1.69E-07 |
| A_23_P66219 | *PDPK1* | down | 2.53 | 1.11E-05 |
| A_32_P85273 | *GOLGA8A* | down | 2.53 | 8.24E-05 |
| A_24_P538590 |  | down | 2.53 | 2.81E-08 |
| A_24_P346210 | *BTN2A2* | down | 2.52 | 6.57E-06 |
| A_24_P588897 | *SLCO3A1* | down | 2.52 | 1.43E-05 |
| A_24_P667838 |  | down | 2.52 | 5.11E-04 |
| A_23_P4353 | *WSB1* | down | 2.52 | 4.76E-05 |
| A_24_P76725 | *DCAF8* | down | 2.52 | 2.82E-02 |
| A_23_P307536 | *C1orf113* | down | 2.51 | 2.87E-08 |
| A_23_P381945 | *KRT7* | down | 2.51 | 1.23E-08 |
| A_23_P38941 | *EPS15L1* | down | 2.51 | 5.06E-03 |
| A_23_P35645 | *RBM17* | down | 2.51 | 2.13E-09 |
| A_24_P29733 | *CDK14* | down | 2.51 | 5.13E-06 |
| A_23_P75260 | *RASSF4* | down | 2.51 | 8.69E-06 |
| A_23_P109593 | *TBC1D22A* | down | 2.51 | 3.60E-06 |
| A_23_P202750 | *C11orf54* | down | 2.50 | 4.41E-07 |
| A_24_P284324 | *TMEM104* | down | 2.50 | 4.94E-09 |
| A_24_P215765 | *ATP10A* | down | 2.50 | 6.50E-05 |
| A_23_P105862 | *FRY* | down | 2.50 | 1.43E-03 |
| A_32_P25972 |  | down | 2.50 | 4.30E-04 |
| A_24_P298939 | *EP400* | down | 2.49 | 5.29E-03 |
| A_32_P13151 | *LOC284232* | down | 2.48 | 2.62E-03 |
| A_24_P84419 | *VAV2* | down | 2.48 | 1.03E-06 |
| A_23_P12884 | *GRK5* | down | 2.48 | 6.60E-08 |
| A_23_P256205 | *ABLIM3* | down | 2.48 | 2.54E-08 |
| A_32_P151747 |  | down | 2.48 | 1.68E-05 |
| A_23_P258124 | *ZNF346* | down | 2.48 | 5.92E-06 |
| A_23_P435183 | *LRRFIP1* | down | 2.48 | 6.59E-08 |
| A_32_P192842 | *LOC100288985* | down | 2.48 | 1.82E-05 |
| A_23_P252764 | *SMARCA2* | down | 2.48 | 6.24E-04 |
| A_23_P256375 | *STX4* | down | 2.47 | 6.13E-05 |
| A_23_P8311 | *TTRAP* | down | 2.47 | 1.01E-07 |
| A_32_P57810 | *RNF157* | down | 2.46 | 8.00E-05 |
| A_23_P390116 | *SPATA13* | down | 2.46 | 1.31E-02 |
| A_24_P194881 | *SHANK3* | down | 2.46 | 1.07E-06 |
| A_32_P115277 |  | down | 2.46 | 5.75E-05 |
| A_23_P68730 | *PDXK* | down | 2.46 | 1.75E-08 |
| A_24_P46808 |  | down | 2.45 | 3.42E-09 |
| A_23_P61646 | *STX4* | down | 2.45 | 3.68E-05 |
| A_32_P182609 |  | down | 2.45 | 6.73E-04 |
| A_24_P65910 | *UQCC* | down | 2.45 | 1.17E-08 |
| A_24_P916496 | *PRKCA* | down | 2.45 | 1.03E-06 |
| A_24_P693448 | *ZNF552* | down | 2.45 | 4.34E-02 |
| A_23_P58604 | *C5orf45* | down | 2.45 | 9.43E-11 |
| A_23_P253738 | *CLN8* | down | 2.45 | 9.29E-04 |
| A_24_P105913 | *AZI2* | down | 2.44 | 1.17E-04 |
| A_24_P57700 | *ZHX3* | down | 2.44 | 5.11E-07 |
| A_32_P220762 | *OSBPL6* | down | 2.44 | 1.06E-05 |
| A_23_P398491 | *MRPL42P5* | down | 2.44 | 3.45E-04 |
| A_23_P6023 | *RAE1* | down | 2.44 | 1.74E-06 |
| A_23_P12572 | *CASP7* | down | 2.44 | 9.61E-07 |
| A_24_P873764 | *BCR* | down | 2.43 | 2.96E-08 |
| A_24_P193482 | *ADAL* | down | 2.43 | 6.90E-07 |
| A_23_P352435 | *RGS12* | down | 2.43 | 2.64E-07 |
| A_32_P161327 |  | down | 2.42 | 8.67E-03 |
| A_23_P257497 | *LOC100130967* | down | 2.42 | 9.28E-08 |
| A_23_P166779 | *LOH3CR2A* | down | 2.42 | 5.20E-06 |
| A_24_P555791 | *HERC2* | down | 2.42 | 2.82E-05 |
| A_24_P913227 | *CDC23* | down | 2.42 | 3.12E-06 |
| A_23_P257743 | *SHB* | down | 2.42 | 2.80E-07 |
| A_23_P217339 | *PRKX* | down | 2.42 | 1.93E-09 |
| A_23_P54963 | *MRPL38* | down | 2.42 | 2.29E-08 |
| A_23_P66988 | *ONECUT2* | down | 2.42 | 4.35E-04 |
| A_23_P382043 | *NT5DC1* | down | 2.42 | 1.42E-08 |
| A_24_P306561 | *TCF25* | down | 2.42 | 5.49E-04 |
| A_24_P146575 | *SHB* | down | 2.42 | 4.12E-08 |
| A_32_P226356 |  | down | 2.42 | 6.52E-05 |
| A_32_P171856 |  | down | 2.41 | 1.93E-04 |
| A_23_P117480 |  | down | 2.41 | 4.81E-04 |
| A_32_P877 |  | down | 2.41 | 9.95E-05 |
| A_23_P300150 | *NFATC1* | down | 2.41 | 4.04E-06 |
| A_23_P110031 | *ZNF589* | down | 2.41 | 1.85E-08 |
| A_24_P313397 |  | down | 2.41 | 3.78E-08 |
| A_23_P503182 | *ABR* | down | 2.41 | 3.79E-09 |
| A_24_P135319 | *CGNL1* | down | 2.41 | 4.28E-02 |
| A_23_P57089 | *PMEPA1* | down | 2.41 | 4.07E-07 |
| A_23_P63829 | *HSPA14* | down | 2.41 | 8.75E-10 |
| A_24_P139943 | *HS1BP3* | down | 2.41 | 6.66E-06 |
| A_24_P364025 | *UBE2D1* | down | 2.41 | 9.45E-04 |
| A_23_P61202 | *C21orf70* | down | 2.40 | 2.29E-06 |
| A_32_P161661 | *TSNARE1* | down | 2.40 | 9.90E-07 |
| A_23_P340188 | *FAM122C* | down | 2.40 | 9.41E-05 |
| A_32_P181297 | *ST7OT1* | down | 2.40 | 3.14E-05 |
| A_32_P92117 |  | down | 2.40 | 1.53E-04 |
| A_23_P160638 | *BGLAP* | down | 2.39 | 4.12E-09 |
| A_24_P307759 | *SYNE2* | down | 2.39 | 5.85E-03 |
| A_23_P89310 | *EPN2* | down | 2.39 | 4.84E-07 |
| A_23_P157247 | *TMEM184A* | down | 2.39 | 1.91E-05 |
| A_32_P108738 | *CCDC149* | down | 2.39 | 1.72E-06 |
| A_23_P140405 | *FOXN3* | down | 2.39 | 3.33E-09 |
| A_23_P98350 | *BIRC3* | down | 2.39 | 5.83E-09 |
| A_32_P124125 |  | down | 2.38 | 2.03E-03 |
| A_23_P50455 | *POLD1* | down | 2.38 | 1.64E-07 |
| A_23_P163801 | *KLHDC4* | down | 2.38 | 9.03E-06 |
| A_23_P500741 | *CBFA2T3* | down | 2.38 | 1.31E-06 |
| A_32_P799227 | *FAM22A* | down | 2.38 | 2.08E-03 |
| A_23_P9823 | *MLXIP* | down | 2.38 | 1.77E-05 |
| A_32_P93852 | *BOD1* | down | 2.38 | 1.13E-07 |
| A_32_P330000 | *LOC400931* | down | 2.38 | 6.09E-06 |
| A_23_P163148 | *VIPAR* | down | 2.37 | 7.22E-09 |
| A_23_P62967 | *DISC1* | down | 2.37 | 1.93E-08 |
| A_24_P219378 | *CASKIN1* | down | 2.37 | 4.13E-04 |
| A_32_P43812 | *DCUN1D4* | down | 2.37 | 4.78E-07 |
| A_32_P35452 |  | down | 2.37 | 8.08E-03 |
| A_24_P400172 | *hCG_1817306* | down | 2.37 | 1.29E-06 |
| A_23_P390596 | *PSKH1* | down | 2.37 | 3.40E-07 |
| A_24_P678743 | *KIAA1671* | down | 2.36 | 3.48E-03 |
| A_23_P48771 | *C14orf159* | down | 2.36 | 7.71E-09 |
| A_24_P662177 |  | down | 2.36 | 2.31E-06 |
| A_24_P247978 | *ZNF589* | down | 2.36 | 2.51E-03 |
| A_23_P163697 | *SYT17* | down | 2.36 | 3.17E-06 |
| A_24_P337012 | *SP140L* | down | 2.36 | 5.15E-03 |
| A_23_P330788 | *IQSEC2* | down | 2.36 | 1.66E-05 |
| A_32_P132276 |  | down | 2.36 | 3.40E-07 |
| A_23_P106727 | *RAB11FIP3* | down | 2.36 | 1.39E-06 |
| A_23_P427083 | *CDRT4* | down | 2.36 | 1.80E-06 |
| A_24_P280558 | *FLJ40330* | down | 2.35 | 2.60E-05 |
| A_23_P59637 | *DOCK4* | down | 2.35 | 1.28E-04 |
| A_24_P524452 |  | down | 2.35 | 4.52E-04 |
| A_32_P89976 | *POLR1E* | down | 2.35 | 4.86E-08 |
| A_23_P431330 | *CRIPAK* | down | 2.35 | 5.54E-09 |
| A_23_P139207 | *SERGEF* | down | 2.35 | 1.07E-06 |
| A_23_P422083 | *TMEM55A* | down | 2.35 | 3.33E-05 |
| A_24_P526177 | *TDG* | down | 2.35 | 8.98E-05 |
| A_23_P163099 | *POLE2* | down | 2.35 | 1.18E-08 |
| A_23_P355439 | *HIST1H2AA* | down | 2.34 | 2.23E-05 |
| A_23_P44105 | *LAT* | down | 2.34 | 6.40E-09 |
| A_32_P56713 | *BCR* | down | 2.34 | 4.12E-09 |
| A_23_P24192 | *RRP12* | down | 2.34 | 3.37E-06 |
| A_32_P54616 | *KIAA1407* | down | 2.34 | 8.82E-03 |
| A_23_P29303 | *RRP7A* | down | 2.34 | 2.25E-06 |
| A_24_P213370 |  | down | 2.34 | 4.03E-05 |
| A_23_P55099 | *PRKCA* | down | 2.34 | 3.37E-06 |
| A_23_P57760 | *ACPL2* | down | 2.33 | 2.01E-09 |
| A_23_P259328 | *PEX3* | down | 2.33 | 5.64E-06 |
| A_24_P497226 | *RPS6KB1* | down | 2.33 | 4.99E-07 |
| A_24_P234732 | *MXD4* | down | 2.33 | 6.75E-05 |
| A_23_P216282 | *ARHGEF10* | down | 2.33 | 1.69E-07 |
| A_23_P105753 | *PWP1* | down | 2.33 | 3.12E-06 |
| A_24_P212096 | *NAV1* | down | 2.33 | 1.08E-03 |
| A_24_P201404 | *C11orf54* | down | 2.33 | 8.20E-09 |
| A_32_P154223 |  | down | 2.32 | 4.65E-06 |
| A_23_P88309 | *SLC38A6* | down | 2.32 | 2.11E-08 |
| A_23_P401547 | *PVRL3* | down | 2.32 | 8.61E-05 |
| A_24_P450172 |  | down | 2.32 | 8.02E-04 |
| A_32_P128391 |  | down | 2.32 | 1.81E-07 |
| A_24_P354300 | *WDR51A* | down | 2.32 | 3.41E-08 |
| A_24_P159648 | *BAIAP2* | down | 2.32 | 6.36E-09 |
| A_24_P636974 | *LOC100289383* | down | 2.32 | 2.98E-06 |
| A_23_P15202 | *DHODH* | down | 2.31 | 1.72E-07 |
| A_23_P61623 | *SLCO3A1* | down | 2.31 | 6.31E-07 |
| A_32_P219963 | *LOC100133091* | down | 2.31 | 4.31E-07 |
| A_23_P50456 | *POLD1* | down | 2.31 | 2.81E-04 |
| A_32_P142334 |  | down | 2.31 | 9.55E-05 |
| A_23_P91783 | *PRR5-ARHGAP8* | down | 2.31 | 4.56E-08 |
| A_23_P154539 | *PRPF6* | down | 2.31 | 9.16E-05 |
| A_23_P58960 | *AGPAT4* | down | 2.31 | 1.16E-05 |
| A_24_P337746 | *RABGEF1* | down | 2.31 | 3.21E-06 |
| A_23_P61230 | *HGS* | down | 2.31 | 2.34E-06 |
| A_32_P120604 |  | down | 2.30 | 2.03E-06 |
| A_32_P2213 |  | down | 2.30 | 1.09E-06 |
| A_32_P35486 |  | down | 2.30 | 1.38E-05 |
| A_24_P642758 |  | down | 2.30 | 1.51E-07 |
| A_32_P200165 | *ELAVL1* | down | 2.30 | 1.72E-06 |
| A_24_P213134 |  | down | 2.30 | 1.94E-07 |
| A_23_P364537 | *DDX51* | down | 2.30 | 3.18E-04 |
| A_24_P111134 | *POMT2* | down | 2.30 | 3.91E-06 |
| A_32_P57717 |  | down | 2.30 | 1.03E-05 |
| A_24_P915535 |  | down | 2.30 | 1.33E-03 |
| A_24_P160088 | *PDXK* | down | 2.30 | 1.22E-06 |
| A_23_P1322 | *AKR1E2* | down | 2.29 | 1.34E-06 |
| A_24_P392480 | *MYPOP* | down | 2.29 | 1.06E-04 |
| A_32_P96692 | *POLH* | down | 2.29 | 2.43E-07 |
| A_23_P24843 | *MICAL2* | down | 2.29 | 1.18E-07 |
| A_23_P124855 | *ZCCHC7* | down | 2.29 | 3.54E-06 |
| A_23_P89871 | *ZNF415* | down | 2.29 | 2.31E-08 |
| A_24_P910580 | *GOLGA8A* | down | 2.28 | 9.78E-03 |
| A_24_P945408 | *ARMC9* | down | 2.28 | 1.10E-04 |
| A_23_P64499 | *MOB2* | down | 2.28 | 3.28E-08 |
| A_32_P154361 |  | down | 2.28 | 1.01E-06 |
| A_23_P171385 | *PHF6* | down | 2.28 | 1.32E-06 |
| A_23_P335452 | *ZCCHC24* | down | 2.28 | 7.55E-08 |
| A_23_P123308 | *TEX15* | down | 2.27 | 3.42E-03 |
| A_23_P156445 | *DDX43* | down | 2.27 | 4.78E-07 |
| A_23_P377819 | *SFRS5* | down | 2.27 | 2.01E-09 |
| A_23_P84821 | *MRPL1* | down | 2.27 | 1.06E-07 |
| A_23_P108135 | *AP3D1* | down | 2.27 | 3.52E-03 |
| A_32_P140079 |  | down | 2.27 | 2.09E-03 |
| A_24_P23258 | *GRAMD4* | down | 2.27 | 2.29E-07 |
| A_23_P88489 | *CATSPER2* | down | 2.26 | 3.20E-07 |
| A_23_P154457 | *NOL10* | down | 2.26 | 2.76E-06 |
| A_24_P312164 | *SLC25A40* | down | 2.26 | 6.94E-06 |
| A_23_P130488 | *ERCC2* | down | 2.26 | 1.80E-05 |
| A_23_P97283 | *PAQR6* | down | 2.26 | 3.04E-08 |
| A_24_P187131 | *FRYL* | down | 2.26 | 5.59E-06 |
| A_23_P74115 | *RAD54L* | down | 2.26 | 2.92E-08 |
| A_23_P371239 | *CMIP* | down | 2.26 | 7.56E-07 |
| A_24_P28165 | *ARHGAP26* | down | 2.26 | 1.74E-07 |
| A_24_P79153 | *SCAMP4* | down | 2.26 | 1.47E-07 |
| A_23_P395374 | *HIST1H4D* | down | 2.26 | 1.09E-04 |
| A_23_P54956 | *MRPL38* | down | 2.25 | 9.86E-09 |
| A_24_P886515 |  | down | 2.25 | 4.04E-07 |
| A_24_P325992 | *LIFR* | down | 2.25 | 1.48E-02 |
| A_24_P205627 | *TXLNA* | down | 2.25 | 8.51E-07 |
| A_24_P350124 | *RNF213* | down | 2.25 | 4.06E-06 |
| A_24_P942354 | *PITPNA* | down | 2.25 | 1.84E-06 |
| A_23_P10701 | *APBB2* | down | 2.25 | 2.56E-07 |
| A_24_P236091 | *ENO2* | down | 2.25 | 3.58E-05 |
| A_23_P130856 | *CCDC123* | down | 2.25 | 3.09E-07 |
| A_23_P208293 | *PVRL2* | down | 2.25 | 3.46E-08 |
| A_23_P367816 | *SNX29* | down | 2.25 | 9.35E-07 |
| A_23_P90359 | *NRTN* | down | 2.25 | 1.54E-04 |
| A_24_P943613 | *TBC1D1* | down | 2.25 | 4.96E-06 |
| A_24_P579826 |  | down | 2.24 | 5.66E-06 |
| A_24_P236008 | *SCYL2* | down | 2.24 | 1.99E-06 |
| A_23_P376372 | *MARVELD3* | down | 2.24 | 1.62E-04 |
| A_23_P16483 | *STK11* | down | 2.24 | 3.52E-06 |
| A_23_P52610 | *DDB2* | down | 2.24 | 9.42E-07 |
| A_24_P218587 | *MED17* | down | 2.24 | 1.23E-07 |
| A_24_P406006 | *LPCAT1* | down | 2.24 | 1.76E-07 |
| A_23_P382654 | *ASCC3* | down | 2.24 | 8.42E-05 |
| A_23_P423482 | *FAM81A* | down | 2.24 | 1.37E-03 |
| A_32_P98979 |  | down | 2.24 | 2.68E-02 |
| A_24_P38387 | *NDRG1* | down | 2.24 | 3.36E-02 |
| A_24_P405430 | *TIA1* | down | 2.24 | 1.85E-05 |
| A_32_P215078 |  | down | 2.24 | 1.58E-02 |
| A_24_P944827 | *ATG7* | down | 2.23 | 4.12E-06 |
| A_24_P96403 | *RUNX1* | down | 2.23 | 2.50E-07 |
| A_23_P300174 | *HEXDC* | down | 2.23 | 1.59E-06 |
| A_32_P319880 | *KIAA1530* | down | 2.23 | 1.89E-08 |
| A_23_P100602 | *TBCD* | down | 2.23 | 1.31E-06 |
| A_23_P141770 | *NAPG* | down | 2.23 | 3.90E-05 |
| A_23_P46928 | *PFKP* | down | 2.23 | 2.19E-06 |
| A_23_P88083 | *CDC16* | down | 2.23 | 1.25E-07 |
| A_32_P54186 |  | down | 2.23 | 8.06E-03 |
| A_23_P46819 | *BTRC* | down | 2.23 | 1.15E-05 |
| A_24_P945194 | *PDCD6IP* | down | 2.23 | 1.80E-06 |
| A_24_P37540 | *TTLL3* | down | 2.23 | 1.58E-04 |
| A_24_P311845 | *PANK3* | down | 2.23 | 2.24E-03 |
| A_23_P65022 | *ACADS* | down | 2.22 | 1.64E-06 |
| A_23_P359052 | *BOD1L* | down | 2.22 | 2.55E-03 |
| A_24_P82630 | *SMCHD1* | down | 2.22 | 8.00E-03 |
| A_24_P273413 | *EML4* | down | 2.22 | 4.32E-06 |
| A_32_P122703 | *PGM2L1* | down | 2.22 | 4.17E-04 |
| A_23_P58506 | *ELL2* | down | 2.22 | 1.44E-07 |
| A_24_P46417 | *STK10* | down | 2.22 | 1.24E-07 |
| A_24_P476215 |  | down | 2.22 | 8.04E-06 |
| A_24_P508103 | *LOC440905* | down | 2.22 | 1.98E-06 |
| A_24_P50829 | *TRPM7* | down | 2.21 | 1.41E-03 |
| A_24_P80571 |  | down | 2.21 | 2.03E-06 |
| A_23_P145024 | *ADRB2* | down | 2.21 | 1.48E-08 |
| A_23_P141194 | *ICT1* | down | 2.21 | 2.90E-08 |
| A_23_P381461 | *LRRC45* | down | 2.21 | 1.58E-04 |
| A_32_P14762 | *OOEP* | down | 2.21 | 1.68E-06 |
| A_23_P72044 | *NPLOC4* | down | 2.21 | 1.01E-07 |
| A_32_P185701 |  | down | 2.21 | 7.34E-07 |
| A_32_P81806 | *ZNF738* | down | 2.21 | 1.06E-04 |
| A_24_P318967 | *PDXK* | down | 2.21 | 1.28E-06 |
| A_24_P14531 | *TM9SF4* | down | 2.21 | 4.88E-06 |
| A_23_P416034 | *HAUS7* | down | 2.20 | 1.04E-05 |
| A_23_P327370 | *PGPEP1* | down | 2.20 | 2.11E-05 |
| A_24_P34505 | *NOL10* | down | 2.20 | 3.28E-03 |
| A_32_P22355 |  | down | 2.20 | 4.07E-04 |
| A_24_P107336 | *VPS26B* | down | 2.20 | 7.72E-08 |
| A_23_P66473 | *PITPNC1* | down | 2.20 | 1.84E-06 |
| A_23_P114466 | *TBL1Y* | down | 2.20 | 1.31E-04 |
| A_24_P354900 | *DCBLD1* | down | 2.20 | 9.28E-07 |
| A_23_P24275 | *C10orf110* | down | 2.20 | 3.00E-08 |
| A_23_P201963 | *PARD3* | down | 2.20 | 7.87E-06 |
| A_32_P210223 |  | down | 2.19 | 3.90E-06 |
| A_32_P182135 |  | down | 2.19 | 1.01E-05 |
| A_32_P96124 |  | down | 2.19 | 1.01E-05 |
| A_32_P45297 |  | down | 2.19 | 5.53E-04 |
| A_23_P259797 | *ACSF3* | down | 2.19 | 7.31E-07 |
| A_24_P156490 | *KCNMA1* | down | 2.19 | 1.56E-05 |
| A_24_P936051 |  | down | 2.19 | 2.51E-03 |
| A_23_P38618 | *PIGL* | down | 2.19 | 1.50E-04 |
| A_24_P377499 | *OSBPL3* | down | 2.19 | 1.48E-06 |
| A_24_P362904 | *PFKFB4* | down | 2.19 | 1.69E-06 |
| A_23_P420692 | *PPFIA4* | down | 2.19 | 1.17E-03 |
| A_32_P208076 | *ITGA2* | down | 2.19 | 1.15E-03 |
| A_32_P406142 |  | down | 2.18 | 7.80E-08 |
| A_24_P332926 | *SFRS14* | down | 2.18 | 4.59E-07 |
| A_23_P7752 | *SEMA6A* | down | 2.18 | 8.53E-03 |
| A_23_P315206 | *CCBL1* | down | 2.18 | 1.18E-07 |
| A_23_P132226 | *TPST2* | down | 2.18 | 8.40E-06 |
| A_23_P390097 | *TTC39B* | down | 2.18 | 1.99E-04 |
| A_24_P680857 | *ATG12* | down | 2.18 | 3.63E-06 |
| A_23_P36187 | *SYT8* | down | 2.18 | 4.01E-02 |
| A_23_P143580 | *KLHL22* | down | 2.18 | 1.94E-04 |
| A_23_P106675 | *PLCG2* | down | 2.18 | 5.93E-08 |
| A_23_P341860 | *NPB* | down | 2.17 | 5.14E-06 |
| A_23_P201376 | *SSX2IP* | down | 2.17 | 3.54E-04 |
| A_23_P313223 | *C11orf84* | down | 2.17 | 1.40E-05 |
| A_23_P53081 | *OSBPL5* | down | 2.17 | 9.27E-05 |
| A_24_P135748 | *GRTP1* | down | 2.17 | 7.78E-07 |
| A_24_P927189 | *OXNAD1* | down | 2.17 | 5.29E-08 |
| A_23_P37497 | *MYO1E* | down | 2.17 | 1.80E-08 |
| A_23_P165788 | *PHOSPHO2* | down | 2.17 | 5.15E-07 |
| A_23_P204998 | *FARP1* | down | 2.16 | 1.49E-06 |
| A_32_P177300 | *ZADH2* | down | 2.16 | 3.69E-04 |
| A_32_P194563 |  | down | 2.16 | 9.36E-08 |
| A_32_P155811 | *CD2AP* | down | 2.16 | 1.32E-04 |
| A_32_P116957 |  | down | 2.16 | 1.69E-07 |
| A_24_P684721 | *TRABD* | down | 2.16 | 8.94E-07 |
| A_24_P231057 | *BOD1L* | down | 2.16 | 1.26E-03 |
| A_24_P385585 | *TMEM18* | down | 2.16 | 5.21E-03 |
| A_24_P23454 |  | down | 2.16 | 1.67E-04 |
| A_24_P113521 | *DGCR8* | down | 2.16 | 4.55E-05 |
| A_23_P15247 | *C16orf5* | down | 2.16 | 3.49E-02 |
| A_24_P941376 | *ZNF473* | down | 2.16 | 1.64E-07 |
| A_32_P919718 | *TMEM105* | down | 2.16 | 3.59E-06 |
| A_24_P245108 | *USP7* | down | 2.16 | 2.04E-04 |
| A_32_P447001 |  | down | 2.16 | 1.98E-02 |
| A_23_P135061 | *CORO2A* | down | 2.16 | 4.25E-08 |
| A_23_P253734 | *CLN8* | down | 2.15 | 1.84E-03 |
| A_24_P145229 | *CCDC123* | down | 2.15 | 5.34E-07 |
| A_23_P320837 | *STAG3L1* | down | 2.15 | 1.89E-02 |
| A_32_P161262 |  | down | 2.15 | 7.11E-04 |
| A_23_P397285 | *LY6K* | down | 2.15 | 3.99E-04 |
| A_23_P23227 | *PKN2* | down | 2.15 | 3.48E-03 |
| A_32_P30649 | *ETV5* | down | 2.15 | 1.30E-05 |
| A_23_P105833 | *BIVM* | down | 2.15 | 1.71E-04 |
| A_32_P119197 | *TPM3* | down | 2.15 | 2.78E-07 |
| A_23_P377664 | *ALS2* | down | 2.14 | 3.53E-07 |
| A_24_P93703 | *LOC440104* | down | 2.14 | 1.13E-06 |
| A_32_P27558 |  | down | 2.14 | 1.58E-04 |
| A_23_P80763 | *PVRL3* | down | 2.14 | 3.70E-06 |
| A_23_P159305 | *TAF15* | down | 2.14 | 3.23E-04 |
| A_23_P111452 | *AGAP3* | down | 2.14 | 1.43E-06 |
| A_23_P217384 | *AP1S2* | down | 2.14 | 3.44E-07 |
| A_24_P166407 | *HIST1H4B* | down | 2.14 | 2.73E-04 |
| A_32_P4495 |  | down | 2.14 | 4.67E-05 |
| A_32_P142802 |  | down | 2.14 | 3.88E-02 |
| A_23_P74778 | *C1orf54* | down | 2.13 | 4.04E-07 |
| A_23_P71598 | *MPDZ* | down | 2.13 | 4.90E-04 |
| A_23_P334664 | *PML* | down | 2.13 | 1.94E-05 |
| A_24_P388632 | *PCGF3* | down | 2.13 | 1.60E-06 |
| A_23_P374351 | *KCTD20* | down | 2.13 | 3.61E-06 |
| A_24_P226008 | *MGLL* | down | 2.13 | 1.93E-06 |
| A_23_P11774 | *UTP11L* | down | 2.13 | 4.02E-09 |
| A_23_P143748 | *TTLL12* | down | 2.13 | 6.35E-09 |
| A_24_P175059 | *ATG5* | down | 2.13 | 2.92E-04 |
| A_24_P902195 |  | down | 2.13 | 5.83E-05 |
| A_24_P58122 | *USP24* | down | 2.13 | 3.95E-08 |
| A_24_P108311 | *NEDD4L* | down | 2.13 | 6.03E-07 |
| A_23_P250619 | *ZDHHC14* | down | 2.13 | 5.28E-06 |
| A_24_P378987 | *DHRSX* | down | 2.13 | 3.86E-08 |
| A_24_P94651 | *ARIH2* | down | 2.12 | 3.82E-07 |
| A_23_P105382 | *THAP2* | down | 2.12 | 2.03E-06 |
| A_23_P165989 | *NEURL2* | down | 2.12 | 8.70E-04 |
| A_24_P914649 |  | down | 2.12 | 7.95E-07 |
| A_23_P320457 | *FAM40A* | down | 2.12 | 2.59E-06 |
| A_23_P315386 | *RHPN1* | down | 2.12 | 2.66E-06 |
| A_24_P317622 | *AGAP3* | down | 2.12 | 1.34E-04 |
| A_24_P532864 |  | down | 2.12 | 2.36E-04 |
| A_24_P333644 | *SYCP2* | down | 2.12 | 3.46E-03 |
| A_32_P114246 | *USP47* | down | 2.12 | 2.18E-05 |
| A_23_P118306 | *DNAJA3* | down | 2.12 | 2.90E-07 |
| A_23_P347432 | *DVL1* | down | 2.12 | 7.85E-06 |
| A_24_P913056 | *PLEC* | down | 2.12 | 1.65E-05 |
| A_32_P135007 |  | down | 2.11 | 1.14E-04 |
| A_23_P7684 | *CCNJL* | down | 2.11 | 9.25E-06 |
| A_24_P205316 | *DNAJC5* | down | 2.11 | 4.50E-07 |
| A_23_P144916 | *GFPT2* | down | 2.11 | 6.36E-09 |
| A_32_P32463 |  | down | 2.11 | 5.84E-05 |
| A_24_P416257 | *GGA2* | down | 2.11 | 3.93E-07 |
| A_24_P407704 | *DDX19B* | down | 2.11 | 1.68E-07 |
| A_24_P25326 | *ZMYM6* | down | 2.11 | 1.83E-07 |
| A_23_P74446 | *KDM4A* | down | 2.11 | 1.79E-03 |
| A_24_P634530 | *CPPED1* | down | 2.11 | 4.61E-05 |
| A_23_P80342 | *TAB1* | down | 2.11 | 7.06E-05 |
| A_23_P79599 | *TMEM18* | down | 2.10 | 2.66E-07 |
| A_23_P101111 | *CTDP1* | down | 2.10 | 5.08E-06 |
| A_24_P286054 | *ZFYVE16* | down | 2.10 | 9.40E-05 |
| A_23_P396062 | *RAB40C* | down | 2.10 | 3.20E-05 |
| A_24_P230808 | *BTBD7* | down | 2.10 | 9.66E-06 |
| A_23_P206059 | *PRC1* | down | 2.10 | 8.42E-08 |
| A_23_P10605 |  | down | 2.10 | 6.32E-06 |
| A_24_P389608 | *C10orf47* | down | 2.10 | 1.08E-02 |
| A_23_P61050 | *MLKL* | down | 2.10 | 6.32E-08 |
| A_23_P211302 | *WDR4* | down | 2.10 | 3.06E-04 |
| A_24_P117323 | *KLHL22* | down | 2.09 | 3.37E-06 |
| A_24_P402080 | *MBP* | down | 2.09 | 1.04E-07 |
| A_23_P93818 | *STAG3L1* | down | 2.09 | 1.90E-04 |
| A_24_P148086 | *SFRS4* | down | 2.09 | 1.75E-02 |
| A_23_P14273 | *ZFYVE21* | down | 2.09 | 2.38E-07 |
| A_23_P254801 | *PLCG1* | down | 2.09 | 3.64E-07 |
| A_24_P314554 | *GOLGA2P2* | down | 2.09 | 3.60E-02 |
| A_32_P113935 | *LNX2* | down | 2.09 | 1.80E-03 |
| A_23_P215479 | *CLIP2* | down | 2.09 | 4.52E-03 |
| A_23_P45059 | *DOCK1* | down | 2.09 | 5.10E-04 |
| A_23_P386 | *ARHGEF10L* | down | 2.09 | 4.86E-09 |
| A_23_P330727 | *PDDC1* | down | 2.09 | 8.97E-04 |
| A_32_P137604 | *ANKRD33B* | down | 2.09 | 3.66E-05 |
| A_23_P385267 | *INO80E* | down | 2.09 | 1.35E-08 |
| A_24_P598516 |  | down | 2.09 | 1.69E-02 |
| A_32_P104469 |  | down | 2.09 | 8.03E-04 |
| A_24_P153713 | *MARVELD3* | down | 2.09 | 2.15E-07 |
| A_24_P309645 | *TPCN1* | down | 2.09 | 8.85E-04 |
| A_23_P336728 | *DKFZP564C152* | down | 2.08 | 4.50E-02 |
| A_24_P76635 | *ACTR3C* | down | 2.08 | 2.51E-05 |
| A_23_P40989 | *USP13* | down | 2.08 | 7.30E-07 |
| A_24_P743806 | *ZNF618* | down | 2.08 | 8.69E-03 |
| A_32_P32653 | *SENP5* | down | 2.08 | 3.30E-07 |
| A_24_P859032 |  | down | 2.08 | 3.17E-08 |
| A_23_P70480 | *HIST1H4L* | down | 2.08 | 2.73E-03 |
| A_23_P404821 | *KIAA1147* | down | 2.08 | 2.69E-06 |
| A_23_P9465 | *FPGS* | down | 2.08 | 9.97E-07 |
| A_32_P164246 | *FOXQ1* | down | 2.07 | 1.91E-05 |
| A_23_P250564 | *PRKCE* | down | 2.07 | 1.01E-06 |
| A_23_P60283 | *XPA* | down | 2.07 | 5.11E-08 |
| A_23_P256603 | *MLLT4* | down | 2.07 | 7.19E-05 |
| A_24_P235266 | *GRB10* | down | 2.07 | 2.25E-05 |
| A_24_P364381 | *MMAB* | down | 2.07 | 3.71E-04 |
| A_23_P204269 | *USP15* | down | 2.07 | 3.34E-05 |
| A_23_P401774 | *ELMOD1* | down | 2.07 | 5.91E-03 |
| A_23_P413641 | *PREX1* | down | 2.07 | 4.44E-03 |
| A_23_P8848 | *INTS9* | down | 2.07 | 4.84E-07 |
| A_23_P200043 | *KIAA0562* | down | 2.07 | 1.97E-05 |
| A_24_P453740 | *DNAJC21* | down | 2.07 | 4.04E-06 |
| A_24_P56052 | *ZFP91* | down | 2.07 | 2.52E-04 |
| A_32_P208868 |  | down | 2.07 | 2.15E-06 |
| A_32_P155841 |  | down | 2.07 | 1.94E-07 |
| A_23_P205370 | *ASB2* | down | 2.07 | 6.05E-07 |
| A_23_P163955 | *PEMT* | down | 2.07 | 1.75E-08 |
| A_23_P112774 | *PTP4A3* | down | 2.06 | 2.23E-02 |
| A_24_P408341 | *CRYZL1* | down | 2.06 | 2.55E-03 |
| A_23_P217804 | *CDK11B* | down | 2.06 | 1.03E-02 |
| A_23_P48936 | *SMAD3* | down | 2.06 | 4.21E-09 |
| A_24_P922465 |  | down | 2.06 | 1.67E-03 |
| A_24_P339974 | *AP2A2* | down | 2.06 | 4.80E-07 |
| A_32_P103464 |  | down | 2.06 | 2.64E-03 |
| A_23_P103433 | *OSCP1* | down | 2.06 | 9.65E-09 |
| A_23_P65918 | *ITPKA* | down | 2.06 | 6.32E-06 |
| A_24_P88554 | *PEX11B* | down | 2.06 | 4.46E-05 |
| A_23_P134637 | *KIAA0415* | down | 2.06 | 1.77E-06 |
| A_23_P211829 | *ZXDC* | down | 2.06 | 2.84E-06 |
| A_23_P134477 | *C7orf50* | down | 2.06 | 4.49E-05 |
| A_23_P132405 | *ACAD9* | down | 2.06 | 3.23E-07 |
| A_24_P943106 | *SR140* | down | 2.06 | 7.18E-04 |
| A_23_P46315 | *DENND2C* | down | 2.05 | 9.07E-05 |
| A_32_P132317 | *GPR155* | down | 2.05 | 1.05E-02 |
| A_24_P404245 | *PCYT2* | down | 2.05 | 5.41E-06 |
| A_23_P431410 | *RBMS1* | down | 2.05 | 2.01E-07 |
| A_23_P354591 | *FAM125B* | down | 2.05 | 2.84E-02 |
| A_23_P14216 | *C13orf38* | down | 2.05 | 1.51E-05 |
| A_23_P132619 | *OXTR* | down | 2.05 | 1.22E-03 |
| A_24_P406814 | *FAM53B* | down | 2.05 | 1.84E-06 |
| A_32_P20912 |  | down | 2.05 | 7.51E-07 |
| A_23_P5654 | *IL1F7* | down | 2.05 | 8.61E-05 |
| A_24_P555510 | *PCM1* | down | 2.05 | 1.91E-05 |
| A_24_P323610 | *MYO1F* | down | 2.05 | 5.39E-03 |
| A_23_P37244 | *SNAPC1* | down | 2.05 | 1.25E-07 |
| A_32_P59606 |  | down | 2.05 | 7.87E-05 |
| A_23_P252681 | *PCYT1A* | down | 2.05 | 5.14E-06 |
| A_24_P80204 | *MALL* | down | 2.04 | 3.34E-08 |
| A_24_P336931 | *ANKRD36* | down | 2.04 | 2.20E-08 |
| A_23_P217120 | *EHMT1* | down | 2.04 | 2.23E-07 |
| A_23_P165548 | *HS1BP3* | down | 2.04 | 3.55E-05 |
| A_24_P944751 | *C22orf9* | down | 2.04 | 2.29E-07 |
| A_23_P200843 | *CHRM3* | down | 2.04 | 1.13E-04 |
| A_23_P64888 | *TAS2R10* | down | 2.04 | 2.07E-04 |
| A_32_P26376 | *KTELC1* | down | 2.04 | 1.41E-05 |
| A_32_P226700 |  | down | 2.04 | 1.52E-07 |
| A_32_P218707 |  | down | 2.04 | 1.35E-05 |
| A_23_P365119 |  | down | 2.04 | 8.35E-03 |
| A_32_P11894 | *C12orf65* | down | 2.04 | 9.50E-04 |
| A_23_P37505 | *DYX1C1* | down | 2.04 | 2.50E-09 |
| A_23_P345212 | *BOD1P* | down | 2.04 | 3.01E-08 |
| A_32_P94722 | *BTBD9* | down | 2.04 | 3.36E-07 |
| A_23_P21853 | *AP2A2* | down | 2.04 | 1.79E-08 |
| A_24_P16892 | *TAF2* | down | 2.03 | 6.13E-03 |
| A_24_P106057 | *PCYT1A* | down | 2.03 | 1.79E-07 |
| A_24_P193257 |  | down | 2.03 | 3.56E-06 |
| A_23_P56422 | *LRRFIP1* | down | 2.03 | 1.52E-04 |
| A_23_P216468 | *SLC1A1* | down | 2.03 | 2.31E-07 |
| A_23_P302654 | *CEP72* | down | 2.03 | 8.49E-09 |
| A_24_P347418 | *LRCH1* | down | 2.03 | 1.01E-07 |
| A_23_P158277 | *TMCO4* | down | 2.03 | 1.60E-05 |
| A_24_P185314 | *NAPG* | down | 2.03 | 3.28E-06 |
| A_23_P212781 |  | down | 2.03 | 8.85E-04 |
| A_32_P216004 |  | down | 2.03 | 1.66E-03 |
| A_23_P351679 | *TK2* | down | 2.03 | 1.61E-07 |
| A_24_P363087 | *C5orf45* | down | 2.03 | 5.53E-06 |
| A_24_P152743 | *TMC6* | down | 2.03 | 2.37E-06 |
| A_32_P154731 |  | down | 2.03 | 5.21E-04 |
| A_23_P342108 | *CCDC116* | down | 2.03 | 8.01E-04 |
| A_24_P362540 | *ASAP2* | down | 2.03 | 5.76E-07 |
| A_24_P744297 |  | down | 2.03 | 2.35E-04 |
| A_23_P35030 | *CPSF3L* | down | 2.03 | 4.69E-06 |
| A_23_P87329 | *NAT10* | down | 2.02 | 2.77E-05 |
| A_23_P394216 | *TECPR2* | down | 2.02 | 9.00E-08 |
| A_32_P109637 |  | down | 2.02 | 1.05E-04 |
| A_23_P139418 | *GALNTL4* | down | 2.02 | 3.74E-04 |
| A_23_P120153 | *RNF149* | down | 2.02 | 4.88E-06 |
| A_24_P346181 | *FAM120B* | down | 2.02 | 1.57E-04 |
| A_23_P316974 | *SYNJ2* | down | 2.02 | 6.36E-05 |
| A_32_P218332 | *ACSF3* | down | 2.02 | 4.22E-06 |
| A_24_P400997 | *SMCHD1* | down | 2.02 | 1.89E-02 |
| A_24_P216501 | *PRKAR1B* | down | 2.02 | 8.06E-05 |
| A_24_P219094 | *SIPA1L1* | down | 2.02 | 1.91E-04 |
| A_23_P61406 | *SHC3* | down | 2.02 | 1.49E-07 |
| A_23_P72680 | *IFT81* | down | 2.02 | 1.15E-02 |
| A_24_P418138 | *LOC100129503* | down | 2.02 | 9.79E-04 |
| A_23_P420942 | *MT1E* | down | 2.02 | 3.41E-06 |
| A_24_P328504 | *SP140* | down | 2.02 | 5.33E-05 |
| A_32_P202066 |  | down | 2.01 | 1.91E-04 |
| A_24_P408321 | *OSBPL2* | down | 2.01 | 1.05E-06 |
| A_24_P216253 | *DLGAP4* | down | 2.01 | 7.42E-04 |
| A_23_P24751 | *TTC9C* | down | 2.01 | 1.38E-04 |
| A_23_P48387 | *PDS5B* | down | 2.01 | 5.83E-04 |
| A_32_P134698 |  | down | 2.01 | 1.18E-05 |
| A_24_P26897 | *INPP5A* | down | 2.01 | 1.65E-08 |
| A_23_P218317 | *NARF* | down | 2.01 | 1.49E-07 |
| A_24_P797455 |  | down | 2.01 | 1.15E-05 |
| A_24_P74571 | *CBY1* | down | 2.01 | 4.72E-06 |
| A_23_P51187 | *PRKCZ* | down | 2.01 | 1.49E-07 |
| A_23_P168567 | *GTF2I* | down | 2.01 | 3.98E-08 |
| A_23_P436526 | *SLC25A42* | down | 2.01 | 1.60E-04 |
| A_23_P20793 |  | down | 2.01 | 2.62E-05 |
| A_23_P210554 | *SPATA2* | down | 2.01 | 7.58E-03 |
| A_24_P755169 |  | down | 2.00 | 2.36E-07 |
| A_24_P175435 | *SLC2A8* | down | 2.00 | 4.08E-06 |
| A_24_P577694 | *ADCY1* | down | 2.00 | 2.81E-02 |
| A_23_P364324 | *ABCA13* | down | 2.00 | 1.95E-03 |
| A_24_P235131 | *RNF216* | down | 2.00 | 1.06E-04 |
| A_24_P182764 | *ATG4B* | down | 2.00 | 1.57E-05 |
| A_23_P100556 | *EXOC7* | down | 2.00 | 2.33E-05 |
| A_24_P260361 | *BBS7* | down | 2.00 | 1.22E-05 |
| A_24_P926125 | *LOC390705* | down | 2.00 | 5.46E-07 |
| A_24_P339858 | *C21orf90* | down | 2.00 | 1.69E-07 |
| A_24_P83102 | *IGLL1* | down | 2.00 | 1.40E-04 |
| A_32_P10067 |  | down | 2.00 | 2.56E-09 |
| A_23_P306804 | *CITED4* | down | 2.00 | 1.22E-03 |
| A_32_P174908 | *WAPAL* | down | 2.00 | 2.36E-07 |
| A_23_P212844 | *TACC3* | down | 2.00 | 8.27E-06 |
| A_23_P377888 | *MTMR15* | down | 2.00 | 6.55E-07 |
| A_24_P51786 | *CCDC50* | down | 2.00 | 1.76E-03 |
| A_23_P125157 | *NFATC2IP* | down | 2.00 | 2.15E-05 |
| A_32_P235840 |  | up | 82.77 | 1.60E-07 |
| A_23_P58137 |  | up | 46.61 | 4.22E-10 |
| A_32_P118655 | *CTBP1* | up | 36.08 | 9.77E-11 |
| A_23_P365685 | *LIMS3* | up | 31.99 | 6.17E-11 |
| A_32_P77139 |  | up | 29.38 | 8.87E-09 |
| A_32_P232883 |  | up | 19.95 | 2.01E-09 |
| A_24_P924836 | *CRYZL1* | up | 18.12 | 1.91E-07 |
| A_24_P917280 | *ALDH1A3* | up | 14.87 | 2.06E-07 |
| A_23_P388762 | *GPR107* | up | 13.41 | 5.53E-13 |
| A_23_P417372 | *ZSCAN2* | up | 12.98 | 1.53E-08 |
| A_24_P475864 | *RRN3P2* | up | 12.68 | 2.33E-07 |
| A_24_P162145 |  | up | 12.20 | 8.78E-07 |
| A_24_P497843 |  | up | 11.22 | 1.49E-07 |
| A_32_P234336 |  | up | 10.76 | 2.44E-04 |
| A_32_P184667 |  | up | 10.19 | 1.94E-05 |
| A_24_P532478 |  | up | 10.14 | 1.52E-08 |
| A_24_P93736 | *FLJ40292* | up | 10.03 | 1.15E-06 |
| A_24_P514678 |  | up | 10.02 | 2.87E-07 |
| A_24_P229389 | *DKFZP686I15217* | up | 9.84 | 1.76E-06 |
| A_24_P699286 | *LOC399900* | up | 9.09 | 3.30E-06 |
| A_32_P26800 |  | up | 9.08 | 4.42E-07 |
| A_24_P136691 | *C7orf50* | up | 8.94 | 4.72E-10 |
| A_23_P130764 | *KCNJ14* | up | 8.80 | 1.17E-09 |
| A_24_P924183 | *MYO10* | up | 8.72 | 1.63E-09 |
| A_23_P329271 | *MC1R* | up | 8.52 | 2.68E-07 |
| A_24_P936999 | *ZNF462* | up | 8.37 | 3.56E-07 |
| A_23_P417891 | *ARHGEF7* | up | 8.29 | 2.70E-04 |
| A_23_P301328 | *PDXK* | up | 7.86 | 7.13E-09 |
| A_24_P919989 | *ABCC5* | up | 7.64 | 1.11E-07 |
| A_32_P217471 | *MYO1B* | up | 7.60 | 3.47E-07 |
| A_24_P212875 |  | up | 7.58 | 1.14E-07 |
| A_23_P430800 | *CCDC17* | up | 7.55 | 1.43E-07 |
| A_32_P116488 |  | up | 7.46 | 7.53E-08 |
| A_24_P76868 |  | up | 7.32 | 3.01E-07 |
| A_32_P150152 |  | up | 7.26 | 3.06E-07 |
| A_23_P359173 | *VPS13B* | up | 7.19 | 1.21E-07 |
| A_23_P66095 | *MC1R* | up | 7.05 | 7.58E-06 |
| A_32_P3131 |  | up | 7.03 | 1.92E-07 |
| A_23_P251965 | *ZNF273* | up | 7.02 | 2.31E-07 |
| A_32_P142550 | *ZNF562* | up | 6.94 | 6.63E-08 |
| A_23_P1102 | *ACTA1* | up | 6.90 | 1.33E-06 |
| A_23_P34396 | *C1orf63* | up | 6.85 | 2.01E-09 |
| A_24_P7143 | *MYO1B* | up | 6.85 | 6.35E-09 |
| A_23_P133739 | *HUS1B* | up | 6.79 | 8.62E-07 |
| A_24_P123521 | *CLK4* | up | 6.77 | 3.36E-07 |
| A_24_P295330 | *TSTD2* | up | 6.70 | 6.83E-07 |
| A_24_P24256 | *MGC24125* | up | 6.67 | 4.87E-06 |
| A_24_P81695 | *SNX19* | up | 6.62 | 4.51E-09 |
| A_24_P398810 | *EIF5* | up | 6.51 | 3.19E-09 |
| A_24_P917934 | *U2AF1* | up | 6.48 | 2.41E-08 |
| A_23_P96688 | *SUV420H1* | up | 6.43 | 9.08E-06 |
| A_24_P940233 |  | up | 6.37 | 1.82E-05 |
| A_23_P500433 | *CARD9* | up | 6.35 | 1.97E-08 |
| A_24_P930985 | *RXRA* | up | 6.29 | 1.39E-07 |
| A_23_P134980 | *FGD3* | up | 6.23 | 3.94E-03 |
| A_24_P315535 | *SPRED2* | up | 6.07 | 2.20E-03 |
| A_24_P933706 |  | up | 6.04 | 5.51E-07 |
| A_23_P257834 | *ALB* | up | 6.04 | 9.14E-06 |
| A_24_P367752 | *NDST1* | up | 5.95 | 2.21E-09 |
| A_23_P302028 | *DYNLRB1* | up | 5.91 | 2.24E-06 |
| A_23_P351215 | *SKIL* | up | 5.90 | 2.60E-07 |
| A_32_P95034 |  | up | 5.82 | 1.93E-08 |
| A_32_P8813 | *LOC283663* | up | 5.77 | 1.17E-09 |
| A_23_P209011 | *BRD4* | up | 5.75 | 3.37E-06 |
| A_32_P102300 |  | up | 5.72 | 1.31E-08 |
| A_24_P273510 | *LOC100130078* | up | 5.67 | 8.41E-06 |
| A_23_P131502 | *TTL* | up | 5.62 | 4.03E-09 |
| A_24_P782102 |  | up | 5.60 | 8.41E-06 |
| A_23_P357504 |  | up | 5.56 | 4.02E-09 |
| A_32_P120791 | *LOC727916* | up | 5.56 | 1.09E-07 |
| A_23_P200439 | *ADAR* | up | 5.56 | 4.26E-08 |
| A_24_P914573 | *SULT1A3* | up | 5.48 | 5.16E-06 |
| A_23_P251962 | *ZNF273* | up | 5.46 | 4.02E-09 |
| A_24_P717586 |  | up | 5.43 | 5.78E-08 |
| A_23_P103756 | *OVGP1* | up | 5.35 | 1.93E-08 |
| A_23_P144668 | *CMBL* | up | 5.30 | 3.92E-04 |
| A_23_P423974 | *HUS1B* | up | 5.23 | 4.88E-07 |
| A_32_P169243 |  | up | 5.19 | 3.11E-05 |
| A_24_P558034 |  | up | 5.18 | 5.82E-06 |
| A_24_P223163 | *NAF1* | up | 5.16 | 1.51E-05 |
| A_24_P109272 | *ADD1* | up | 5.16 | 3.41E-08 |
| A_24_P230675 | *SOCS2* | up | 5.06 | 1.03E-06 |
| A_24_P924166 | *NANP* | up | 5.06 | 1.11E-05 |
| A_32_P170811 | *CCDC88C* | up | 5.05 | 6.83E-07 |
| A_24_P932648 |  | up | 5.02 | 4.16E-08 |
| A_23_P132138 | *C21orf58* | up | 4.98 | 2.01E-09 |
| A_23_P65262 | *N4BP2L2* | up | 4.94 | 2.49E-08 |
| A_24_P823011 |  | up | 4.93 | 9.40E-10 |
| A_24_P36425 | *YTHDF1* | up | 4.91 | 1.06E-08 |
| A_23_P125639 | *ZFX* | up | 4.88 | 4.59E-05 |
| A_23_P20122 | *ZC3HAV1* | up | 4.86 | 2.97E-09 |
| A_24_P941947 | *LPCAT1* | up | 4.82 | 2.01E-05 |
| A_24_P81691 | *SNX19* | up | 4.66 | 1.72E-06 |
| A_23_P143514 | *C21orf122* | up | 4.64 | 8.06E-06 |
| A_32_P162862 |  | up | 4.59 | 2.15E-08 |
| A_24_P916808 | *CRTC3* | up | 4.57 | 2.69E-02 |
| A_24_P927639 |  | up | 4.46 | 1.41E-08 |
| A_24_P751247 |  | up | 4.39 | 3.59E-06 |
| A_24_P927716 | *UNQ9368* | up | 4.39 | 1.80E-04 |
| A_24_P167012 | *TNFSF15* | up | 4.38 | 1.16E-02 |
| A_24_P16361 |  | up | 4.37 | 2.31E-03 |
| A_24_P247987 | *C16orf57* | up | 4.37 | 2.86E-08 |
| A_24_P479793 |  | up | 4.35 | 1.21E-07 |
| A_23_P357248 | *ZNF343* | up | 4.30 | 3.08E-07 |
| A_24_P152398 | *TP53AIP1* | up | 4.30 | 1.25E-04 |
| A_24_P917021 |  | up | 4.30 | 3.43E-08 |
| A_23_P206998 | *MTMR4* | up | 4.28 | 8.79E-07 |
| A_24_P608302 | *LOC388796* | up | 4.25 | 2.01E-10 |
| A_23_P20927 | *TNKS* | up | 4.25 | 8.00E-04 |
| A_23_P138105 | *MED18* | up | 4.23 | 3.89E-03 |
| A_23_P85932 |  | up | 4.22 | 2.24E-05 |
| A_23_P41664 | *LRRC70* | up | 4.21 | 3.92E-09 |
| A_24_P935782 | *ZNF121* | up | 4.21 | 8.18E-09 |
| A_24_P358976 | *TMEM19* | up | 4.20 | 1.49E-08 |
| A_24_P257151 | *CLK1* | up | 4.19 | 1.62E-05 |
| A_32_P121085 | *DOK3* | up | 4.17 | 2.86E-09 |
| A_23_P351744 |  | up | 4.13 | 4.68E-07 |
| A_24_P233944 | *CEPT1* | up | 4.11 | 3.53E-08 |
| A_23_P90933 | *DUSP19* | up | 4.10 | 2.09E-04 |
| A_32_P81324 |  | up | 4.08 | 1.95E-05 |
| A_23_P171237 | *ACRC* | up | 4.07 | 4.76E-05 |
| A_23_P46690 | *TMEM81* | up | 4.06 | 4.64E-06 |
| A_24_P63290 | *C3orf62* | up | 4.04 | 8.35E-06 |
| A_23_P127002 | *THNSL1* | up | 4.03 | 3.84E-04 |
| A_32_P110751 | *EIF4A2* | up | 4.03 | 1.29E-06 |
| A_24_P879787 | *LOC642826* | up | 4.02 | 3.33E-09 |
| A_24_P169048 | *RFPL3S* | up | 4.01 | 1.04E-04 |
| A_24_P857624 | *LOC100131096* | up | 3.99 | 6.36E-09 |
| A_24_P59569 | *LOC400684* | up | 3.97 | 2.97E-09 |
| A_23_P103269 |  | up | 3.96 | 9.86E-09 |
| A_24_P934679 |  | up | 3.93 | 4.22E-10 |
| A_24_P68908 | *LOC344887* | up | 3.93 | 2.04E-05 |
| A_24_P913016 | *UROS* | up | 3.92 | 5.93E-08 |
| A_24_P282172 | *TRIP11* | up | 3.90 | 2.46E-05 |
| A_23_P3792 | *SLC7A5* | up | 3.88 | 1.84E-07 |
| A_24_P339436 | *GNAL* | up | 3.88 | 1.26E-07 |
| A_24_P342020 |  | up | 3.87 | 1.88E-05 |
| A_24_P332721 |  | up | 3.87 | 4.10E-03 |
| A_32_P115707 |  | up | 3.87 | 4.29E-08 |
| A_24_P410378 | *FTSJD1* | up | 3.85 | 1.51E-05 |
| A_24_P341938 | *ZC3HAV1* | up | 3.85 | 4.44E-08 |
| A_32_P210723 |  | up | 3.83 | 1.70E-04 |
| A_24_P376787 | *ZNF496* | up | 3.82 | 1.75E-06 |
| A_32_P232381 |  | up | 3.79 | 1.40E-07 |
| A_23_P330017 | *DOLPP1* | up | 3.77 | 1.70E-03 |
| A_24_P119685 | *OBSCN* | up | 3.77 | 3.40E-07 |
| A_24_P935125 | *ZNF23* | up | 3.76 | 1.70E-04 |
| A_23_P422204 | *PPPDE1* | up | 3.74 | 3.14E-06 |
| A_23_P355993 | *ZNF614* | up | 3.74 | 2.20E-03 |
| A_24_P378302 | *C15orf28* | up | 3.73 | 1.87E-06 |
| A_24_P359165 | *SWAP70* | up | 3.73 | 2.78E-07 |
| A_23_P46660 |  | up | 3.72 | 5.17E-07 |
| A_24_P867342 | *AFMID* | up | 3.71 | 4.04E-06 |
| A_24_P316475 | *TTF2* | up | 3.71 | 1.98E-05 |
| A_23_P426565 | *C17orf55* | up | 3.70 | 7.48E-07 |
| A_32_P80523 |  | up | 3.70 | 2.96E-05 |
| A_32_P209624 | *LOC100240726* | up | 3.68 | 2.30E-05 |
| A_24_P911571 | *GNB5* | up | 3.64 | 4.51E-09 |
| A_23_P126100 | *INADL* | up | 3.61 | 3.91E-07 |
| A_24_P823381 |  | up | 3.60 | 1.94E-04 |
| A_23_P90021 | *ZNF557* | up | 3.57 | 7.58E-08 |
| A_24_P177604 | *PPP1R3F* | up | 3.57 | 3.38E-10 |
| A_23_P41674 | *GRPEL2* | up | 3.57 | 3.11E-08 |
| A_23_P210807 | *CSTL1* | up | 3.56 | 1.82E-04 |
| A_23_P114349 | *XAGE3* | up | 3.54 | 3.07E-04 |
| A_24_P342023 |  | up | 3.52 | 1.40E-07 |
| A_23_P385911 | *KIAA1712* | up | 3.52 | 4.94E-05 |
| A_23_P399797 | *SMAD5OS* | up | 3.52 | 6.58E-05 |
| A_24_P615822 | *BMS1* | up | 3.50 | 3.38E-10 |
| A_32_P224234 | *LOC645195* | up | 3.50 | 1.99E-10 |
| A_23_P54692 |  | up | 3.48 | 1.55E-06 |
| A_32_P159254 |  | up | 3.48 | 9.53E-06 |
| A_32_P215318 | *ACACA* | up | 3.48 | 7.18E-08 |
| A_24_P289170 | *CCDC88C* | up | 3.48 | 4.06E-09 |
| A_24_P155058 | *IKBKB* | up | 3.47 | 1.63E-05 |
| A_24_P93316 | *LOC96610* | up | 3.45 | 4.85E-06 |
| A_23_P206501 | *CLEC18B* | up | 3.44 | 3.69E-06 |
| A_32_P21459 |  | up | 3.44 | 4.05E-06 |
| A_23_P316460 | *C7orf29* | up | 3.44 | 4.89E-08 |
| A_24_P338121 | *UBQLN1* | up | 3.44 | 7.71E-03 |
| A_23_P427334 |  | up | 3.43 | 6.03E-05 |
| A_24_P693946 |  | up | 3.43 | 3.29E-08 |
| A_32_P125568 |  | up | 3.42 | 2.27E-08 |
| A_24_P114936 | *GM2A* | up | 3.41 | 7.07E-04 |
| A_24_P860662 | *LOC100290415* | up | 3.40 | 1.85E-05 |
| A_23_P385084 | *LUZP1* | up | 3.40 | 7.08E-08 |
| A_24_P128145 | *ATF2* | up | 3.39 | 2.71E-05 |
| A_32_P33434 | *ZNF812* | up | 3.39 | 4.03E-02 |
| A_23_P377339 | *C4orf36* | up | 3.38 | 1.51E-04 |
| A_32_P199998 | *C10orf75* | up | 3.37 | 6.02E-09 |
| A_23_P309207 | *ZNF577* | up | 3.36 | 1.39E-06 |
| A_32_P130522 |  | up | 3.35 | 7.71E-09 |
| A_23_P390677 | *MECOM* | up | 3.34 | 7.79E-07 |
| A_23_P154962 | *RIMBP3* | up | 3.33 | 9.97E-06 |
| A_23_P34877 | *RBM15* | up | 3.33 | 7.84E-07 |
| A_32_P197976 |  | up | 3.31 | 1.63E-06 |
| A_23_P378588 | *ARL5B* | up | 3.31 | 1.90E-04 |
| A_32_P9931 |  | up | 3.30 | 7.11E-08 |
| A_23_P53458 | *CORO1C* | up | 3.30 | 6.50E-09 |
| A_24_P29594 | *HBS1L* | up | 3.29 | 9.04E-04 |
| A_24_P67784 | *RIMBP3* | up | 3.29 | 5.54E-07 |
| A_24_P2584 | *C1orf63* | up | 3.29 | 1.46E-08 |
| A_23_P104741 | *KIRREL3* | up | 3.28 | 9.58E-05 |
| A_23_P74391 | *OPN3* | up | 3.28 | 3.31E-05 |
| A_23_P201248 | *SLC26A9* | up | 3.27 | 5.50E-06 |
| A_23_P80008 | *MYLK2* | up | 3.27 | 4.39E-06 |
| A_24_P162412 | *WDR20* | up | 3.25 | 2.94E-05 |
| A_32_P23319 |  | up | 3.25 | 2.99E-07 |
| A_23_P27247 | *MED13* | up | 3.23 | 7.74E-06 |
| A_23_P211136 | *BRWD1* | up | 3.23 | 1.28E-07 |
| A_24_P826348 | *ZC3H6* | up | 3.21 | 6.09E-07 |
| A_32_P90685 |  | up | 3.20 | 7.11E-08 |
| A_23_P309991 | *BCL2L11* | up | 3.20 | 1.65E-08 |
| A_23_P100469 | *TXNL4B* | up | 3.19 | 2.72E-06 |
| A_23_P129717 | *ERI2* | up | 3.19 | 1.26E-04 |
| A_23_P425954 | *BCL2L11* | up | 3.16 | 2.33E-05 |
| A_24_P268196 | *LZIC* | up | 3.15 | 2.72E-09 |
| A_23_P412554 | *C18orf25* | up | 3.14 | 1.62E-06 |
| A_23_P218646 | *TNFRSF6B* | up | 3.14 | 1.01E-07 |
| A_24_P339450 | *ZNF24* | up | 3.14 | 2.02E-05 |
| A_24_P359838 | *TTL* | up | 3.13 | 6.49E-08 |
| A_24_P228266 | *ZNF304* | up | 3.12 | 4.61E-05 |
| A_24_P268993 | *LEAP2* | up | 3.12 | 3.00E-08 |
| A_23_P104237 | *GPAM* | up | 3.12 | 3.76E-04 |
| A_24_P920938 |  | up | 3.12 | 2.02E-06 |
| A_24_P276628 | *PPT1* | up | 3.11 | 2.60E-07 |
| A_23_P166929 | *SERPINI1* | up | 3.11 | 3.90E-05 |
| A_23_P393645 | *ADAMTS13* | up | 3.10 | 9.82E-09 |
| A_32_P109214 | *CCDC144NL* | up | 3.10 | 2.41E-08 |
| A_23_P135294 | *ALDH1B1* | up | 3.08 | 4.79E-07 |
| A_23_P256047 | *ANKRD5* | up | 3.08 | 2.79E-09 |
| A_24_P138784 | *MGAT5B* | up | 3.07 | 2.80E-07 |
| A_24_P915806 | *HNMT* | up | 3.07 | 1.09E-08 |
| A_32_P187985 |  | up | 3.06 | 1.02E-04 |
| A_23_P251730 | *ATP11C* | up | 3.05 | 3.45E-04 |
| A_23_P143143 | *ID2* | up | 3.05 | 1.33E-03 |
| A_24_P36457 | *STX16* | up | 3.05 | 2.69E-06 |
| A_23_P15580 | *ANKRD40* | up | 3.05 | 6.14E-08 |
| A_23_P203601 | *UCP3* | up | 3.05 | 4.39E-09 |
| A_24_P337575 | *FAM8A1* | up | 3.05 | 1.79E-05 |
| A_23_P365060 | *MDN1* | up | 3.04 | 1.79E-05 |
| A_23_P62465 | *ZFY* | up | 3.03 | 5.72E-09 |
| A_23_P16817 | *CLK1* | up | 3.02 | 3.41E-07 |
| A_23_P120270 | *MCFD2* | up | 3.01 | 1.34E-07 |
| A_32_P167148 | *PIGW* | up | 3.01 | 6.36E-06 |
| A_32_P93459 |  | up | 3.00 | 1.01E-04 |
| A_24_P914240 | *ZNF44* | up | 2.99 | 7.39E-04 |
| A_24_P65949 | *PDXK* | up | 2.98 | 4.01E-06 |
| A_23_P86741 |  | up | 2.97 | 1.40E-04 |
| A_24_P387514 | *LRP5L* | up | 2.97 | 1.41E-07 |
| A_23_P128911 |  | up | 2.97 | 1.13E-02 |
| A_23_P12950 | *KBTBD4* | up | 2.96 | 1.62E-05 |
| A_24_P740692 |  | up | 2.96 | 7.01E-07 |
| A_23_P124946 | *CMYA5* | up | 2.95 | 9.77E-04 |
| A_24_P375453 | *SSBP3* | up | 2.94 | 1.57E-08 |
| A_23_P50195 |  | up | 2.93 | 9.40E-10 |
| A_23_P171388 | *TMSB4Y* | up | 2.93 | 5.94E-04 |
| A_24_P218001 | *ZNF273* | up | 2.92 | 1.13E-06 |
| A_24_P147252 | *ZNF23* | up | 2.92 | 1.32E-05 |
| A_23_P8640 | *GPER* | up | 2.92 | 4.58E-08 |
| A_24_P934162 |  | up | 2.91 | 4.71E-08 |
| A_23_P168974 | *SDCBP* | up | 2.91 | 1.53E-05 |
| A_24_P202512 | *RNF138* | up | 2.91 | 5.66E-06 |
| A_23_P160367 | *THRAP3* | up | 2.90 | 1.76E-06 |
| A_23_P209316 | *CCNT2* | up | 2.90 | 1.68E-05 |
| A_24_P919840 |  | up | 2.90 | 1.16E-07 |
| A_32_P115122 |  | up | 2.90 | 1.80E-08 |
| A_24_P935400 |  | up | 2.90 | 2.33E-03 |
| A_24_P192821 | *TRIM44* | up | 2.90 | 5.28E-06 |
| A_32_P117464 | *C3orf59* | up | 2.89 | 1.27E-08 |
| A_32_P179706 |  | up | 2.89 | 6.74E-05 |
| A_23_P38100 | *TRIM65* | up | 2.88 | 3.27E-07 |
| A_23_P34066 | *IL9R* | up | 2.88 | 7.02E-05 |
| A_23_P214779 | *UTRN* | up | 2.88 | 1.29E-08 |
| A_32_P138977 | *SERINC4* | up | 2.87 | 3.45E-07 |
| A_23_P48705 | *PTGR2* | up | 2.87 | 7.98E-08 |
| A_23_P379794 | *PIGW* | up | 2.87 | 1.17E-09 |
| A_24_P389285 | *C7orf54* | up | 2.87 | 1.37E-02 |
| A_24_P262407 | *THRA* | up | 2.86 | 2.12E-06 |
| A_23_P377245 |  | up | 2.86 | 8.08E-03 |
| A_23_P365086 | *ANKRD5* | up | 2.85 | 4.17E-08 |
| A_23_P49610 | *C17orf91* | up | 2.84 | 4.39E-09 |
| A_24_P276816 | *RCOR3* | up | 2.83 | 1.58E-09 |
| A_23_P18384 | *ARMC8* | up | 2.83 | 5.81E-08 |
| A_23_P155351 | *BTD* | up | 2.82 | 1.76E-04 |
| A_24_P401432 | *LOC100128343* | up | 2.82 | 4.01E-06 |
| A_23_P58835 | *F2RL1* | up | 2.82 | 5.93E-04 |
| A_23_P208288 | *ZNF304* | up | 2.81 | 5.75E-07 |
| A_32_P110016 | *LOC727869* | up | 2.81 | 2.62E-05 |
| A_23_P85765 | *CACNA1S* | up | 2.81 | 1.38E-04 |
| A_32_P64716 |  | up | 2.81 | 1.13E-06 |
| A_23_P63870 | *SAMD8* | up | 2.79 | 1.27E-06 |
| A_24_P757154 |  | up | 2.79 | 2.01E-04 |
| A_23_P334635 | *JRK* | up | 2.79 | 2.48E-06 |
| A_23_P204654 | *KITLG* | up | 2.79 | 3.60E-08 |
| A_23_P58382 | *LIN54* | up | 2.78 | 1.34E-05 |
| A_32_P37461 |  | up | 2.77 | 6.02E-06 |
| A_32_P154872 |  | up | 2.77 | 2.15E-08 |
| A_23_P166219 | *GABPA* | up | 2.77 | 5.17E-07 |
| A_23_P128215 | *SOCS2* | up | 2.77 | 2.45E-08 |
| A_24_P117866 | *ADD1* | up | 2.77 | 1.01E-09 |
| A_24_P201728 | *C12orf32* | up | 2.76 | 7.06E-07 |
| A_23_P331992 | *STX6* | up | 2.75 | 1.23E-08 |
| A_23_P121250 | *EIF4A2* | up | 2.75 | 3.39E-09 |
| A_23_P38830 | *ZNF552* | up | 2.74 | 1.02E-06 |
| A_23_P415061 | *FAM104B* | up | 2.74 | 5.18E-04 |
| A_23_P396299 | *LOC100129827* | up | 2.74 | 1.59E-08 |
| A_24_P401241 | *ZNF160* | up | 2.74 | 2.18E-03 |
| A_32_P437227 | *FAM84B* | up | 2.74 | 1.79E-04 |
| A_23_P327698 | *LMBRD2* | up | 2.73 | 3.92E-05 |
| A_23_P407115 | *PIP4K2B* | up | 2.73 | 2.09E-06 |
| A_32_P167208 |  | up | 2.73 | 3.73E-04 |
| A_24_P935252 | *FXR1* | up | 2.73 | 3.00E-07 |
| A_23_P75083 | *ZMYND11* | up | 2.73 | 4.83E-09 |
| A_23_P110725 | *PRKAA1* | up | 2.72 | 1.08E-07 |
| A_24_P109854 | *ZNF468* | up | 2.72 | 2.04E-04 |
| A_32_P215866 |  | up | 2.72 | 4.16E-07 |
| A_24_P902728 | *CAPRIN1* | up | 2.72 | 2.18E-07 |
| A_24_P674479 | *FAM122C* | up | 2.72 | 5.04E-04 |
| A_24_P152404 | *C10orf76* | up | 2.71 | 2.16E-07 |
| A_23_P136724 | *LOC344887* | up | 2.71 | 1.20E-04 |
| A_23_P373100 | *MGC24103* | up | 2.71 | 1.99E-04 |
| A_24_P272313 | *C2orf55* | up | 2.70 | 1.30E-02 |
| A_23_P207742 | *THRA* | up | 2.70 | 1.58E-05 |
| A_24_P105391 | *ERMAP* | up | 2.69 | 3.07E-06 |
| A_23_P121939 | *PTCD2* | up | 2.69 | 1.07E-07 |
| A_23_P416751 | *ZNF610* | up | 2.69 | 1.01E-08 |
| A_32_P66306 | *LOC400604* | up | 2.69 | 1.51E-04 |
| A_23_P348349 |  | up | 2.68 | 1.08E-03 |
| A_24_P7950 | *VAV3* | up | 2.68 | 5.29E-06 |
| A_24_P202357 | *PNPO* | up | 2.68 | 3.39E-03 |
| A_24_P8151 | *LOC100131170* | up | 2.67 | 1.13E-06 |
| A_24_P912228 | *LOC100131053* | up | 2.67 | 2.65E-06 |
| A_32_P144208 |  | up | 2.66 | 1.08E-04 |
| A_24_P135489 | *TSPAN14* | up | 2.66 | 4.70E-03 |
| A_23_P105583 | *C12orf32* | up | 2.66 | 2.80E-07 |
| A_23_P309996 | *BCL2L11* | up | 2.65 | 5.80E-07 |
| A_24_P306810 | *RNF213* | up | 2.65 | 1.27E-04 |
| A_32_P117758 |  | up | 2.65 | 4.57E-07 |
| A_23_P11598 | *ZNF684* | up | 2.65 | 1.72E-03 |
| A_24_P558135 |  | up | 2.65 | 8.21E-07 |
| A_23_P205828 | *TJP1* | up | 2.65 | 7.17E-08 |
| A_23_P423480 | *TYSND1* | up | 2.64 | 1.43E-06 |
| A_32_P135733 |  | up | 2.64 | 8.03E-04 |
| A_24_P210513 | *BTBD7* | up | 2.63 | 1.68E-06 |
| A_23_P414308 | *FLCN* | up | 2.63 | 3.29E-07 |
| A_23_P107942 | *ZNF473* | up | 2.63 | 8.96E-08 |
| A_24_P370484 |  | up | 2.62 | 2.97E-08 |
| A_24_P655833 | *LOC100289602* | up | 2.62 | 1.58E-05 |
| A_23_P21278 | *MTBP* | up | 2.62 | 1.24E-02 |
| A_24_P337867 | *ORAI2* | up | 2.62 | 5.80E-07 |
| A_32_P208136 | *C6orf167* | up | 2.62 | 7.09E-05 |
| A_24_P212826 | *ZNF525* | up | 2.62 | 5.96E-05 |
| A_23_P312252 | *C15orf40* | up | 2.61 | 2.50E-04 |
| A_23_P254790 | *TAF9B* | up | 2.60 | 1.02E-06 |
| A_32_P161033 |  | up | 2.60 | 9.55E-05 |
| A_23_P326204 | *SGMS2* | up | 2.60 | 3.21E-07 |
| A_23_P216199 | *PPP1R3B* | up | 2.60 | 7.19E-05 |
| A_23_P106391 | *THAP10* | up | 2.60 | 2.47E-07 |
| A_23_P206877 | *RRN3* | up | 2.60 | 4.85E-07 |
| A_32_P176550 | *JMY* | up | 2.60 | 2.01E-08 |
| A_23_P13222 | *RCN1* | up | 2.60 | 5.99E-08 |
| A_23_P18102 | *SENP5* | up | 2.59 | 5.09E-07 |
| A_24_P321093 | *SPOCK2* | up | 2.59 | 5.28E-03 |
| A_23_P41854 | *CARD6* | up | 2.59 | 3.61E-05 |
| A_24_P85317 | *CHD2* | up | 2.59 | 3.11E-04 |
| A_24_P884696 |  | up | 2.59 | 3.48E-03 |
| A_32_P157465 |  | up | 2.59 | 5.13E-05 |
| A_24_P225477 | *ECHDC2* | up | 2.59 | 2.08E-06 |
| A_23_P501583 | *NFYA* | up | 2.59 | 1.80E-07 |
| A_24_P230895 | *SETMAR* | up | 2.59 | 6.99E-03 |
| A_24_P380284 | *PCDHB9* | up | 2.59 | 1.27E-04 |
| A_23_P59375 | *ID4* | up | 2.58 | 1.01E-06 |
| A_32_P203836 | *GEN1* | up | 2.58 | 1.76E-06 |
| A_23_P50232 | *AURKC* | up | 2.58 | 1.24E-02 |
| A_32_P69368 | *ID2* | up | 2.57 | 1.90E-06 |
| A_23_P347040 | *DTX3L* | up | 2.57 | 8.18E-09 |
| A_32_P3998 | *ZNF813* | up | 2.57 | 4.47E-08 |
| A_23_P156310 | *SKP2* | up | 2.57 | 1.97E-08 |
| A_23_P393749 | *CATSPER3* | up | 2.56 | 5.72E-07 |
| A_23_P95972 | *TXNL4B* | up | 2.56 | 3.11E-08 |
| A_24_P914479 |  | up | 2.56 | 2.90E-08 |
| A_32_P139219 |  | up | 2.56 | 1.61E-04 |
| A_32_P202719 |  | up | 2.56 | 1.83E-05 |
| A_23_P208915 | *DPP9* | up | 2.55 | 1.00E-07 |
| A_23_P38813 | *ZNF320* | up | 2.55 | 9.09E-06 |
| A_32_P22549 |  | up | 2.54 | 3.14E-04 |
| A_32_P84640 |  | up | 2.54 | 4.89E-05 |
| A_24_P670342 |  | up | 2.54 | 7.32E-04 |
| A_32_P84846 | *ZNF780B* | up | 2.53 | 3.78E-08 |
| A_23_P217899 | *CCNL2* | up | 2.53 | 3.84E-07 |
| A_24_P532212 |  | up | 2.53 | 4.72E-10 |
| A_24_P247587 | *LOC348751* | up | 2.53 | 1.85E-02 |
| A_24_P339059 | *CRISPLD2* | up | 2.53 | 2.45E-04 |
| A_32_P10231 |  | up | 2.52 | 1.23E-04 |
| A_23_P29499 | *CTNNB1* | up | 2.52 | 1.91E-05 |
| A_23_P502488 | *ATG4C* | up | 2.52 | 4.47E-07 |
| A_24_P940524 | *ZFX* | up | 2.52 | 1.42E-06 |
| A_24_P49775 | *ZNF493* | up | 2.52 | 8.15E-08 |
| A_24_P187954 | *ARMC8* | up | 2.51 | 5.51E-07 |
| A_23_P15692 | *GPR172B* | up | 2.51 | 2.24E-07 |
| A_24_P189739 | *DUSP16* | up | 2.51 | 1.43E-06 |
| A_23_P87773 | *C12orf48* | up | 2.51 | 8.53E-03 |
| A_24_P64895 |  | up | 2.51 | 9.07E-05 |
| A_23_P333330 | *ZDHHC24* | up | 2.51 | 7.47E-08 |
| A_23_P108129 | *ZNF223* | up | 2.50 | 2.85E-03 |
| A_23_P20480 | *BRF2* | up | 2.50 | 3.62E-07 |
| A_23_P126120 | *CENPL* | up | 2.49 | 1.79E-05 |
| A_23_P258931 | *ZNF623* | up | 2.49 | 1.20E-07 |
| A_23_P436138 | *MAX* | up | 2.49 | 3.66E-07 |
| A_23_P385851 | *MBNL3* | up | 2.49 | 6.12E-07 |
| A_23_P165521 | *STRN* | up | 2.49 | 4.39E-05 |
| A_24_P14328 | *ZNF564* | up | 2.49 | 6.56E-05 |
| A_23_P397376 | *MAF* | up | 2.49 | 8.62E-08 |
| A_24_P281243 |  | up | 2.49 | 2.99E-07 |
| A_24_P358400 |  | up | 2.48 | 1.19E-04 |
| A_32_P172920 |  | up | 2.48 | 1.25E-08 |
| A_32_P178746 |  | up | 2.48 | 1.38E-05 |
| A_32_P120567 | *MDFIC* | up | 2.48 | 1.20E-05 |
| A_23_P314202 | *PAPD4* | up | 2.48 | 7.68E-08 |
| A_23_P305335 | *RBM8A* | up | 2.48 | 5.88E-05 |
| A_24_P358493 | *NCRNA00171* | up | 2.47 | 2.29E-04 |
| A_24_P917711 | *PRKAB2* | up | 2.47 | 2.09E-04 |
| A_32_P378278 | *FAM117B* | up | 2.47 | 1.61E-05 |
| A_24_P924462 | *PRKCZ* | up | 2.47 | 5.26E-05 |
| A_23_P209760 | *AMMECR1L* | up | 2.47 | 2.87E-04 |
| A_23_P55584 | *RIOK3* | up | 2.47 | 2.74E-08 |
| A_32_P64418 | *DIS3L2* | up | 2.47 | 2.56E-07 |
| A_23_P113005 | *EFNA1* | up | 2.47 | 2.97E-09 |
| A_24_P275984 | *ZNF616* | up | 2.46 | 2.20E-05 |
| A_24_P137713 | *ZNF323* | up | 2.46 | 3.48E-05 |
| A_24_P178602 | *ZNF600* | up | 2.46 | 1.53E-05 |
| A_24_P419017 |  | up | 2.45 | 1.82E-06 |
| A_23_P161197 | *SEC61A2* | up | 2.45 | 5.38E-05 |
| A_24_P920271 |  | up | 2.45 | 1.57E-05 |
| A_32_P197060 |  | up | 2.45 | 6.07E-04 |
| A_23_P319895 | *SETD1B* | up | 2.45 | 8.54E-06 |
| A_24_P931755 |  | up | 2.45 | 1.23E-03 |
| A_32_P199002 |  | up | 2.45 | 1.66E-02 |
| A_23_P169494 | *ORM1* | up | 2.44 | 1.81E-06 |
| A_23_P122655 | *FLJ13744* | up | 2.44 | 6.68E-05 |
| A_23_P71830 | *ZBTB26* | up | 2.43 | 2.11E-05 |
| A_32_P207428 | *ZNF845* | up | 2.43 | 1.64E-07 |
| A_23_P4640 | *ZNF548* | up | 2.43 | 1.02E-06 |
| A_32_P153833 |  | up | 2.43 | 4.39E-09 |
| A_24_P370670 | *ZMYM6* | up | 2.43 | 4.04E-05 |
| A_32_P73139 | *LOC100289602* | up | 2.43 | 2.35E-06 |
| A_24_P925737 | *C1orf226* | up | 2.43 | 4.86E-05 |
| A_23_P49459 | *LOC81691* | up | 2.43 | 3.78E-05 |
| A_24_P83944 | *ZNF586* | up | 2.43 | 1.95E-07 |
| A_23_P250118 | *HSPBAP1* | up | 2.43 | 1.02E-08 |
| A_32_P34046 | *HFM1* | up | 2.42 | 9.00E-03 |
| A_23_P65068 | *EID3* | up | 2.42 | 1.32E-05 |
| A_24_P820087 |  | up | 2.42 | 8.58E-08 |
| A_32_P44316 | *EEF1A1* | up | 2.42 | 6.58E-04 |
| A_23_P16806 | *hCG_1993592* | up | 2.42 | 3.91E-07 |
| A_24_P355693 | *ACER3* | up | 2.41 | 1.28E-05 |
| A_32_P15017 | *RICTOR* | up | 2.41 | 1.17E-04 |
| A_24_P337140 | *ABHD14B* | up | 2.41 | 1.68E-07 |
| A_24_P932208 | *CREB1* | up | 2.41 | 1.52E-03 |
| A_24_P265342 |  | up | 2.41 | 1.13E-05 |
| A_32_P125803 |  | up | 2.40 | 2.11E-05 |
| A_24_P489649 |  | up | 2.40 | 1.02E-03 |
| A_24_P881527 | *CTNND1* | up | 2.40 | 3.09E-03 |
| A_24_P722360 |  | up | 2.40 | 2.07E-03 |
| A_24_P727501 |  | up | 2.40 | 3.39E-07 |
| A_32_P129800 | *FLJ39653* | up | 2.40 | 1.32E-02 |
| A_23_P12010 | *C1orf66* | up | 2.40 | 6.99E-08 |
| A_24_P316154 |  | up | 2.40 | 4.94E-09 |
| A_32_P158524 |  | up | 2.39 | 2.26E-06 |
| A_23_P134026 | *C6orf35* | up | 2.39 | 1.21E-06 |
| A_32_P122402 | *LOC441455* | up | 2.39 | 3.34E-07 |
| A_24_P919995 |  | up | 2.39 | 9.28E-04 |
| A_23_P334271 |  | up | 2.39 | 2.89E-05 |
| A_23_P128940 | *C14orf138* | up | 2.39 | 4.06E-09 |
| A_23_P38567 | *CYTSB* | up | 2.39 | 8.09E-06 |
| A_23_P74928 | *MR1* | up | 2.39 | 1.65E-08 |
| A_32_P117760 |  | up | 2.38 | 4.36E-09 |
| A_23_P17471 | *FAM113A* | up | 2.38 | 7.42E-06 |
| A_23_P95417 | *TNFRSF10D* | up | 2.38 | 2.90E-06 |
| A_23_P52219 | *ERLIN1* | up | 2.38 | 4.02E-09 |
| A_32_P89277 |  | up | 2.38 | 4.71E-07 |
| A_32_P95573 |  | up | 2.38 | 3.45E-07 |
| A_23_P167201 | *SEPSECS* | up | 2.38 | 1.49E-02 |
| A_24_P101261 |  | up | 2.38 | 8.30E-05 |
| A_32_P98136 | *KIAA1107* | up | 2.38 | 2.78E-06 |
| A_23_P362261 | *FDXACB1* | up | 2.38 | 8.46E-06 |
| A_23_P83234 | *ZBTB6* | up | 2.37 | 5.74E-03 |
| A_32_P130968 |  | up | 2.37 | 4.07E-07 |
| A_32_P185682 |  | up | 2.37 | 1.24E-02 |
| A_23_P146379 | *RANBP6* | up | 2.37 | 6.08E-05 |
| A_23_P398040 | *YTHDF3* | up | 2.37 | 5.88E-06 |
| A_24_P921232 | *PEX14* | up | 2.37 | 1.30E-05 |
| A_24_P226076 | *METAP1* | up | 2.37 | 3.39E-05 |
| A_23_P83320 | *GARNL3* | up | 2.37 | 1.03E-04 |
| A_23_P500886 | *CLDN15* | up | 2.36 | 1.06E-05 |
| A_23_P430764 | *CDC42SE2* | up | 2.36 | 5.04E-06 |
| A_32_P219753 | *POLR2D* | up | 2.36 | 9.67E-06 |
| A_24_P934473 | *LOC100132006* | up | 2.36 | 1.10E-03 |
| A_23_P93082 | *NUS1* | up | 2.36 | 1.52E-07 |
| A_24_P835943 |  | up | 2.36 | 3.65E-06 |
| A_32_P212920 |  | up | 2.36 | 3.70E-04 |
| A_24_P714134 | *LOC399881* | up | 2.36 | 1.85E-04 |
| A_23_P13033 | *RBM4* | up | 2.36 | 9.86E-09 |
| A_24_P323434 | *CDCA2* | up | 2.35 | 2.53E-04 |
| A_32_P9468 | *COX19* | up | 2.35 | 1.43E-07 |
| A_24_P13376 | *ADIPOR2* | up | 2.35 | 2.09E-06 |
| A_24_P375510 |  | up | 2.35 | 2.12E-06 |
| A_23_P118536 | *SLFN12* | up | 2.35 | 2.45E-08 |
| A_23_P62159 | *FAM120C* | up | 2.35 | 5.95E-07 |
| A_32_P133926 |  | up | 2.35 | 4.21E-06 |
| A_32_P397824 | *FIGN* | up | 2.35 | 5.04E-04 |
| A_23_P40535 | *CRKL* | up | 2.34 | 2.45E-03 |
| A_23_P78342 | *LMAN1* | up | 2.34 | 5.54E-07 |
| A_23_P341392 | *CYB5D2* | up | 2.34 | 9.65E-09 |
| A_24_P307665 |  | up | 2.34 | 1.55E-09 |
| A_24_P571937 |  | up | 2.34 | 7.98E-04 |
| A_24_P164838 | *SPATA2* | up | 2.33 | 8.03E-06 |
| A_23_P167767 | *CDC42SE2* | up | 2.33 | 1.25E-07 |
| A_24_P937644 | *CDK11A* | up | 2.33 | 2.02E-05 |
| A_24_P246767 | *LOC643387* | up | 2.33 | 7.11E-04 |
| A_24_P256539 | *C12orf47* | up | 2.33 | 1.43E-07 |
| A_24_P106297 | *AMACR* | up | 2.33 | 5.41E-08 |
| A_32_P223441 |  | up | 2.33 | 3.59E-06 |
| A_23_P301336 | *C10orf28* | up | 2.33 | 1.89E-03 |
| A_24_P157087 | *CASP8* | up | 2.33 | 1.88E-05 |
| A_23_P155049 | *APOL6* | up | 2.33 | 5.17E-04 |
| A_24_P388659 | *ARHGAP24* | up | 2.32 | 7.57E-05 |
| A_23_P200890 | *GOLPH3L* | up | 2.32 | 1.73E-07 |
| A_23_P123503 | *TRIB1* | up | 2.32 | 1.93E-06 |
| A_23_P205247 | *RCOR1* | up | 2.32 | 1.64E-03 |
| A_24_P205120 | *SPG7* | up | 2.32 | 7.53E-08 |
| A_23_P147826 | *RAP2C* | up | 2.32 | 5.73E-06 |
| A_23_P113825 | *NACC2* | up | 2.32 | 1.03E-07 |
| A_24_P392022 | *FAM86A* | up | 2.32 | 3.05E-06 |
| A_24_P933372 |  | up | 2.32 | 8.49E-05 |
| A_23_P215517 | *KLHL7* | up | 2.32 | 3.42E-09 |
| A_24_P766204 | *MAP3K1* | up | 2.32 | 3.02E-03 |
| A_24_P714620 |  | up | 2.32 | 4.18E-02 |
| A_23_P5464 | *RQCD1* | up | 2.31 | 6.34E-08 |
| A_23_P257755 | *RMND5A* | up | 2.31 | 7.98E-05 |
| A_23_P6490 | *TNRC6B* | up | 2.31 | 1.04E-07 |
| A_24_P472081 |  | up | 2.31 | 2.32E-05 |
| A_23_P217763 | *TMSB4X* | up | 2.31 | 3.47E-06 |
| A_23_P201035 | *GBA* | up | 2.31 | 5.21E-04 |
| A_24_P58147 | *LMBRD2* | up | 2.31 | 2.56E-05 |
| A_23_P114095 | *MBTPS2* | up | 2.30 | 3.72E-07 |
| A_23_P39766 | *GLS* | up | 2.30 | 6.14E-08 |
| A_24_P99371 | *THUMPD1* | up | 2.30 | 6.29E-07 |
| A_23_P91217 | *CHD6* | up | 2.30 | 6.27E-07 |
| A_23_P203201 | *DDX6* | up | 2.30 | 8.14E-06 |
| A_23_P156708 | *TNXB* | up | 2.30 | 1.83E-05 |
| A_23_P426472 | *ZNF45* | up | 2.30 | 2.31E-08 |
| A_23_P254025 | *ZFP37* | up | 2.30 | 5.94E-04 |
| A_23_P71709 | *LOC402377* | up | 2.29 | 1.17E-02 |
| A_23_P44569 | *ABCC2* | up | 2.29 | 5.95E-05 |
| A_24_P722292 |  | up | 2.29 | 3.91E-08 |
| A_23_P422268 | *DCAF7* | up | 2.29 | 5.23E-06 |
| A_23_P205611 | *GMFB* | up | 2.29 | 3.10E-04 |
| A_23_P48713 | *PTGR2* | up | 2.29 | 1.03E-08 |
| A_32_P207436 | *ZNF845* | up | 2.29 | 1.64E-07 |
| A_24_P8304 |  | up | 2.29 | 1.01E-05 |
| A_23_P501435 | *CSRP2BP* | up | 2.29 | 2.54E-07 |
| A_23_P430758 | *CDC42SE2* | up | 2.29 | 1.63E-06 |
| A_24_P201153 | *TJP2* | up | 2.29 | 3.55E-05 |
| A_24_P50139 |  | up | 2.28 | 3.23E-04 |
| A_23_P57137 | *C20orf29* | up | 2.28 | 6.81E-07 |
| A_23_P147238 | *WSB2* | up | 2.28 | 2.63E-04 |
| A_32_P178099 |  | up | 2.28 | 1.16E-08 |
| A_23_P67980 | *KLF7* | up | 2.28 | 2.97E-09 |
| A_23_P151970 | *FEM1B* | up | 2.28 | 3.20E-06 |
| A_23_P207068 | *USP22* | up | 2.28 | 4.51E-03 |
| A_32_P140220 |  | up | 2.28 | 1.22E-06 |
| A_23_P56298 | *ZNF430* | up | 2.27 | 2.52E-04 |
| A_32_P129419 | *NARG2* | up | 2.27 | 8.44E-04 |
| A_24_P766228 |  | up | 2.27 | 6.40E-06 |
| A_24_P385341 | *C1orf107* | up | 2.27 | 3.17E-09 |
| A_23_P209538 | *KIAA1310* | up | 2.27 | 1.25E-07 |
| A_32_P82424 | *LOC647252* | up | 2.27 | 7.18E-04 |
| A_23_P14174 | *TNFSF13B* | up | 2.27 | 3.37E-04 |
| A_24_P48617 | *UQCR11* | up | 2.26 | 1.05E-07 |
| A_23_P421011 | *KAZALD1* | up | 2.26 | 2.96E-07 |
| A_23_P255591 | *ZNF268* | up | 2.26 | 9.81E-08 |
| A_24_P401830 | *LOC100129196* | up | 2.26 | 1.74E-04 |
| A_32_P367241 | *DUSP19* | up | 2.26 | 1.39E-06 |
| A_24_P385148 | *ZNF197* | up | 2.25 | 1.88E-06 |
| A_24_P263297 | *CALHM3* | up | 2.25 | 2.50E-09 |
| A_24_P400140 | *NFS1* | up | 2.25 | 7.01E-07 |
| A_24_P390833 | *MPPE1* | up | 2.25 | 4.11E-06 |
| A_23_P6786 |  | up | 2.25 | 4.43E-07 |
| A_23_P73096 |  | up | 2.25 | 7.68E-04 |
| A_23_P136493 | *NRG1* | up | 2.25 | 9.09E-08 |
| A_24_P222835 | *S100PBP* | up | 2.25 | 1.55E-07 |
| A_23_P150667 | *KIF18A* | up | 2.25 | 2.73E-07 |
| A_23_P319512 | *KLF11* | up | 2.25 | 2.92E-05 |
| A_24_P145124 | *CEP57* | up | 2.25 | 4.00E-06 |
| A_23_P22915 | *SLC30A7* | up | 2.25 | 2.40E-05 |
| A_32_P311737 | *PLEKHG3* | up | 2.25 | 4.70E-05 |
| A_23_P9435 | *SLC25A25* | up | 2.25 | 2.52E-05 |
| A_23_P26729 | *COIL* | up | 2.24 | 3.44E-06 |
| A_23_P15844 | *BRIP1* | up | 2.24 | 7.74E-06 |
| A_24_P219920 | *FEM1A* | up | 2.24 | 5.36E-07 |
| A_32_P181548 |  | up | 2.24 | 2.29E-03 |
| A_23_P65041 | *RACGAP1P* | up | 2.24 | 2.15E-06 |
| A_23_P55639 | *RPRD1A* | up | 2.23 | 4.76E-05 |
| A_32_P2392 | *GOLGA8A* | up | 2.23 | 3.62E-07 |
| A_24_P228027 | *CYB5D2* | up | 2.23 | 8.39E-08 |
| A_24_P66685 | *NAA30* | up | 2.23 | 9.09E-08 |
| A_23_P315815 | *NRG1* | up | 2.22 | 1.51E-07 |
| A_23_P167066 | *UGDH* | up | 2.22 | 4.66E-06 |
| A_23_P127851 | *FXC1* | up | 2.22 | 5.64E-08 |
| A_23_P431939 | *MR1* | up | 2.22 | 3.33E-09 |
| A_23_P28397 |  | up | 2.22 | 1.96E-02 |
| A_23_P501634 | *BTN2A1* | up | 2.22 | 4.35E-03 |
| A_24_P200652 | *C6orf62* | up | 2.22 | 1.22E-06 |
| A_24_P91222 | *DCAF7* | up | 2.22 | 2.08E-07 |
| A_23_P165390 | *SLC30A6* | up | 2.22 | 1.18E-07 |
| A_24_P221407 | *PYROXD1* | up | 2.22 | 1.36E-05 |
| A_23_P137381 | *ID3* | up | 2.21 | 7.27E-10 |
| A_24_P358146 | *LOC100129478* | up | 2.21 | 6.19E-07 |
| A_24_P349648 |  | up | 2.21 | 1.44E-03 |
| A_24_P277211 | *HRH1* | up | 2.21 | 1.04E-07 |
| A_23_P20732 | *GTF3C4* | up | 2.21 | 5.51E-07 |
| A_23_P424316 | *TCF20* | up | 2.21 | 4.91E-06 |
| A_23_P96590 | *GPRASP1* | up | 2.21 | 1.58E-08 |
| A_32_P39991 |  | up | 2.21 | 2.66E-03 |
| A_23_P355455 | *TBC1D5* | up | 2.21 | 1.49E-08 |
| A_32_P145639 |  | up | 2.21 | 1.77E-06 |
| A_23_P339601 | *ZNF578* | up | 2.21 | 1.05E-05 |
| A_23_P257043 | *GEM* | up | 2.20 | 1.57E-05 |
| A_24_P89509 | *TRMT11* | up | 2.20 | 1.15E-03 |
| A_23_P74252 | *HSPC157* | up | 2.20 | 9.73E-06 |
| A_23_P334630 | *JRK* | up | 2.20 | 6.49E-08 |
| A_24_P199929 | *VPS54* | up | 2.20 | 3.61E-04 |
| A_24_P272608 | *ZNF808* | up | 2.20 | 2.50E-04 |
| A_23_P322593 | *FLJ39653* | up | 2.19 | 7.04E-05 |
| A_23_P142407 | *ZNF101* | up | 2.19 | 1.04E-07 |
| A_23_P121141 | *ASTE1* | up | 2.19 | 6.83E-07 |
| A_24_P415280 | *SEC61A2* | up | 2.19 | 1.55E-06 |
| A_24_P66592 | *CNKSR3* | up | 2.19 | 1.65E-03 |
| A_23_P48628 | *ZBTB25* | up | 2.19 | 3.63E-06 |
| A_24_P101800 | *LOC284804* | up | 2.19 | 6.50E-09 |
| A_24_P107208 | *CAPRIN1* | up | 2.19 | 2.56E-05 |
| A_23_P382688 | *FAM122B* | up | 2.18 | 1.53E-04 |
| A_24_P932680 | *LOC100289230* | up | 2.18 | 2.48E-03 |
| A_24_P39759 | *RNF135* | up | 2.18 | 6.30E-04 |
| A_23_P254512 | *EFNA1* | up | 2.18 | 6.08E-06 |
| A_24_P393470 | *LOC729991* | up | 2.18 | 2.24E-05 |
| A_23_P134366 | *ETV1* | up | 2.18 | 7.39E-07 |
| A_23_P146004 | *CBLL1* | up | 2.18 | 4.03E-06 |
| A_24_P272389 |  | up | 2.18 | 1.07E-03 |
| A_23_P56630 | *STAT1* | up | 2.18 | 4.91E-05 |
| A_23_P353574 | *NEK7* | up | 2.18 | 6.19E-04 |
| A_32_P44775 | *C9orf85* | up | 2.18 | 1.32E-07 |
| A_32_P95015 |  | up | 2.18 | 3.54E-02 |
| A_23_P163353 | *PIAS1* | up | 2.18 | 2.01E-09 |
| A_23_P407142 | *LUZP1* | up | 2.17 | 1.10E-05 |
| A_24_P230965 | *KIAA1586* | up | 2.17 | 6.83E-04 |
| A_24_P228026 | *CYB5D2* | up | 2.17 | 9.62E-07 |
| A_23_P10927 | *TM2D2* | up | 2.17 | 8.18E-08 |
| A_23_P208055 | *LPIN2* | up | 2.17 | 2.87E-02 |
| A_23_P40354 | *MAPRE1* | up | 2.17 | 7.18E-08 |
| A_23_P51117 | *ETAA1* | up | 2.17 | 8.58E-08 |
| A_24_P88870 | *B3GALNT1* | up | 2.17 | 7.47E-04 |
| A_32_P63324 |  | up | 2.17 | 8.57E-07 |
| A_23_P61674 | *CLK4* | up | 2.16 | 6.82E-06 |
| A_23_P25121 | *FKBP11* | up | 2.16 | 9.06E-07 |
| A_32_P155043 |  | up | 2.16 | 4.94E-05 |
| A_24_P261488 | *SAPS3* | up | 2.16 | 1.10E-04 |
| A_24_P41391 | *COX19* | up | 2.16 | 2.30E-06 |
| A_23_P9932 | *PDCD4* | up | 2.15 | 2.60E-04 |
| A_23_P25888 | *FRMD6* | up | 2.15 | 2.62E-04 |
| A_23_P74309 | *NOS1AP* | up | 2.15 | 2.78E-03 |
| A_23_P334263 | *SENP8* | up | 2.15 | 4.48E-05 |
| A_23_P39755 | *B3GNT7* | up | 2.15 | 2.15E-06 |
| A_24_P377775 | *RGS3* | up | 2.15 | 4.71E-05 |
| A_24_P136653 | *NFE2L3* | up | 2.15 | 2.12E-03 |
| A_23_P167067 | *UGDH* | up | 2.15 | 1.13E-04 |
| A_24_P204971 | *HBP1* | up | 2.15 | 2.14E-07 |
| A_24_P349821 | *MTRF1* | up | 2.15 | 1.58E-03 |
| A_23_P116376 | *CPSF7* | up | 2.15 | 8.38E-06 |
| A_23_P356041 | *SPAG9* | up | 2.14 | 8.14E-04 |
| A_24_P33895 | *ATF3* | up | 2.14 | 2.35E-04 |
| A_23_P31389 | *TRA2A* | up | 2.14 | 1.17E-04 |
| A_24_P410389 | *BCL2L13* | up | 2.14 | 3.93E-05 |
| A_24_P261691 | *HIST4H4* | up | 2.14 | 5.76E-04 |
| A_32_P11969 |  | up | 2.14 | 2.00E-04 |
| A_23_P155417 | *ABHD14B* | up | 2.14 | 4.94E-09 |
| A_23_P18246 | *XCR1* | up | 2.14 | 1.18E-06 |
| A_23_P74162 | *IQCC* | up | 2.14 | 1.17E-08 |
| A_24_P316414 | *LOC100289058* | up | 2.14 | 3.23E-03 |
| A_23_P347169 | *MTUS1* | up | 2.14 | 9.74E-04 |
| A_23_P347508 | *C1orf163* | up | 2.13 | 1.45E-07 |
| A_23_P104509 | *FAM53B* | up | 2.13 | 4.16E-03 |
| A_23_P362228 | *C1orf213* | up | 2.13 | 2.71E-08 |
| A_32_P217655 | *LOC645166* | up | 2.13 | 2.89E-07 |
| A_23_P206697 | *CYB5B* | up | 2.13 | 4.92E-06 |
| A_24_P398585 | *UNG* | up | 2.13 | 2.99E-04 |
| A_23_P353436 | *CEP78* | up | 2.13 | 2.49E-05 |
| A_24_P150676 | *TRAPPC2L* | up | 2.13 | 2.82E-06 |
| A_23_P404595 | *ZNF417* | up | 2.13 | 2.21E-03 |
| A_23_P384825 | *LOC100303728* | up | 2.12 | 5.58E-03 |
| A_24_P230708 | *PDE12* | up | 2.12 | 6.34E-06 |
| A_32_P174130 |  | up | 2.12 | 1.46E-05 |
| A_24_P298744 |  | up | 2.12 | 1.96E-02 |
| A_23_P115885 | *MINPP1* | up | 2.12 | 4.73E-03 |
| A_24_P159323 | *GINS3* | up | 2.12 | 1.46E-05 |
| A_23_P59294 | *RREB1* | up | 2.12 | 1.20E-05 |
| A_32_P228886 |  | up | 2.12 | 1.46E-06 |
| A_23_P54389 | *NARG2* | up | 2.12 | 3.82E-06 |
| A_23_P385861 | *CDCA2* | up | 2.12 | 3.33E-07 |
| A_24_P702749 |  | up | 2.12 | 1.25E-04 |
| A_24_P916494 | *PPA2* | up | 2.11 | 5.21E-05 |
| A_23_P257753 | *RMND5A* | up | 2.11 | 6.87E-07 |
| A_24_P213622 | *LOC100129113* | up | 2.11 | 6.06E-04 |
| A_32_P34926 |  | up | 2.11 | 1.55E-05 |
| A_24_P215628 | *DCUN1D2* | up | 2.11 | 3.05E-06 |
| A_23_P151376 |  | up | 2.11 | 4.39E-09 |
| A_23_P317654 | *DDX3X* | up | 2.11 | 6.64E-05 |
| A_32_P203219 | *MAP4K5* | up | 2.11 | 1.35E-07 |
| A_23_P36825 | *GPRC5A* | up | 2.11 | 6.83E-06 |
| A_23_P258814 | *DPH3B* | up | 2.11 | 5.80E-07 |
| A_23_P166159 | *PDRG1* | up | 2.11 | 7.86E-09 |
| A_24_P102389 | *ALG11* | up | 2.11 | 4.39E-09 |
| A_24_P940218 | *PPP2R2D* | up | 2.11 | 5.84E-07 |
| A_23_P371011 | *ZNF227* | up | 2.11 | 3.83E-07 |
| A_32_P32061 | *C2orf27A* | up | 2.10 | 2.00E-06 |
| A_24_P393958 | *DNAJB4* | up | 2.10 | 3.90E-05 |
| A_32_P235796 | *BBS12* | up | 2.10 | 3.30E-05 |
| A_24_P326739 | *GLS2* | up | 2.10 | 1.87E-02 |
| A_23_P100344 | *ORC6L* | up | 2.10 | 2.16E-07 |
| A_24_P359191 | *SLC6A6* | up | 2.10 | 7.44E-04 |
| A_24_P76898 | *PPP2R5C* | up | 2.10 | 4.48E-05 |
| A_32_P82462 | *LOC554202* | up | 2.10 | 2.77E-04 |
| A_32_P480177 | *TNN* | up | 2.10 | 4.58E-03 |
| A_24_P244699 | *NUDT15* | up | 2.10 | 6.98E-05 |
| A_32_P107208 | *C1orf106* | up | 2.10 | 8.11E-03 |
| A_23_P167263 | *PHF17* | up | 2.09 | 1.17E-05 |
| A_23_P209778 | *POLR2D* | up | 2.09 | 6.50E-09 |
| A_23_P71111 | *C7orf64* | up | 2.09 | 1.72E-04 |
| A_32_P219368 | *WTAP* | up | 2.09 | 6.86E-05 |
| A_24_P74329 | *ZNF493* | up | 2.09 | 9.92E-07 |
| A_23_P208069 | *METTL4* | up | 2.09 | 3.68E-06 |
| A_24_P922357 | *C22orf39* | up | 2.09 | 9.08E-06 |
| A_24_P393461 | *C1orf43* | up | 2.09 | 2.78E-05 |
| A_24_P941166 | *ZNF425* | up | 2.09 | 2.73E-04 |
| A_24_P212086 | *SERPINB5* | up | 2.09 | 4.65E-04 |
| A_24_P206624 | *FGFR2* | up | 2.09 | 2.80E-02 |
| A_24_P342086 | *WWP2* | up | 2.08 | 8.43E-06 |
| A_23_P155868 | *PGRMC2* | up | 2.08 | 4.12E-07 |
| A_24_P810084 |  | up | 2.08 | 1.59E-06 |
| A_32_P6972 |  | up | 2.08 | 4.50E-05 |
| A_32_P108592 |  | up | 2.08 | 1.25E-06 |
| A_23_P16242 | *ZNF20* | up | 2.08 | 4.14E-03 |
| A_23_P145463 | *SLC35B3* | up | 2.08 | 1.52E-06 |
| A_23_P140780 |  | up | 2.08 | 8.70E-04 |
| A_32_P115701 | *NARG2* | up | 2.08 | 3.63E-05 |
| A_24_P319635 | *MCL1* | up | 2.08 | 1.68E-07 |
| A_32_P35294 |  | up | 2.08 | 2.77E-05 |
| A_23_P257365 | *GFI1* | up | 2.08 | 1.25E-04 |
| A_24_P14260 | *CARD8* | up | 2.07 | 4.68E-06 |
| A_24_P521544 |  | up | 2.07 | 4.48E-04 |
| A_23_P426511 | *C4orf21* | up | 2.07 | 7.34E-07 |
| A_24_P92472 | *CFI* | up | 2.07 | 7.45E-05 |
| A_23_P93677 | *C7orf42* | up | 2.07 | 6.72E-05 |
| A_23_P353514 | *MSL3* | up | 2.07 | 2.63E-02 |
| A_32_P1614 |  | up | 2.07 | 7.80E-08 |
| A_24_P16950 | *ZNF160* | up | 2.07 | 2.68E-02 |
| A_24_P178333 |  | up | 2.07 | 1.97E-08 |
| A_23_P7866 | *GPR115* | up | 2.07 | 3.23E-05 |
| A_24_P126682 | *SMN2* | up | 2.07 | 1.02E-03 |
| A_23_P320878 | *FAM119B* | up | 2.07 | 8.07E-07 |
| A_24_P318544 | *CSNK1D* | up | 2.07 | 2.55E-07 |
| A_24_P174550 | *RHOA* | up | 2.07 | 2.27E-03 |
| A_23_P309246 | *ZNF498* | up | 2.07 | 4.59E-08 |
| A_24_P931476 | *BOD1L* | up | 2.07 | 5.87E-06 |
| A_32_P158253 |  | up | 2.06 | 8.31E-05 |
| A_23_P47818 | *CS* | up | 2.06 | 2.20E-06 |
| A_32_P89997 |  | up | 2.06 | 1.44E-07 |
| A_32_P134402 | *EIF4A2* | up | 2.06 | 5.00E-05 |
| A_23_P211997 | *SEC22C* | up | 2.06 | 9.90E-09 |
| A_23_P119502 | *S1PR4* | up | 2.06 | 6.94E-07 |
| A_23_P205575 | *GPR135* | up | 2.06 | 1.37E-03 |
| A_32_P512061 | *GBAP1* | up | 2.06 | 3.62E-08 |
| A_23_P150255 | *RBM14* | up | 2.06 | 2.18E-05 |
| A_24_P20139 | *C2orf60* | up | 2.06 | 1.27E-06 |
| A_24_P179400 | *VEGFA* | up | 2.06 | 2.61E-05 |
| A_32_P145039 | *LOC145757* | up | 2.06 | 1.61E-04 |
| A_32_P29615 | *ZNF468* | up | 2.06 | 4.32E-04 |
| A_23_P50156 | *VPS4B* | up | 2.06 | 6.44E-04 |
| A_24_P49997 | *PFN3* | up | 2.06 | 6.05E-06 |
| A_23_P151586 | *TM9SF1* | up | 2.06 | 7.40E-07 |
| A_24_P252973 | *CLP1* | up | 2.06 | 1.09E-06 |
| A_32_P221076 | *ZC3H11A* | up | 2.06 | 2.16E-04 |
| A_23_P138461 | *C10orf2* | up | 2.05 | 3.63E-06 |
| A_23_P90565 | *C2orf86* | up | 2.05 | 3.17E-02 |
| A_23_P135499 | *CLIC4* | up | 2.05 | 3.14E-04 |
| A_24_P373726 | *ZNF587* | up | 2.05 | 1.23E-05 |
| A_24_P916656 | *CRCP* | up | 2.05 | 5.07E-04 |
| A_32_P190682 |  | up | 2.05 | 1.07E-04 |
| A_24_P40827 | *RAD51L3* | up | 2.05 | 8.98E-08 |
| A_32_P115446 |  | up | 2.05 | 1.49E-06 |
| A_23_P211627 | *NUP50* | up | 2.05 | 1.07E-07 |
| A_24_P290709 | *TOM1L1* | up | 2.05 | 1.44E-06 |
| A_24_P396980 | *PFN2* | up | 2.05 | 1.77E-04 |
| A_24_P214231 | *STIL* | up | 2.05 | 1.45E-05 |
| A_23_P250136 | *GRIK2* | up | 2.05 | 3.39E-03 |
| A_23_P55388 | *KLHL11* | up | 2.05 | 6.09E-06 |
| A_32_P155721 |  | up | 2.05 | 6.79E-04 |
| A_24_P333445 | *MORF4L2* | up | 2.04 | 4.45E-04 |
| A_23_P383601 | *FLJ31306* | up | 2.04 | 1.06E-07 |
| A_23_P208812 | *ZNF507* | up | 2.04 | 5.35E-07 |
| A_24_P144104 | *NCRNA00167* | up | 2.04 | 1.63E-02 |
| A_23_P25137 | *CAND1* | up | 2.04 | 1.80E-05 |
| A_24_P288685 | *IL13RA1* | up | 2.04 | 2.93E-07 |
| A_24_P135391 |  | up | 2.04 | 4.18E-05 |
| A_32_P9575 | *MRPL45* | up | 2.04 | 8.89E-08 |
| A_24_P926972 | *SENP3* | up | 2.04 | 2.03E-03 |
| A_23_P167367 | *PITX2* | up | 2.04 | 5.30E-04 |
| A_23_P352022 | *PVT1* | up | 2.04 | 6.23E-06 |
| A_23_P117635 | *ATXN3* | up | 2.04 | 2.94E-05 |
| A_23_P162211 | *MANSC1* | up | 2.04 | 4.82E-04 |
| A_32_P56392 | *RBMX* | up | 2.03 | 5.77E-03 |
| A_23_P25141 | *CAND1* | up | 2.03 | 3.51E-05 |
| A_23_P301133 | *IP6K2* | up | 2.03 | 6.24E-07 |
| A_24_P804667 | *METTL12* | up | 2.03 | 2.50E-08 |
| A_23_P36397 | *CYP27B1* | up | 2.03 | 3.05E-02 |
| A_23_P95070 |  | up | 2.03 | 4.86E-02 |
| A_23_P42065 | *TNFRSF21* | up | 2.03 | 1.54E-04 |
| A_24_P290257 |  | up | 2.03 | 2.31E-04 |
| A_23_P90223 | *ZNF585B* | up | 2.03 | 3.40E-07 |
| A_23_P255812 | *TMEM66* | up | 2.03 | 1.39E-04 |
| A_23_P346086 | *TPM3* | up | 2.03 | 2.85E-08 |
| A_23_P342131 | *CYBASC3* | up | 2.03 | 3.38E-04 |
| A_23_P18818 | *CNOT6* | up | 2.03 | 2.25E-05 |
| A_23_P134191 | *TRMT11* | up | 2.03 | 9.08E-05 |
| A_24_P246467 | *ATF2* | up | 2.03 | 6.51E-05 |
| A_32_P204258 |  | up | 2.03 | 3.08E-07 |
| A_32_P808 | *SLAIN2* | up | 2.03 | 5.76E-06 |
| A_24_P912889 | *SELS* | up | 2.03 | 1.21E-07 |
| A_32_P14187 | *TFAP2A* | up | 2.03 | 8.59E-08 |
| A_24_P391431 | *TAF9B* | up | 2.02 | 7.01E-06 |
| A_23_P357838 | *SDR42E1* | up | 2.02 | 2.80E-07 |
| A_32_P193166 |  | up | 2.02 | 8.24E-06 |
| A_24_P410797 | *KALRN* | up | 2.02 | 8.26E-07 |
| A_23_P214908 | *MTHFD1L* | up | 2.02 | 9.05E-06 |
| A_23_P105646 | *ATF1* | up | 2.02 | 1.88E-05 |
| A_23_P30634 | *BACH2* | up | 2.02 | 1.94E-03 |
| A_23_P92300 | *ZNF501* | up | 2.02 | 1.34E-05 |
| A_23_P142173 | *DHX34* | up | 2.02 | 2.04E-04 |
| A_23_P65555 | *SAV1* | up | 2.02 | 6.91E-07 |
| A_24_P370096 | *ZNF230* | up | 2.02 | 1.54E-04 |
| A_23_P302681 | *FIGNL1* | up | 2.02 | 1.70E-03 |
| A_23_P39263 | *ZNF57* | up | 2.02 | 2.30E-07 |
| A_23_P65609 | *GTF2A1* | up | 2.02 | 1.23E-04 |
| A_24_P945215 | *USP6NL* | up | 2.01 | 6.68E-04 |
| A_23_P100011 | *AP3S2* | up | 2.01 | 2.58E-02 |
| A_24_P128291 | *ZNF138* | up | 2.01 | 1.06E-05 |
| A_23_P96325 | *ERCC6L* | up | 2.01 | 3.30E-07 |
| A_32_P182473 | *ZNF625* | up | 2.01 | 2.41E-05 |
| A_24_P278839 | *RNF34* | up | 2.01 | 4.66E-04 |
| A_23_P157521 | *FUT10* | up | 2.01 | 5.23E-03 |
| A_32_P233250 |  | up | 2.01 | 1.30E-06 |
| A_24_P344537 | *ZNF625* | up | 2.01 | 2.85E-03 |
| A_23_P110712 | *DUSP1* | up | 2.01 | 2.18E-05 |
| A_32_P12142 |  | up | 2.01 | 4.04E-06 |
| A_32_P5376 | *NUDT16P1* | up | 2.01 | 6.28E-04 |
| A_23_P18887 | *MCCC2* | up | 2.01 | 8.56E-06 |
| A_24_P2338 | *XPNPEP3* | up | 2.01 | 6.46E-03 |
| A_23_P25433 | *C12orf4* | up | 2.01 | 9.82E-07 |
| A_23_P203075 | *USP28* | up | 2.01 | 2.18E-07 |
| A_24_P846755 |  | up | 2.01 | 3.77E-05 |
| A_23_P317347 | *ESCO1* | up | 2.01 | 8.93E-06 |
| A_24_P4334 | *RNF38* | up | 2.01 | 5.10E-06 |
| A_24_P35169 | *GATAD1* | up | 2.01 | 7.13E-09 |
| A_24_P910030 | *LOC100289550* | up | 2.01 | 7.13E-09 |
| A_24_P204484 |  | up | 2.00 | 1.96E-04 |
| A_32_P119248 | *FOXD4* | up | 2.00 | 4.05E-06 |
| A_24_P566932 | *NBPF1* | up | 2.00 | 8.37E-04 |
| A_23_P86424 | *NCOA4* | up | 2.00 | 1.49E-06 |
| A_23_P141894 | *PVR* | up | 2.00 | 1.90E-04 |
| A_24_P941773 | *METTL7A* | up | 2.00 | 9.34E-04 |
| A_24_P348892 | *C15orf57* | up | 2.00 | 1.02E-06 |
| A_24_P221445 | *OXR1* | up | 2.00 | 7.09E-05 |
| A_24_P725630 | *RNPS1* | up | 2.00 | 5.62E-05 |
| A_32_P135558 | *TMEM188* | up | 2.00 | 2.31E-04 |
