# Supplementary material for: A dedicated microarray for in-depth analysis of pre-mRNA splicing events: application to the study of genes involved in the response to targeted anticancer therapies
Source: Mol Cancer. 2014 Jan 15;13:9. doi: 10.1186/1476-4598-13-9 (PMC3899606; doi:10.1186/1476-4598-13-9)
Supplement: Additional file 3: Table S2 — Regulation of the 16 selected genes on the 44k AgilentTM microarray in SRSF2-over-expressing H358 cells. The results for the 8 deregulated genes in SRSF2-over-expressing H358 lung cancer cells in comparison to H358 control cells on the 44k microarrays are shown (≥ 1.1 FC, P-value ≤ 0.05 by t-test with FDR). The 44k microarray results for the 8 deregulated genes showed a good concordance with the 15k custom microarray results. [file 1476-4598-13-9-S3.doc]

**Supplementary Table 2.** **Regulation of the 16 selected genes on the 44k Agilent^TM^ microarray in SRSF2-over-expressing H358 cells.** The results for the 8 deregulated genes in SRSF2-over-expressing H358 lung cancer cells in comparison to H358 control cells on the 44k microarrays are shown (≥ 1.1 FC, P-value ≤ 0.05 by *t*-test with FDR). The 44k microarray results for the 8 deregulated genes showed a good concordance with the 15k custom microarray results.

| Agilent Probe Name | Gene Symbol | Regulation | Fold-Change | P-Value |
| --- | --- | --- | --- | --- |
| A_24_P179400 | *VEGFA* | up | 2.06 | 2.61E-05 |
| A_23_P81805 | *VEGFA* | up | 1.78 | 2.98E-07 |
| A_23_P70398 | *VEGFA* | up | 1.45 | 3.92E-03 |
| A_23_P215790 | *HER1/EGFR* | down | 1.83 | 4.67E-06 |
| A_23_P349416 | *HER3* | down | 1.74 | 1.87E-06 |
| A_23_P137035 | *PIR* | down | 1.23 | 2.93E-06 |
| A_23_P1594 | *VEGFB* | down | 1.23 | 6.02E-04 |
| A_24_P359267 | *AKT2* | down | 1.15 | 3.32E-02 |
| A_23_P167096 | *VEGFC* | down | 1.19 | 1.28E-04 |
| A_24_P29401 | *PIK3R1* | up | 1.19 | 2.46E-02 |
