# Supplementary material for: A dedicated microarray for in-depth analysis of pre-mRNA splicing events: application to the study of genes involved in the response to targeted anticancer therapies
Source: Mol Cancer. 2014 Jan 15;13:9. doi: 10.1186/1476-4598-13-9 (PMC3899606; doi:10.1186/1476-4598-13-9)
Supplement: Additional file 4: Table S3 — Labeling efficiency for hybridization of the 15k custom microarray. The labeled cRNA yield and specific activity of cyanine3 are shown for each of the three labeling experiments performed. The cRNA yield should be superior to 1.65 μg, and the specific activity superior to 9.0 pmol cyanine3 per μg cRNA. The number of 15k replicates using Quick Amp labeling was 4 for each condition (control or SRSF2 over-expression), and the number of 44k replicates was 6 (i.e. 2 for each of the three labeling) for each condition. [file 1476-4598-13-9-S4.doc]

**Supplementary Table 3.** **Labeling efficiency for hybridization of the 15k custom microarray.** The labeled cRNA yield and specific activity of cyanine3 are shown for each of the three labeling experiments performed. The cRNA yield should be superior to 1.65 μg, and the specific activity superior to 9.0 pmol cyanine3 per μg cRNA. The number of 15k replicates using Quick Amp labeling was 4 for each condition (control or SRSF2 over-expression), and the number of 44k replicates was 6 (*i.e*. 2 for each of the three labeling) for each condition.

|  | Quick Amp Labeling | 1 | 2 | 3 |
| --- | --- | --- | --- | --- |
| Control Condition | cRNA Yield (µg) | 11.34 | 10.35 | 10.38 |
|  | Specific Activity (pmol Cy3 per μg cRNA) | 17.12 | 18.26 | 17.34 |
|  | 15k Microarray Hybridization | no | no | yes |
|  | 15k Replicate Microarray | 0 | 0 | 4 |
|  | 44k Microarray Hybridization | yes | yes | yes |
|  | 44k Replicate Microarray | 2 | 2 | 2 |
| SRSF2 Over-expression Condition | cRNA Yield (µg) | 13.35 | 11.36 | 7.95 |
|  | Specific Activity (pmol Cy3 per μg cRNA) | 17.53 | 17.43 | 16.98 |
|  | 15k Microarray Hybridization | no | no | yes |
|  | 15k Replicate Microarray | 0 | 0 | 4 |
|  | 44k Microarray Hybridization | yes | yes | yes |
|  | 44k Replicate Microarray | 2 | 2 | 2 |
