# Supplementary material for: A dedicated microarray for in-depth analysis of pre-mRNA splicing events: application to the study of genes involved in the response to targeted anticancer therapies
Source: Mol Cancer. 2014 Jan 15;13:9. doi: 10.1186/1476-4598-13-9 (PMC3899606; doi:10.1186/1476-4598-13-9)
Supplement: Additional file 5: Table S4 — Comparison of the 15k custom and 44k AgilentTM microarray results. The results are shown for the AgilentTM probes present on both chips: 313 probes were deregulated in SRSF2-over-expressing H358 lung cancer cells in comparison to H358 control cells on the 15k custom chip (≥ 1.5 FC, P-value ≤ 0.05), and 310 had the same type of regulation, considering statistically relevant and not statistically relevant regulations on the 44k chip (same type of regulation = 1; other type of regulation = 0). [file 1476-4598-13-9-S5.doc]

**Supplementary Table 4.** **Comparison of the 15k custom and 44k Agilent^TM^ microarray results.** The results are shown for the Agilent^TM^ probes present on both chips: 313 probes were deregulated in SRSF2-over-expressing H358 lung cancer cells in comparison to H358 control cells on the 15k custom chip (≥ 1.5 FC, P-value ≤ 0.05), and 310 had the same type of regulation, considering statistically relevant and not statistically relevant regulations on the 44k chip (same type of regulation = 1; other type of regulation = 0).

|  |  | 15k Custom Microarray | | | 44k Commercial Microarray | | | Comparison 15k *vs*. 44k |
| --- | --- | --- | --- | --- | --- | --- | --- | --- |
| Agilent Probe Name | Gene Symbol | Regulation | Fold-Change | P-Value | Regulation | Fold-Change | P-Value | same regulation = 1; other = 0 |
| A_23_P83751 | *C11orf63* | down | 1.94 | 1.20E-02 | up | 1.00 | 8.87E-01 | 0 |
| A_24_P711050 |  | down | 1.75 | 9.65E-04 | up | 1.01 | 9.35E-01 | 0 |
| A_24_P100387 | *GK* | down | 1.59 | 7.41E-04 | up | 1.01 | 7.55E-01 | 0 |
| A_24_P927716 | *UNQ9368* | up | 6.10 | 8.63E-08 | up | 1.11 | 8.12E-02 | 1 |
| A_24_P941947 | *LPCAT1* | up | 5.68 | 2.45E-05 | up | 1.08 | 2.19E-02 | 1 |
| A_23_P357504 |  | up | 4.87 | 2.11E-08 | up | 1.47 | 3.78E-04 | 1 |
| A_24_P927639 |  | up | 3.89 | 7.31E-07 | up | 1.43 | 5.72E-05 | 1 |
| A_23_P134980 | *FGD3* | up | 3.55 | 3.04E-03 | up | 1.30 | 1.19E-02 | 1 |
| A_23_P357248 | *ZNF343* | up | 3.24 | 5.48E-03 | up | 1.10 | 8.83E-03 | 1 |
| A_24_P268993 | *LEAP2* | up | 3.04 | 5.56E-09 | up | 1.49 | 1.51E-05 | 1 |
| A_23_P27247 | *MED13* | up | 2.87 | 3.58E-04 | up | 1.35 | 6.77E-04 | 1 |
| A_23_P49610 | *C17orf91* | up | 2.80 | 1.32E-08 | up | 1.78 | 5.79E-03 | 1 |
| A_23_P128215 | *SOCS2* | up | 2.74 | 1.19E-08 | up | 1.70 | 8.07E-04 | 1 |
| A_32_P93459 |  | up | 2.73 | 2.40E-06 | up | 1.06 | 5.53E-03 | 1 |
| A_24_P912228 | *LOC100131053* | up | 2.48 | 2.28E-07 | up | 1.14 | 7.64E-03 | 1 |
| A_24_P281243 |  | up | 2.38 | 7.55E-04 | up | 1.10 | 2.18E-03 | 1 |
| A_32_P107208 | *C1orf106* | up | 2.34 | 2.23E-04 | up | 1.04 | 2.09E-02 | 1 |
| A_23_P83234 | *ZBTB6* | up | 2.24 | 1.36E-03 | up | 1.05 | 1.61E-02 | 1 |
| A_23_P258931 | *ZNF623* | up | 2.20 | 5.50E-07 | up | 1.42 | 4.69E-03 | 1 |
| A_23_P258814 | *DPH3B* | up | 2.20 | 3.26E-07 | up | 1.60 | 1.37E-03 | 1 |
| A_23_P49459 | *LOC81691* | up | 2.19 | 2.82E-06 | up | 1.08 | 2.26E-02 | 1 |
| A_23_P134026 | *C6orf35* | up | 2.18 | 3.83E-04 | up | 1.19 | 2.11E-02 | 1 |
| A_23_P161197 | *SEC61A2* | up | 2.14 | 1.32E-07 | up | 1.56 | 2.32E-04 | 1 |
| A_23_P404595 | *ZNF417* | up | 2.14 | 9.54E-05 | up | 1.02 | 2.71E-02 | 1 |
| A_23_P166193 | *EDEM2* | up | 2.09 | 1.82E-06 | up | 1.20 | 1.26E-02 | 1 |
| A_32_P145639 |  | up | 2.07 | 1.51E-05 | up | 1.09 | 7.68E-03 | 1 |
| A_24_P205120 | *SPG7* | up | 2.04 | 6.35E-07 | up | 1.18 | 2.70E-03 | 1 |
| A_23_P427634 | *ASB10* | up | 2.01 | 2.99E-04 | up | 1.02 | 2.63E-01 | 1 |
| A_23_P111188 | *ZBTB22* | up | 1.98 | 2.14E-02 | up | 1.17 | 1.47E-02 | 1 |
| A_23_P65068 | *EID3* | up | 1.97 | 3.84E-06 | up | 1.06 | 4.16E-02 | 1 |
| A_24_P931476 | *BOD1L* | up | 1.95 | 9.93E-07 | up | 1.67 | 1.85E-04 | 1 |
| A_23_P327907 | *C8orf37* | up | 1.94 | 4.43E-02 | up | 1.11 | 3.70E-02 | 1 |
| A_23_P318208 | *AP1G1* | up | 1.92 | 6.10E-07 | up | 1.41 | 2.00E-03 | 1 |
| A_23_P27758 |  | up | 1.88 | 9.09E-08 | up | 1.32 | 7.87E-03 | 1 |
| A_23_P27256 | *TWSG1* | up | 1.88 | 5.65E-08 | up | 1.42 | 4.76E-04 | 1 |
| A_23_P49521 | *SFRS1* | up | 1.85 | 9.60E-07 | up | 1.39 | 1.20E-02 | 1 |
| A_32_P75399 |  | up | 1.84 | 2.21E-04 | up | 1.05 | 4.43E-02 | 1 |
| A_24_P214231 | *STIL* | up | 1.84 | 9.61E-07 | up | 1.10 | 1.35E-03 | 1 |
| A_24_P288685 | *IL13RA1* | up | 1.83 | 1.60E-08 | up | 1.31 | 1.44E-04 | 1 |
| A_24_P204971 | *HBP1* | up | 1.83 | 1.73E-05 | up | 1.25 | 2.70E-02 | 1 |
| A_24_P100228 | *XBP1* | up | 1.82 | 1.00E-06 | up | 1.24 | 2.79E-03 | 1 |
| A_23_P134517 | *PURB* | up | 1.81 | 2.35E-08 | up | 1.70 | 1.44E-03 | 1 |
| A_23_P138819 | *MRPL49* | up | 1.80 | 1.87E-06 | up | 1.23 | 1.34E-04 | 1 |
| A_23_P370005 | *RIPK1* | up | 1.77 | 3.05E-02 | up | 1.23 | 3.69E-04 | 1 |
| A_32_P6972 |  | up | 1.75 | 1.51E-07 | up | 1.57 | 7.15E-03 | 1 |
| A_23_P88331 | *DLGAP5* | up | 1.74 | 2.27E-08 | up | 1.72 | 4.04E-03 | 1 |
| A_23_P27285 | *MPPE1* | up | 1.74 | 2.38E-02 | up | 1.43 | 7.43E-03 | 1 |
| A_23_P311732 | *GNG12* | up | 1.73 | 1.16E-07 | up | 1.28 | 1.97E-04 | 1 |
| A_24_P300483 | *C7orf25* | up | 1.72 | 2.44E-06 | up | 1.17 | 5.03E-03 | 1 |
| A_24_P130792 | *NSUN4* | up | 1.71 | 4.10E-05 | up | 1.37 | 4.58E-03 | 1 |
| A_24_P467073 | *hCG_1643808* | up | 1.71 | 2.29E-05 | up | 1.13 | 3.49E-03 | 1 |
| A_23_P88021 | *GPR180* | up | 1.70 | 1.76E-08 | up | 1.28 | 1.12E-02 | 1 |
| A_24_P346604 | *UBE2W* | up | 1.70 | 2.31E-05 | up | 1.18 | 2.81E-02 | 1 |
| A_24_P931364 |  | up | 1.69 | 2.14E-05 | up | 1.04 | 3.22E-02 | 1 |
| A_23_P106194 | *FOS* | up | 1.68 | 6.86E-07 | up | 1.53 | 3.64E-03 | 1 |
| A_23_P216068 | *ATAD2* | up | 1.68 | 1.36E-07 | up | 2.01 | 1.05E-01 | 1 |
| A_23_P256956 | *KIF20A* | up | 1.68 | 3.81E-04 | up | 1.84 | 2.33E-02 | 1 |
| A_32_P54018 | *KIAA1143* | up | 1.68 | 3.55E-06 | up | 1.37 | 3.41E-03 | 1 |
| A_23_P153128 | *C18orf2* | up | 1.67 | 1.34E-07 | up | 1.17 | 1.03E-02 | 1 |
| A_24_P27513 | *CNTNAP4* | up | 1.67 | 9.32E-09 | up | 1.21 | 1.71E-03 | 1 |
| A_24_P814096 |  | up | 1.66 | 3.64E-07 | up | 1.55 | 1.92E-02 | 1 |
| A_23_P395075 | *KDM3A* | up | 1.65 | 1.21E-06 | up | 1.19 | 8.08E-03 | 1 |
| A_32_P46510 | *ZC3H6* | up | 1.65 | 1.12E-06 | up | 1.25 | 4.13E-03 | 1 |
| A_23_P27636 | *ZNF700* | up | 1.65 | 3.61E-04 | up | 1.04 | 3.71E-02 | 1 |
| A_23_P336644 | *TOR1AIP2* | up | 1.64 | 9.54E-05 | up | 1.12 | 3.56E-03 | 1 |
| A_23_P111865 | *ZSCAN21* | up | 1.64 | 2.73E-06 | up | 1.15 | 3.10E-03 | 1 |
| A_23_P336554 | *IL1RAP* | up | 1.63 | 4.52E-02 | up | 1.06 | 1.72E-01 | 1 |
| A_23_P88347 | *FERMT2* | up | 1.62 | 4.73E-07 | up | 1.18 | 2.58E-03 | 1 |
| A_24_P205364 | *SHMT1* | up | 1.62 | 3.43E-06 | up | 1.34 | 3.21E-04 | 1 |
| A_23_P83175 | *PTPLAD2* | up | 1.59 | 5.62E-07 | up | 1.23 | 9.88E-03 | 1 |
| A_23_P111888 | *CTHRC1* | up | 1.58 | 1.54E-05 | up | 1.56 | 6.70E-03 | 1 |
| A_23_P111306 | *RBM16* | up | 1.57 | 4.54E-07 | up | 1.16 | 6.27E-03 | 1 |
| A_23_P118135 |  | up | 1.57 | 9.16E-07 | up | 1.23 | 7.28E-03 | 1 |
| A_32_P38003 | *EIF2AK2* | up | 1.57 | 5.24E-03 | up | 1.06 | 2.01E-03 | 1 |
| A_23_P88303 | *HSPA2* | up | 1.56 | 3.79E-07 | up | 1.47 | 3.13E-03 | 1 |
| A_32_P54531 | *ZNF826* | up | 1.56 | 6.39E-04 | up | 1.31 | 7.08E-02 | 1 |
| A_23_P27404 | *ZNF841* | up | 1.56 | 8.00E-07 | up | 1.20 | 1.35E-03 | 1 |
| A_23_P311740 | *CUL9* | up | 1.55 | 1.06E-03 | up | 1.45 | 8.09E-03 | 1 |
| A_23_P33683 | *MARCH2* | up | 1.55 | 3.46E-03 | up | 1.03 | 1.19E-01 | 1 |
| A_23_P427622 | *UNQ1887* | up | 1.53 | 2.31E-05 | up | 1.20 | 3.08E-03 | 1 |
| A_23_P153197 | *TGIF1* | up | 1.52 | 2.08E-06 | up | 1.50 | 1.07E-01 | 1 |
| A_23_P312851 | *SILV* | up | 1.51 | 2.47E-06 | up | 1.07 | 1.88E-02 | 1 |
| A_23_P98722 | *CAPRIN1* | up | 1.51 | 1.96E-09 | up | 1.59 | 1.42E-04 | 1 |
| A_23_P404129 | *SPATA5* | up | 1.51 | 1.28E-03 | up | 1.09 | 2.73E-02 | 1 |
| A_23_P11507 | *ZZZ3* | up | 1.50 | 1.74E-03 | up | 1.33 | 2.37E-03 | 1 |
| A_24_P163405 | *TRIM27* | up | 1.50 | 1.16E-07 | up | 1.64 | 1.83E-03 | 1 |
| A_24_P272310 | *MUSTN1* | down | 10.12 | 3.54E-03 | down | 1.07 | 2.88E-03 | 1 |
| A_23_P59967 | *RP1* | down | 6.34 | 2.00E-02 | down | 1.05 | 2.04E-02 | 1 |
| A_23_P1331 | *COL13A1* | down | 4.89 | 1.59E-09 | down | 1.70 | 4.65E-03 | 1 |
| A_32_P104746 | *ZFYVE28* | down | 4.63 | 1.47E-02 | down | 1.04 | 4.44E-02 | 1 |
| A_24_P941427 |  | down | 4.23 | 2.88E-03 | down | 1.02 | 1.96E-02 | 1 |
| A_23_P347128 |  | down | 3.85 | 7.38E-07 | down | 1.28 | 1.63E-04 | 1 |
| A_23_P83599 | *PRKAR1B* | down | 3.57 | 4.92E-07 | down | 1.18 | 1.43E-03 | 1 |
| A_32_P54616 | *KIAA1407* | down | 3.54 | 9.59E-05 | down | 1.16 | 8.43E-03 | 1 |
| A_24_P231057 | *BOD1L* | down | 3.42 | 1.12E-03 | down | 1.05 | 1.72E-03 | 1 |
| A_32_P75772 |  | down | 3.40 | 7.17E-09 | down | 1.28 | 2.28E-03 | 1 |
| A_23_P88849 | *RRAD* | down | 3.32 | 3.00E-09 | down | 2.51 | 2.54E-03 | 1 |
| A_24_P205627 | *TXLNA* | down | 3.26 | 4.03E-06 | down | 1.07 | 2.34E-02 | 1 |
| A_32_P122268 | *SYT15* | down | 3.04 | 4.63E-08 | down | 1.22 | 1.12E-03 | 1 |
| A_32_P96124 |  | down | 2.92 | 3.27E-02 | down | 1.11 | 4.13E-03 | 1 |
| A_23_P123193 | *ACTR3B* | down | 2.90 | 7.01E-10 | down | 1.87 | 3.95E-03 | 1 |
| A_24_P873764 | *BCR* | down | 2.89 | 1.22E-08 | down | 2.15 | 6.16E-05 | 1 |
| A_32_P111019 |  | down | 2.89 | 1.71E-06 | down | 1.05 | 1.02E-02 | 1 |
| A_24_P243044 | *GSK3B* | down | 2.87 | 7.76E-05 | down | 1.08 | 1.90E-02 | 1 |
| A_23_P98248 | *TRPT1* | down | 2.87 | 1.69E-10 | down | 2.22 | 8.04E-04 | 1 |
| A_23_P304991 | *HLCS* | down | 2.86 | 5.95E-08 | down | 1.43 | 3.27E-03 | 1 |
| A_23_P153549 | *GRIN2D* | down | 2.77 | 2.52E-03 | down | 1.51 | 2.01E-02 | 1 |
| A_23_P97283 | *PAQR6* | down | 2.76 | 8.25E-05 | down | 1.51 | 1.31E-03 | 1 |
| A_24_P927090 |  | down | 2.72 | 9.16E-03 | down | 1.02 | 7.88E-02 | 1 |
| A_23_P119794 |  | down | 2.72 | 1.44E-09 | down | 1.72 | 3.47E-03 | 1 |
| A_23_P134477 | *C7orf50* | down | 2.66 | 2.32E-05 | down | 2.12 | 3.16E-03 | 1 |
| A_23_P129801 | *RAB40B* | down | 2.61 | 2.37E-07 | down | 1.22 | 4.38E-03 | 1 |
| A_24_P504294 |  | down | 2.60 | 2.08E-02 | down | 1.03 | 2.47E-01 | 1 |
| A_24_P361457 | *FLJ35220* | down | 2.56 | 5.27E-09 | down | 1.14 | 1.51E-02 | 1 |
| A_24_P80571 |  | down | 2.52 | 5.96E-09 | down | 1.18 | 1.16E-02 | 1 |
| A_23_P93818 | *STAG3L1* | down | 2.51 | 9.57E-08 | down | 1.61 | 1.45E-03 | 1 |
| A_32_P96692 | *POLH* | down | 2.51 | 1.05E-09 | down | 1.42 | 7.71E-04 | 1 |
| A_24_P288979 | *GNPTAB* | down | 2.49 | 4.68E-03 | down | 1.08 | 2.49E-03 | 1 |
| A_23_P395374 | *HIST1H4D* | down | 2.49 | 3.78E-07 | down | 2.02 | 1.85E-03 | 1 |
| A_24_P941376 | *ZNF473* | down | 2.47 | 2.06E-05 | down | 1.14 | 1.47E-02 | 1 |
| A_23_P99285 | *GPR19* | down | 2.46 | 2.08E-02 | down | 1.04 | 6.21E-02 | 1 |
| A_32_P192842 | *LOC100288985* | down | 2.46 | 1.20E-03 | down | 1.06 | 5.12E-03 | 1 |
| A_23_P88309 | *SLC38A6* | down | 2.45 | 1.08E-08 | down | 1.39 | 2.02E-03 | 1 |
| A_23_P153524 | *C19orf73* | down | 2.43 | 3.21E-04 | down | 1.15 | 6.56E-03 | 1 |
| A_23_P153867 | *LASS4* | down | 2.40 | 6.80E-03 | down | 1.04 | 2.26E-02 | 1 |
| A_24_P368544 | *SLC25A26* | down | 2.40 | 5.90E-03 | down | 1.08 | 2.07E-01 | 1 |
| A_23_P124202 | *ING5* | down | 2.32 | 4.54E-02 | down | 1.05 | 2.63E-02 | 1 |
| A_23_P27734 | *NPAS1* | down | 2.32 | 5.85E-04 | down | 1.03 | 3.04E-02 | 1 |
| A_32_P200165 | *ELAVL1* | down | 2.30 | 4.79E-06 | down | 1.13 | 2.57E-02 | 1 |
| A_32_P93852 | *BOD1* | down | 2.29 | 2.05E-08 | down | 1.57 | 4.39E-03 | 1 |
| A_24_P205427 |  | down | 2.27 | 4.32E-08 | down | 1.35 | 9.66E-03 | 1 |
| A_23_P49220 | *TCF25* | down | 2.24 | 8.20E-09 | down | 1.85 | 1.61E-03 | 1 |
| A_23_P88083 | *CDC16* | down | 2.24 | 4.25E-05 | down | 1.65 | 2.16E-03 | 1 |
| A_23_P118306 | *DNAJA3* | down | 2.22 | 1.00E-08 | down | 1.89 | 4.59E-04 | 1 |
| A_32_P104448 |  | down | 2.21 | 2.06E-03 | down | 1.04 | 2.00E-03 | 1 |
| A_23_P32029 | *SLC35D2* | down | 2.19 | 3.76E-07 | down | 1.13 | 2.86E-03 | 1 |
| A_32_P462013 |  | down | 2.19 | 2.21E-02 | down | 1.03 | 5.66E-02 | 1 |
| A_24_P142531 | *DDX42* | down | 2.18 | 8.33E-05 | down | 1.11 | 3.67E-03 | 1 |
| A_24_P205316 | *DNAJC5* | down | 2.18 | 3.73E-08 | down | 1.53 | 4.12E-03 | 1 |
| A_23_P123125 | *CCM2* | down | 2.17 | 6.29E-08 | down | 1.74 | 3.18E-04 | 1 |
| A_23_P134835 | *CSGALNACT1* | down | 2.16 | 1.29E-02 | down | 1.04 | 2.38E-02 | 1 |
| A_32_P54594 |  | down | 2.16 | 1.02E-07 | down | 1.16 | 4.21E-03 | 1 |
| A_24_P346181 | *FAM120B* | down | 2.15 | 1.38E-03 | down | 1.04 | 4.13E-02 | 1 |
| A_23_P7582 | *TCF7* | down | 2.14 | 4.67E-07 | down | 1.16 | 2.29E-01 | 1 |
| A_23_P434919 | *RAB42* | down | 2.14 | 2.02E-03 | down | 1.03 | 1.09E-01 | 1 |
| A_24_P205130 | *FNBP1* | down | 2.11 | 2.09E-02 | down | 1.23 | 2.19E-02 | 1 |
| A_24_P100535 | *SYT15* | down | 2.10 | 2.12E-08 | down | 1.21 | 1.01E-03 | 1 |
| A_24_P185044 | *MLKL* | down | 2.10 | 1.06E-07 | down | 1.10 | 3.41E-01 | 1 |
| A_23_P357104 | *ANXA6* | down | 2.09 | 1.85E-05 | down | 1.07 | 1.56E-02 | 1 |
| A_23_P108823 | *OSBPL6* | down | 2.09 | 1.03E-07 | down | 1.34 | 3.93E-03 | 1 |
| A_23_P119006 |  | down | 2.08 | 4.42E-04 | down | 1.04 | 8.66E-03 | 1 |
| A_24_P100605 | *RALGAPA2* | down | 2.07 | 1.81E-05 | down | 1.06 | 1.39E-02 | 1 |
| A_24_P945283 | *DLG3* | down | 2.06 | 1.40E-08 | down | 1.75 | 4.21E-05 | 1 |
| A_23_P88848 | *DUS2L* | down | 2.06 | 1.78E-06 | down | 1.36 | 6.93E-03 | 1 |
| A_24_P825874 | *POTEE* | down | 2.06 | 6.35E-04 | down | 1.08 | 3.53E-02 | 1 |
| A_23_P395954 | *SSH2* | down | 2.05 | 8.98E-03 | down | 1.02 | 2.65E-02 | 1 |
| A_32_P192545 | *TCEAL6* | down | 2.04 | 5.89E-09 | down | 1.40 | 7.00E-03 | 1 |
| A_23_P134014 | *ADAT2* | down | 2.04 | 2.08E-05 | down | 1.16 | 6.81E-03 | 1 |
| A_23_P75369 | *TCIRG1* | down | 2.03 | 1.14E-07 | down | 1.84 | 4.61E-04 | 1 |
| A_23_P40611 | *TCN2* | down | 2.03 | 4.44E-05 | down | 1.02 | 1.68E-01 | 1 |
| A_23_P153692 | *XRCC1* | down | 2.02 | 3.65E-07 | down | 1.76 | 5.04E-03 | 1 |
| A_24_P346431 | *TNS3* | down | 2.00 | 6.27E-08 | down | 1.44 | 7.52E-04 | 1 |
| A_23_P49310 | *ERN2* | down | 2.00 | 2.42E-03 | down | 1.18 | 4.48E-02 | 1 |
| A_24_P300777 | *ADAM8* | down | 2.00 | 5.97E-07 | down | 1.79 | 1.25E-05 | 1 |
| A_23_P68628 | *NECAB3* | down | 1.99 | 2.65E-08 | down | 1.23 | 1.03E-04 | 1 |
| A_23_P68121 | *PSD4* | down | 1.98 | 1.31E-07 | down | 1.17 | 1.42E-04 | 1 |
| A_23_P32054 | *CAMSAP1* | down | 1.98 | 3.97E-04 | down | 1.03 | 6.57E-02 | 1 |
| A_32_P200429 |  | down | 1.97 | 3.67E-08 | down | 1.48 | 8.64E-03 | 1 |
| A_23_P98580 | *FADS2* | down | 1.97 | 6.69E-07 | down | 1.47 | 3.80E-03 | 1 |
| A_23_P32021 | *FANCC* | down | 1.96 | 4.00E-08 | down | 1.44 | 4.37E-03 | 1 |
| A_23_P134946 | *LRRC14* | down | 1.96 | 2.37E-06 | down | 1.46 | 1.79E-02 | 1 |
| A_32_P357301 | *PPHLN1* | down | 1.95 | 1.36E-04 | down | 1.02 | 1.97E-01 | 1 |
| A_24_P878992 |  | down | 1.95 | 4.35E-05 | down | 1.18 | 3.23E-02 | 1 |
| A_24_P153800 | *SUPT16H* | down | 1.95 | 1.49E-07 | down | 1.28 | 1.79E-01 | 1 |
| A_23_P68031 | *STAT4* | down | 1.95 | 4.79E-10 | down | 1.14 | 5.08E-05 | 1 |
| A_24_P142495 | *KRTAP1-3* | down | 1.94 | 1.84E-05 | down | 1.59 | 2.78E-03 | 1 |
| A_23_P111860 | *RADIL* | down | 1.93 | 1.95E-08 | down | 1.43 | 4.46E-04 | 1 |
| A_23_P388914 | *CCDC101* | down | 1.93 | 1.02E-02 | down | 1.05 | 7.62E-02 | 1 |
| A_24_P100351 | *GNL3L* | down | 1.93 | 1.28E-08 | down | 1.75 | 5.62E-05 | 1 |
| A_24_P732565 |  | down | 1.92 | 3.81E-02 | down | 1.08 | 1.21E-03 | 1 |
| A_24_P757255 | *GPR157* | down | 1.92 | 9.57E-04 | down | 1.15 | 1.69E-03 | 1 |
| A_23_P336796 | *GXYLT1* | down | 1.91 | 5.68E-05 | down | 1.03 | 1.82E-01 | 1 |
| A_24_P770494 |  | down | 1.91 | 2.95E-04 | down | 1.70 | 1.26E-02 | 1 |
| A_32_P425998 | *IGFN1* | down | 1.91 | 3.04E-03 | down | 1.02 | 2.24E-01 | 1 |
| A_24_P912720 | *CHRNA4* | down | 1.91 | 1.98E-04 | down | 1.45 | 6.50E-03 | 1 |
| A_23_P32036 | *C9orf95* | down | 1.90 | 3.82E-08 | down | 1.32 | 1.02E-02 | 1 |
| A_23_P27493 | *PPAN* | down | 1.90 | 4.57E-02 | down | 1.71 | 1.69E-03 | 1 |
| A_23_P111621 | *GTF2IRD1* | down | 1.89 | 4.57E-07 | down | 1.58 | 3.75E-03 | 1 |
| A_23_P111194 | *SPDEF* | down | 1.89 | 1.60E-05 | down | 1.35 | 1.34E-02 | 1 |
| A_24_P929137 |  | down | 1.88 | 2.16E-03 | down | 1.05 | 1.79E-02 | 1 |
| A_23_P91827 | *MINA* | down | 1.88 | 1.70E-08 | down | 1.24 | 1.69E-02 | 1 |
| A_24_P281580 | *GATS* | down | 1.88 | 3.52E-05 | down | 1.05 | 6.90E-02 | 1 |
| A_23_P97328 | *C1orf35* | down | 1.87 | 2.22E-08 | down | 1.71 | 6.68E-03 | 1 |
| A_23_P98455 | *VWA5A* | down | 1.87 | 1.20E-02 | down | 1.01 | 2.85E-01 | 1 |
| A_24_P918147 |  | down | 1.87 | 1.88E-06 | down | 1.15 | 2.33E-03 | 1 |
| A_32_P210691 |  | down | 1.87 | 2.15E-05 | down | 1.15 | 9.08E-03 | 1 |
| A_23_P153651 | *FSD1* | down | 1.86 | 1.98E-03 | down | 1.16 | 3.66E-03 | 1 |
| A_23_P67042 | *MOCOS* | down | 1.86 | 1.76E-04 | down | 1.40 | 5.35E-03 | 1 |
| A_23_P134419 | *ZP3* | down | 1.86 | 3.42E-04 | down | 1.11 | 1.06E-03 | 1 |
| A_24_P205589 | *ACOT7* | down | 1.85 | 4.13E-07 | down | 1.52 | 1.29E-02 | 1 |
| A_32_P80901 | *RPS15A* | down | 1.85 | 5.57E-04 | down | 1.22 | 1.12E-02 | 1 |
| A_23_P317800 | *ANAPC4* | down | 1.84 | 8.00E-08 | down | 1.59 | 4.96E-03 | 1 |
| A_23_P404094 | *FLJ39582* | down | 1.84 | 9.82E-03 | down | 1.03 | 4.54E-02 | 1 |
| A_23_P395524 | *PWP1* | down | 1.84 | 4.43E-07 | down | 1.67 | 3.34E-03 | 1 |
| A_23_P27677 | *IRF3* | down | 1.84 | 3.51E-07 | down | 1.41 | 1.16E-02 | 1 |
| A_23_P427075 | *CTNS* | down | 1.84 | 5.20E-08 | down | 1.17 | 5.18E-03 | 1 |
| A_24_P753454 | *LOC100132273* | down | 1.83 | 1.21E-04 | down | 1.06 | 4.39E-02 | 1 |
| A_23_P118086 | *SPATA2L* | down | 1.83 | 2.50E-03 | down | 1.19 | 6.05E-02 | 1 |
| A_32_P96933 |  | down | 1.82 | 9.32E-03 | down | 1.12 | 9.77E-02 | 1 |
| A_23_P501961 | *L3MBTL* | down | 1.82 | 1.64E-07 | down | 1.19 | 2.06E-03 | 1 |
| A_23_P59999 |  | down | 1.82 | 9.98E-04 | down | 1.05 | 6.67E-03 | 1 |
| A_24_P256552 | *CSTF3* | down | 1.81 | 6.25E-04 | down | 1.19 | 2.64E-03 | 1 |
| A_24_P158385 | *ZMYND19* | down | 1.80 | 7.18E-07 | down | 1.34 | 7.46E-04 | 1 |
| A_23_P404005 | *FAM58A* | down | 1.79 | 1.16E-06 | down | 1.54 | 1.72E-03 | 1 |
| A_24_P158314 | *GARNL3* | down | 1.79 | 4.30E-05 | down | 1.09 | 1.25E-03 | 1 |
| A_24_P256674 | *ARHGEF10* | down | 1.79 | 3.26E-03 | down | 1.08 | 4.31E-02 | 1 |
| A_24_P205263 | *ESF1* | down | 1.79 | 3.12E-08 | down | 1.30 | 2.61E-03 | 1 |
| A_24_P163113 | *CDV3* | down | 1.79 | 1.67E-06 | down | 1.51 | 3.69E-03 | 1 |
| A_24_P330366 | *RCOR1* | down | 1.77 | 1.30E-06 | down | 1.33 | 3.91E-03 | 1 |
| A_23_P404730 | *ARHGAP33* | down | 1.77 | 2.77E-02 | down | 1.03 | 9.14E-02 | 1 |
| A_24_P583350 | *ALG1L* | down | 1.76 | 1.97E-06 | down | 1.42 | 4.23E-03 | 1 |
| A_23_P97573 | *PRPF3* | down | 1.76 | 2.62E-06 | down | 1.23 | 5.03E-04 | 1 |
| A_23_P106162 | *C14orf106* | down | 1.76 | 8.79E-08 | down | 1.20 | 6.59E-03 | 1 |
| A_23_P386942 | *DIRAS1* | down | 1.75 | 2.30E-07 | down | 1.09 | 8.07E-03 | 1 |
| A_24_P941988 | *SPAST* | down | 1.74 | 1.67E-02 | down | 1.07 | 1.27E-01 | 1 |
| A_24_P125096 | *MT1X* | down | 1.74 | 5.37E-04 | down | 1.70 | 2.23E-02 | 1 |
| A_24_P205019 | *ZNF250* | down | 1.74 | 6.52E-08 | down | 1.20 | 7.41E-03 | 1 |
| A_23_P68665 | *ADRM1* | down | 1.74 | 1.70E-05 | down | 1.49 | 7.60E-03 | 1 |
| A_23_P49539 | *BAHCC1* | down | 1.74 | 1.96E-06 | down | 1.11 | 4.08E-02 | 1 |
| A_23_P357147 | *WWP2* | down | 1.73 | 5.33E-08 | down | 1.10 | 1.45E-02 | 1 |
| A_23_P27167 | *RNASEH1* | down | 1.72 | 3.82E-06 | down | 1.42 | 1.34E-02 | 1 |
| A_23_P118174 | *PLK1* | down | 1.72 | 1.04E-06 | down | 1.37 | 8.93E-03 | 1 |
| A_24_P142151 | *LRRC28* | down | 1.72 | 2.17E-02 | down | 1.02 | 7.25E-01 | 1 |
| A_23_P111273 | *TBC1D7* | down | 1.72 | 5.48E-07 | down | 1.53 | 2.52E-03 | 1 |
| A_32_P200934 |  | down | 1.72 | 1.98E-05 | down | 1.14 | 6.30E-02 | 1 |
| A_24_P92256 | *SGSM2* | down | 1.71 | 1.22E-06 | down | 1.27 | 9.62E-03 | 1 |
| A_23_P27827 | *ZBTB7A* | down | 1.71 | 3.32E-06 | down | 1.25 | 1.29E-02 | 1 |
| A_23_P370035 | *DCAF4* | down | 1.70 | 9.80E-03 | down | 1.26 | 5.37E-02 | 1 |
| A_23_P118774 | *KPNB1* | down | 1.70 | 8.38E-03 | down | 1.43 | 1.08E-02 | 1 |
| A_24_P920880 | *ZSCAN18* | down | 1.69 | 6.26E-05 | down | 1.07 | 2.20E-02 | 1 |
| A_23_P83110 | *CDK5RAP2* | down | 1.69 | 1.86E-08 | down | 1.30 | 3.65E-03 | 1 |
| A_23_P99226 | *SIRT4* | down | 1.69 | 6.28E-03 | down | 1.01 | 3.80E-01 | 1 |
| A_23_P357546 | *PNKD* | down | 1.69 | 3.63E-05 | down | 1.11 | 2.31E-02 | 1 |
| A_23_P258978 | *GOLGA1* | down | 1.69 | 1.23E-04 | down | 1.07 | 9.12E-03 | 1 |
| A_32_P44210 |  | down | 1.69 | 1.28E-02 | down | 1.01 | 1.11E-01 | 1 |
| A_24_P330030 | *ORAOV1* | down | 1.68 | 2.34E-06 | down | 1.07 | 5.46E-03 | 1 |
| A_23_P327013 | *KIAA0146* | down | 1.68 | 1.63E-06 | down | 1.15 | 1.44E-01 | 1 |
| A_23_P118427 | *MAP2K3* | down | 1.68 | 7.79E-05 | down | 1.25 | 4.54E-03 | 1 |
| A_24_P100190 | *C21orf93* | down | 1.67 | 2.37E-04 | down | 1.06 | 1.86E-01 | 1 |
| A_32_P181271 |  | down | 1.67 | 7.83E-04 | down | 1.07 | 6.17E-02 | 1 |
| A_23_P153441 | *HOOK2* | down | 1.67 | 1.22E-06 | down | 1.33 | 8.16E-03 | 1 |
| A_23_P98304 | *ANO1* | down | 1.66 | 3.77E-05 | down | 1.16 | 1.47E-02 | 1 |
| A_24_P153840 | *FGD3* | down | 1.65 | 1.04E-06 | down | 1.20 | 6.03E-03 | 1 |
| A_24_P873598 |  | down | 1.65 | 9.76E-05 | down | 1.09 | 2.51E-02 | 1 |
| A_32_P188921 |  | down | 1.65 | 1.18E-07 | down | 1.39 | 1.08E-02 | 1 |
| A_24_P305597 | *ADRM1* | down | 1.64 | 1.63E-05 | down | 1.48 | 5.46E-03 | 1 |
| A_23_P427754 | *GJD3* | down | 1.64 | 1.82E-03 | down | 1.15 | 1.70E-02 | 1 |
| A_23_P99216 | *POP5* | down | 1.64 | 1.36E-06 | down | 1.40 | 6.02E-03 | 1 |
| A_23_P361820 | *ATG2A* | down | 1.64 | 3.76E-03 | down | 1.16 | 1.30E-03 | 1 |
| A_24_P256603 | *LYRM7* | down | 1.64 | 9.90E-05 | down | 1.16 | 6.23E-03 | 1 |
| A_24_P205604 | *PADI2* | down | 1.63 | 1.79E-02 | down | 1.03 | 2.80E-02 | 1 |
| A_23_P27994 | *TYROBP* | down | 1.63 | 1.28E-02 | down | 1.03 | 7.86E-02 | 1 |
| A_23_P404108 | *FAM190B* | down | 1.63 | 2.02E-06 | down | 1.08 | 7.22E-02 | 1 |
| A_23_P404667 | *BIK* | down | 1.63 | 6.72E-08 | down | 1.34 | 1.97E-02 | 1 |
| A_23_P318284 | *GPD1L* | down | 1.63 | 2.09E-07 | down | 1.27 | 1.11E-02 | 1 |
| A_23_P98092 | *OAT* | down | 1.62 | 2.98E-06 | down | 1.44 | 9.19E-03 | 1 |
| A_23_P370707 |  | down | 1.62 | 5.85E-08 | down | 1.26 | 1.20E-02 | 1 |
| A_24_P753161 | *BMPR2* | down | 1.62 | 3.76E-03 | down | 1.01 | 7.05E-01 | 1 |
| A_23_P27573 | *TFPT* | down | 1.60 | 3.82E-06 | down | 1.42 | 1.55E-03 | 1 |
| A_24_P163574 | *GIGYF1* | down | 1.60 | 2.46E-07 | down | 1.22 | 3.14E-02 | 1 |
| A_24_P595877 | *SFRS12* | down | 1.60 | 4.06E-05 | down | 1.01 | 4.63E-01 | 1 |
| A_23_P202496 | *NOC3L* | down | 1.60 | 2.78E-07 | down | 1.41 | 7.37E-03 | 1 |
| A_24_P205008 | *TSEN54* | down | 1.59 | 1.58E-04 | down | 1.10 | 4.69E-03 | 1 |
| A_23_P253464 | *FAM175A* | down | 1.59 | 9.24E-07 | down | 1.15 | 7.93E-02 | 1 |
| A_24_P281025 |  | down | 1.59 | 1.72E-03 | down | 1.33 | 6.25E-02 | 1 |
| A_24_P361167 | *CHD8* | down | 1.59 | 5.86E-09 | down | 1.29 | 7.20E-02 | 1 |
| A_32_P12430 | *SPG20* | down | 1.58 | 4.94E-06 | down | 1.02 | 3.60E-02 | 1 |
| A_23_P83781 | *CYTH1* | down | 1.58 | 9.92E-08 | down | 1.32 | 2.83E-02 | 1 |
| A_23_P33886 | *TIMM17B* | down | 1.57 | 2.18E-06 | down | 1.26 | 2.80E-02 | 1 |
| A_32_P420009 | *ALS2CL* | down | 1.57 | 9.12E-07 | down | 1.09 | 1.52E-01 | 1 |
| A_23_P153022 | *KRTAP2-4* | down | 1.57 | 1.34E-03 | down | 1.36 | 5.50E-04 | 1 |
| A_23_P255916 | *RPUSD3* | down | 1.57 | 1.97E-05 | down | 1.24 | 5.82E-03 | 1 |
| A_32_P114896 | *PTGES3* | down | 1.57 | 2.66E-07 | down | 1.29 | 3.57E-03 | 1 |
| A_23_P427703 | *MT1L* | down | 1.56 | 4.78E-04 | down | 1.55 | 5.89E-03 | 1 |
| A_32_P192804 |  | down | 1.56 | 4.20E-03 | down | 1.02 | 1.11E-02 | 1 |
| A_32_P200820 |  | down | 1.55 | 6.15E-05 | down | 1.14 | 1.81E-01 | 1 |
| A_23_P111995 | *LOXL2* | down | 1.55 | 2.88E-07 | down | 1.40 | 8.25E-02 | 1 |
| A_24_P361896 | *MT2A* | down | 1.54 | 3.75E-05 | down | 1.28 | 1.72E-01 | 1 |
| A_32_P8952 | *LMBRD2* | down | 1.54 | 3.60E-05 | down | 1.02 | 3.03E-01 | 1 |
| A_23_P413803 | *C5orf37* | down | 1.54 | 1.79E-05 | down | 1.08 | 1.04E-01 | 1 |
| A_23_P27315 | *EMILIN2* | down | 1.54 | 3.55E-04 | down | 1.05 | 3.46E-02 | 1 |
| A_23_P161190 | *VIM* | down | 1.54 | 3.64E-09 | down | 1.39 | 3.69E-04 | 1 |
| A_23_P258972 | *GOLGA1* | down | 1.54 | 5.29E-07 | down | 1.16 | 3.47E-03 | 1 |
| A_23_P66766 | *RPAIN* | down | 1.54 | 1.84E-05 | down | 1.26 | 8.82E-04 | 1 |
| A_23_P311468 | *ZNF428* | down | 1.53 | 2.76E-03 | down | 1.13 | 1.29E-02 | 1 |
| A_24_P795215 |  | down | 1.53 | 1.35E-02 | down | 1.01 | 5.50E-02 | 1 |
| A_23_P128706 | *DYNC1H1* | down | 1.53 | 2.79E-04 | down | 1.40 | 4.19E-03 | 1 |
| A_23_P83579 | *ARNT2* | down | 1.53 | 3.41E-02 | down | 1.02 | 4.74E-02 | 1 |
| A_24_P416059 | *MAN2C1* | down | 1.53 | 8.70E-07 | down | 1.14 | 4.15E-03 | 1 |
| A_23_P161070 | *TOR1AIP1* | down | 1.53 | 2.45E-05 | down | 1.11 | 1.40E-02 | 1 |
| A_23_P111843 | *ZNHIT1* | down | 1.53 | 2.88E-06 | down | 1.36 | 3.45E-03 | 1 |
| A_23_P153086 | *C18orf22* | down | 1.53 | 1.57E-07 | down | 1.25 | 7.10E-02 | 1 |
| A_23_P336728 | *DKFZP564C152* | down | 1.53 | 1.16E-02 | down | 1.02 | 4.45E-01 | 1 |
| A_32_P8971 |  | down | 1.52 | 5.97E-05 | down | 1.14 | 2.75E-02 | 1 |
| A_32_P138213 |  | down | 1.51 | 4.16E-03 | down | 1.06 | 1.64E-01 | 1 |
| A_23_P111797 | *DKFZp434F142* | down | 1.51 | 1.02E-03 | down | 1.10 | 2.96E-03 | 1 |
| A_23_P57306 | *CHAF1B* | down | 1.51 | 2.85E-08 | down | 1.34 | 4.67E-03 | 1 |
| A_23_P108835 | *YPEL5* | down | 1.51 | 2.44E-05 | down | 1.15 | 4.33E-03 | 1 |
| A_32_P54442 | *CHPF2* | down | 1.51 | 6.20E-07 | down | 1.25 | 5.70E-02 | 1 |
| A_23_P336198 | *GLCCI1* | down | 1.50 | 4.31E-06 | down | 1.08 | 3.46E-03 | 1 |
| A_24_P205307 |  | down | 1.50 | 9.82E-03 | down | 1.05 | 1.06E-02 | 1 |
| A_23_P395054 | *ANXA8L2* | down | 1.50 | 3.61E-05 | down | 1.07 | 4.32E-02 | 1 |
| A_23_P118002 | *UQCRC2* | down | 1.50 | 1.47E-06 | down | 1.32 | 3.11E-03 | 1 |
