# Supplementary material for: A dedicated microarray for in-depth analysis of pre-mRNA splicing events: application to the study of genes involved in the response to targeted anticancer therapies
Source: Mol Cancer. 2014 Jan 15;13:9. doi: 10.1186/1476-4598-13-9 (PMC3899606; doi:10.1186/1476-4598-13-9)
Supplement: Additional file 6: Table S5 — Supporting evidences. The list of supporting evidences that confirmed the regulation of some probe sets in SRSF2-over-expressing H358 lung cancer cells in comparison to H358 control cells is presented. [file 1476-4598-13-9-S6.doc]

**Supplementary Table 5.** **Supporting evidences.** The list of supporting evidences that confirmed the regulation of some probe sets in SRSF2-over-expressing H358 lung cancer cells in comparison to H358 control cells is presented.

| Gene Symbol | Region Name | Region Type | Regulation | Fold-Change | P-Value |
| --- | --- | --- | --- | --- | --- |
| *AKT3* | e7 | exon | up | 2.25 | 6.27E-02 |
| *AKT3* | je6_e7 | junction | up | 1.40 | 5.20E-03 |
| *HER1/EGFR* | e18 | exon | up | 1.85 | 9.08E-02 |
| *HIF1A* | je8_e9 | junction | down | 1.39 | 1.36E-03 |
| *HIF1A* | je9_e10 | junction | down | 1.40 | 1.27E-02 |
| *VEGFA* | e4 | exon | down | 1.25 | 6.92E-02 |
| *VEGFA* | je4_ae8_acceptor_alter | junction | down | 1.27 | 9.34E-02 |
| *VEGFA* | jae6_donor_alter_1_e7 | junction | up | 1.42 | 4.14E-03 |
| *VEGFA* | jae7_donor_alter_ae8_acceptor_alter | junction | up | 1.45 | 1.48E-08 |
| *VEGFA* | je7_ae8_acceptor_alter | junction | up | 1.31 | 6.62E-04 |
