# Supplementary material for: A dedicated microarray for in-depth analysis of pre-mRNA splicing events: application to the study of genes involved in the response to targeted anticancer therapies
Source: Mol Cancer. 2014 Jan 15;13:9. doi: 10.1186/1476-4598-13-9 (PMC3899606; doi:10.1186/1476-4598-13-9)
Supplement: Additional file 7: Table S6 — Quantitative RT-PCR validation. Common regulation events between the 15k custom and 44k AgilentTM microarrays were validated by quantitative RT-PCR in SRSF2-over-expressing H358 lung cancer cells in comparison to H358 control cells. Relative mRNA levels were normalized to that of GAPDH. [file 1476-4598-13-9-S7.doc]

**Supplementary Table 6.** **Quantitative RT-PCR validation.** Common regulation events between the 15k custom and 44k Agilent^TM^ microarrays were validated by quantitative RT-PCR in SRSF2-over-expressing H358 lung cancer cells in comparison to H358 control cells. Relative mRNA levels were normalized to that of *GAPDH*.

|  |  | 15k Custom Microarray | | | 44k Commercial Microarray | | | Relative Expression |
| --- | --- | --- | --- | --- | --- | --- | --- | --- |
| Agilent Probe Name | Gene Symbol | Regulation | Fold-Change | P-Value | Regulation | Fold-Change | P-Value |
| A_24_P941947 | *LPCAT1* | up | 5.68 | 2.45E-05 | up | 1.08 | 2.19E-02 | 3.73 |
| A_23_P216068 | *ATAD2* | up | 1.68 | 1.36E-07 | up | 2.01 | 1.05E-01 | 1.50 |
| A_24_P272310 | *MUSTN1* | down | 10.12 | 3.54E-03 | down | 1.07 | 2.88E-03 | 0.24 |
| A_23_P59967 | *RP1* | down | 6.34 | 2.00E-02 | down | 1.05 | 2.04E-02 | 0.33 |
| A_23_P1331 | *COL13A1* | down | 4.89 | 1.59E-09 | down | 1.70 | 4.65E-03 | 0.21 |
| A_23_P88849 | *RRAD* | down | 3.32 | 3.00E-09 | down | 2.51 | 2.54E-03 | 0.40 |
| A_24_P873764 | *BCR* | down | 2.89 | 1.22E-08 | down | 2.15 | 6.16E-05 | 0.46 |
| A_23_P98248 | *TRPT1* | down | 2.87 | 1.69E-10 | down | 2.22 | 8.04E-04 | 0.40 |
| A_23_P395374 | *HIST1H4D* | down | 2.49 | 3.78E-07 | down | 2.02 | 1.85E-03 | 0.66 |
