# Supplementary material for: A dedicated microarray for in-depth analysis of pre-mRNA splicing events: application to the study of genes involved in the response to targeted anticancer therapies
Source: Mol Cancer. 2014 Jan 15;13:9. doi: 10.1186/1476-4598-13-9 (PMC3899606; doi:10.1186/1476-4598-13-9)
Supplement: Additional file 8: Figure S2 — Design of the custom 15k gene chip. The chip was designed on the backbone of the AgilentTM 15k whole-genome microarray. The majority of the probes correspond to custom oligonucleotides, i.e. to both known and predictable sequences of exons, introns and junctions of 16 genes selected for their biological interest in the response to targeted anticancer therapies: AKT1-3, HER1-4, HIF1A, PIK3CA, PIK3R1-2, VEGFA-D and PIR. The resolution of the custom microarray was decreased in comparison to the Human Exon 1.0 ST array (AffymetrixTM) from 5 million to 12,000 probes, but the number of probes per gene was largely increased, from an average of 45 to an average of 185 probes per gene. The expression of 1,967 distinct genes can also be analyzed thanks to commercial AgilentTM probes. [file 1476-4598-13-9-S8.ppt]

## Slide 1
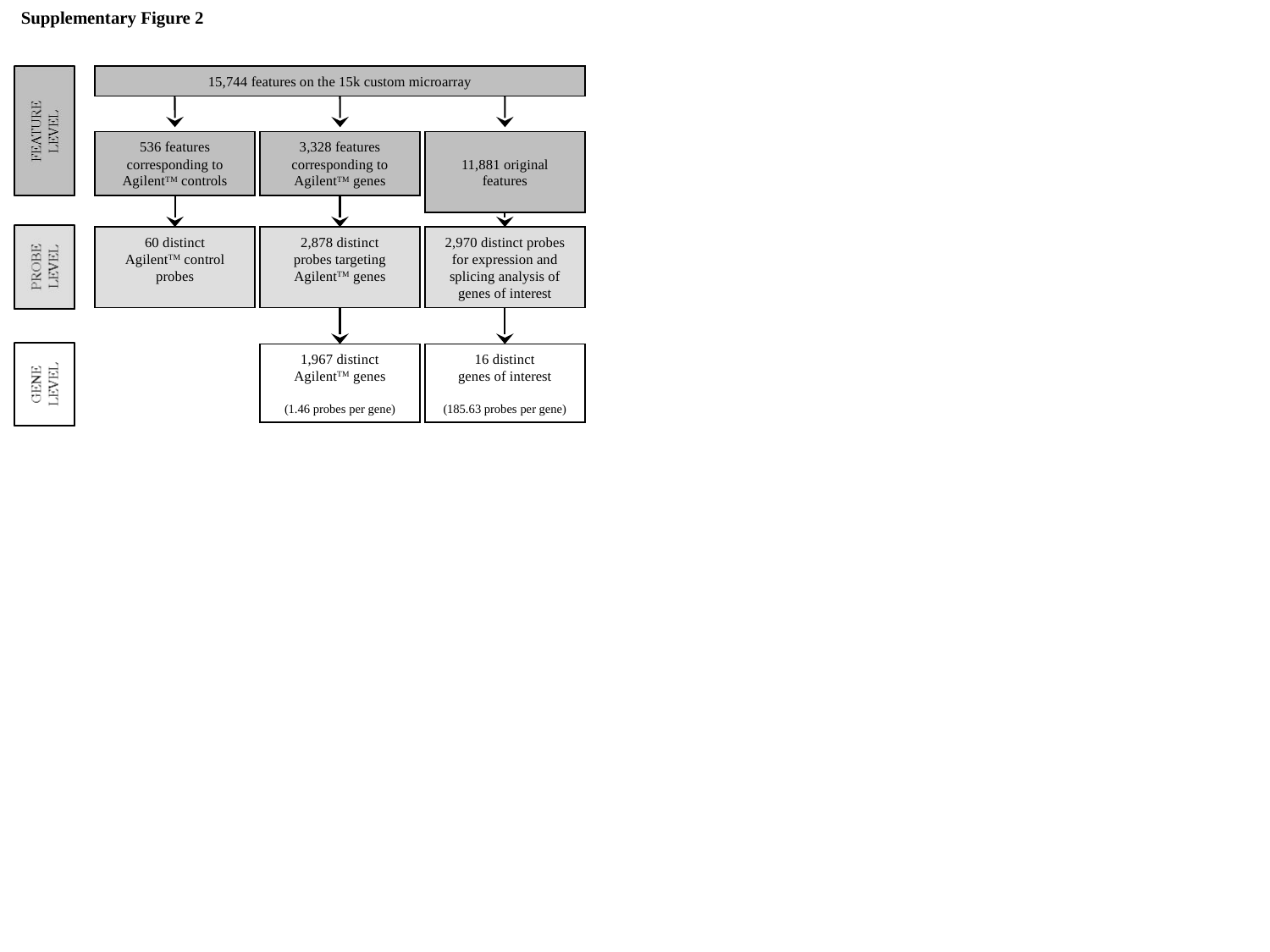

Supplementary Figure 2
15,744 features on the 15k custom microarray
536 features corresponding to AgilentTM controls
3,328 features corresponding to AgilentTM genes
11,881 original features
60 distinct
AgilentTM control probes
2,878 distinct
probes targeting
AgilentTM genes
2,970 distinct probes for expression and splicing analysis of genes of interest
1,967 distinct AgilentTM genes
(1.46 probes per gene)
16 distinct
genes of interest
(185.63 probes per gene)
