# Supplementary material for: A dedicated microarray for in-depth analysis of pre-mRNA splicing events: application to the study of genes involved in the response to targeted anticancer therapies
Source: Mol Cancer. 2014 Jan 15;13:9. doi: 10.1186/1476-4598-13-9 (PMC3899606; doi:10.1186/1476-4598-13-9)
Supplement: Additional file 10: Table S7 — Primers for validation. The sequences of the primers used for the 15k custom microarray validation are presented. [file 1476-4598-13-9-S10.doc]

**Supplementary Table 7.** **Primers for validation.** The sequences of the primers used for the 15k custom microarray validation are presented.

| Gene Symbol | Tested event | Sense primer | Sense primer sequence (5'->3') | Antisense primer | Antisense primer sequence (5'->3') | Expected size (bp) |
| --- | --- | --- | --- | --- | --- | --- |
| *AKT3* | e7+/e8+ | AKT3_je7-e8s | GCGAGCTGTTTTTCCATTTG | AKT3_e9as | CTGGTGCCAGATATTCTGGAGT | 255 |
| *AKT3* | e7+/e8- | AKT3_je7-e9s | GGGCGAGTTGGAGAATCTAA | AKT3_e9as | CTGGTGCCAGATATTCTGGAGT | 134 |
| *AKT3* | e7-/e8+ | AKT3_je6-e8s | CATCCCTTTTTAACACTGTTTTTC | AKT3_e9as | CTGGTGCCAGATATTCTGGAGT | 265 |
| *AKT3* | e7-/e8- | AKT3_je6-e9s | CATCCCTTTTTAACATTGGAGAA | AKT3_e9as | CTGGTGCCAGATATTCTGGAGT | 142 |
| *HER1/EGFR* | last exon = e17 | HER1_e16s | ACAACACCCTGGTCTGGAAG | HER1_e17as | TGAAGCAAAGGGAGAAATTGA | 159 |
| *HER1/EGFR* | last exon = e18 | HER1_e16s | ACAACACCCTGGTCTGGAAG | HER1_e18as | TCCTAAGCATGACTCCTGAGC | 200 |
| *HER1/EGFR* | last exon > e20 | HER1_e16s | ACAACACCCTGGTCTGGAAG | HER1_e20as | ATGGCCTTGGCACTCAAC | 200 |
| *HIF1A* | e9+/e10+ | HIF1A_je9-e10s | GTGAGTGGTATTATTCAGCACGA | HIF1A_e11as | TTGATTGAGTGCAGGGTCAG | 393 |
| *HIF1A* | e9+/e10- | HIF1A_je9-e11s | ACGTTGTGAGACACAGAAACTG | HIF1A_e11as | TTGATTGAGTGCAGGGTCAG | 177 |
| *HIF1A* | e9-/e10+ | HIF1A_je8-e10s | CTCATCATGATATGGTATTATTCAGC | HIF1A_e11as | TTGATTGAGTGCAGGGTCAG | 400 |
| *HIF1A* | e9-/e10- | HIF1A_je8-e11s | CATGATAACACAGAAACTGATGACC | HIF1A_e11as | TTGATTGAGTGCAGGGTCAG | 174 |
| *VEGFA* | last exon = e4 | VEGFA_e4s | TTATGCGGATCAAACCTCAC | VEGFA_ae4as | GCTGATAATGAATCCGTGACTAC | 107 |
| *VEGFA* | last exon > e5 | VEGFA_e4s | TTATGCGGATCAAACCTCAC | VEGFA_e5as | CTTGTCTTGCTCTATCTTTCTTTGG | 102 |
| *VEGFA* | alternative donor e6 | VEGFA_ae6s | CTGCTGTCTAATGCCCTGGA | VEGFA_e7as | AAATGCTTTCTCCGCTCTGA | 72 |
| *VEGFA* | constitutive donor e6 | VEGFA_je6-e7s | AAGAAATCCCGTCCCTGTG | VEGFA_e7as | AAATGCTTTCTCCGCTCTGA | 47 |
